# Transition Metal-Catalyzed Direct C(sp^3^)–H Functionalization Reactions of Aliphatic Carboxylic Acids

**DOI:** 10.1021/acs.chemrev.5c01044

**Published:** 2026-04-10

**Authors:** Sourjya Mal, Manuel van Gemmeren

**Affiliations:** † Otto Diels-Institut für Organische Chemie, 9179 Christian-Albrechts-Universität zu Kiel, Otto-Hahn-Platz 4, 24118 Kiel, Germany

## Abstract

The selective functionalization of C–H bonds is a cornerstone of contemporary organic synthesis. Carboxylic acids, which range among the most abundant functional groups in nature and manmade molecules, find widespread applications in multiple disciplines. Consequently, substantial research has been devoted to using the weakly coordinating carboxylate group to direct the selective functionalization of C–H bonds. In this context, the activation of C–H bonds has emerged as a powerful strategy to achieve the site-selective functionalization of unactivated C­(sp^3^)–H bonds with diverse coupling partners. Alternatively, radical-mediated approaches have delivered complementary mode of reactivity. This review provides an overview of transition metal-catalyzed C­(sp^3^)–H functionalization reactions, enabled by the carboxylic acid group, spanning contributions from 1991 to 2025. It thoroughly examines the distinct mechanistic manifolds for the C–H functionalization of carboxylic acids, systematically comparing these modes of reactivity and their respective challenges/opportunities. In addition, the discussion encompasses reaction design, substrate scopes, mechanistic features, and a critical assessment of the current methodologies, providing readers with valuable insights to guide future research efforts.

## Graphical abstract

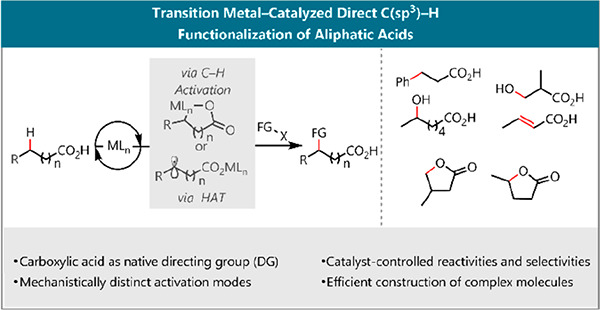

## 1 Introduction

Carboxylic acids are among the most widespread and versatile structural motifs in organic synthesis.^1^ They are commonly found in natural products,^2^ and serve as fundamental building blocks for numerous essential molecules, such as amino acids, keto-acids, fatty acids, and carbohydrate-derivatives. The wide commercial availability in diverse structural forms render them highly useful as starting materials, synthetic intermediates, and products.^3,4^ Moreover, they have numerous industrial applications, including the production of polymers, solvents, food additives, pharmaceuticals, and soaps.^5–7^ Carboxylic acids can be readily synthesized on a laboratory scale via oxidation of primary alcohols or aldehydes and can be further transformed into various derivatives, such as carboxylate salts, esters, amides, acid chlorides, and alcohols. Besides these classical transformations, several strategies have recently emerged which use either the carboxylic acid group or its redox-active ester derivatives for decarboxylative functionalizations.^8–11^ Complementing these strategies, the direct C–H functionalization of carboxylic acids through various activation modes has attracted substantial attention and is at the center of this review.^12–17^ It should be noted that the development of new methods for carboxylic acid directed C­(sp^2^)–H functionalization is of broad importance, and the substantial progress achieved in this area over the past decades has been thoroughly summarized in previous reviews.^12,18^ This review surveys transition metal–catalyzed C­(sp^3^)–H functionalization reactions directed by carboxylic acids, covering developments from 1991 through summer 2025.

## 2 C–H Functionalization Reactions of Aliphatic Acids via C–H Activation

Complementing the above-mentioned transformations, transition metal-catalyzed C–H activation reactions are recognized as a powerful tool for rapidly building molecular complexity in an atom- and step-economic fashion. While this strategy has been proven highly effective for C­(sp^2^)–H activation reactions using carboxylic acid as directing group,^12,19^ the analogous C­(sp^3^)–H activation of aliphatic carboxylic acids long remained underexplored. C­(sp^3^)–H bonds are abundant in organic molecules, but their functionalization is inherently challenging, primarily due to the low polarity, low acidity (estimated p*K*_*a*_ = 45–60), and lack of proximal empty low-energy or filled high-energy orbitals that could readily interact with orbitals of a metal catalyst.^20–25^ Moreover, the relatively weak nature of the metal–alkyl bond formed in the C–H activation step introduces an additional obstacle.^26^ To overcome these challenges, the metal catalyst must be positioned close to the target C–H bond, which is generally accomplished using directing groups (DGs). Precoordination of the metal to a directing group guides it near the desired C–H bond, a phenomenon known as the complexation-induced proximity effect (CIPE).^27–29^ CIPE not only accelerates the reaction but also controls the positional selectivity. The CIPE induced C–H activation operates via a “deprotonation”-like pathway, and consequently the relative reactivity of C­(sp^3^)–H bonds follows the order primary (1°) > secondary (2°) > tertiary (3°), consistent with their intrinsic C–H acidity. The carboxylic acid moiety can coordinate to the metal with two modes: monodentate (κ^1^) or bidentate (κ^2^).^30–33^ In the κ^2^ mode, both oxygen atoms bind to the metal, while in the κ^1^ coordination only one oxygen interacts with metal (Scheme 1A). These modes exist in equilibrium, slightly favoring the unproductive κ^2^ coordination, but the presence of Lewis acidic metal ions, such as Na^+^, K^+^, or Cs^+^ can shift the equilibrium toward the κ^1^ coordination mode by themselves interacting with the carboxylate in a bidentate fashion. Even when κ^1^ coordination is achieved, the free rotation of single bonds between the acid functionality and the C­(sp^3^)–H bonds to be activated imparts significant conformational flexibility, making it entropically challenging to adopt geometries required for C–H activation. This challenge is particularly severe for α-nonquaternary acids (when R^1^ and/or R^2^ = H). In contrast, α-quaternary acids benefit from the Thorpe-Ingold effect, which reduces the entropic penalty for the system to adopt a conformation that places the C–H bond in immediate proximity to the catalyst.^34^ Following preorganization of the reactive conformer, carboxylic acid directed C–H activation leads to formation of a metallacycle, typically five or six membered. In Pd-catalyzed C­(sp^3^)–H activation, β-C­(sp^3^)–H activation typically generates a five-membered palladacycle that is both kinetically and thermodynamically favored over the corresponding six-membered metallacycle formed via γ-C­(sp^3^)–H activation.^35–39^ In contrast, larger metals such as platinum have been reported to favor product formation through six-membered metallacycles, which are considered less strained due to the larger metal center.^40,41^

The resulting cyclometalated species can then engage with suitable coupling partners to introduce the desired functional groups at the targeted site. However, these intermediates are prone to undergo competing side reactions, including β-hydride elimination (when R^1^ and/or R^2^ = H), and homocoupling which can reduce the overall efficiency of the process.^42^ Collectively, these factors render aliphatic carboxylic acid substrates notoriously challenging for C­(sp^3^)–H activation processes. Previously the C­(sp^3^)–H activation/functionalization largely relied on the conversion of carboxylic acid into monodentate^43–45^ or bidentate exogeneous directing groups (DGs) (Scheme 1B),^42,45–53^ which enabled successful activation and subsequent functionalization of C­(sp^3^)–H with a wide range of coupling partners. Despite their effectiveness, these strategies have several inherent limitations. The installation and removal of the directing group adds extra steps, reducing both the step- and atom-economy of the process, and in some cases, the DGs can become a permanent part of the product. Strong DGs tend to produce cyclometalated intermediates which are thermodynamically stable but less reactive to the subsequent functionalization. Additionally, the strong chelating ability of bidentate DGs can predominantly occupy the metal’s coordination sphere, outcompeting external ligands that might otherwise enhance enantio- and regioselectivity and accelerate the entire process. In this context, the use of the free carboxylic acid moiety as a native directing group is particularly attractive. The weak coordination enhances the reactivity of the thermodynamically less stable palladacycle intermediates, compared to strong DG-derived counterparts, allowing for more efficient coupling with various reaction partners. Furthermore, it also leaves open coordination sites for external ligands, improving selectivity and overall reaction efficiency. Despite the challenges outlined above, substantial advancement has been made in transition metal-catalyzed C­(sp^3^)–H activation and functionalization of aliphatic carboxylic acids over the past few years. It should be noted that the term “C–H activation” used in this review refers specifically to C–H functionalization reactions that involve a direct carbon–metal bond formation via an inner-sphere mechanism.^54^

**Scheme 1:**
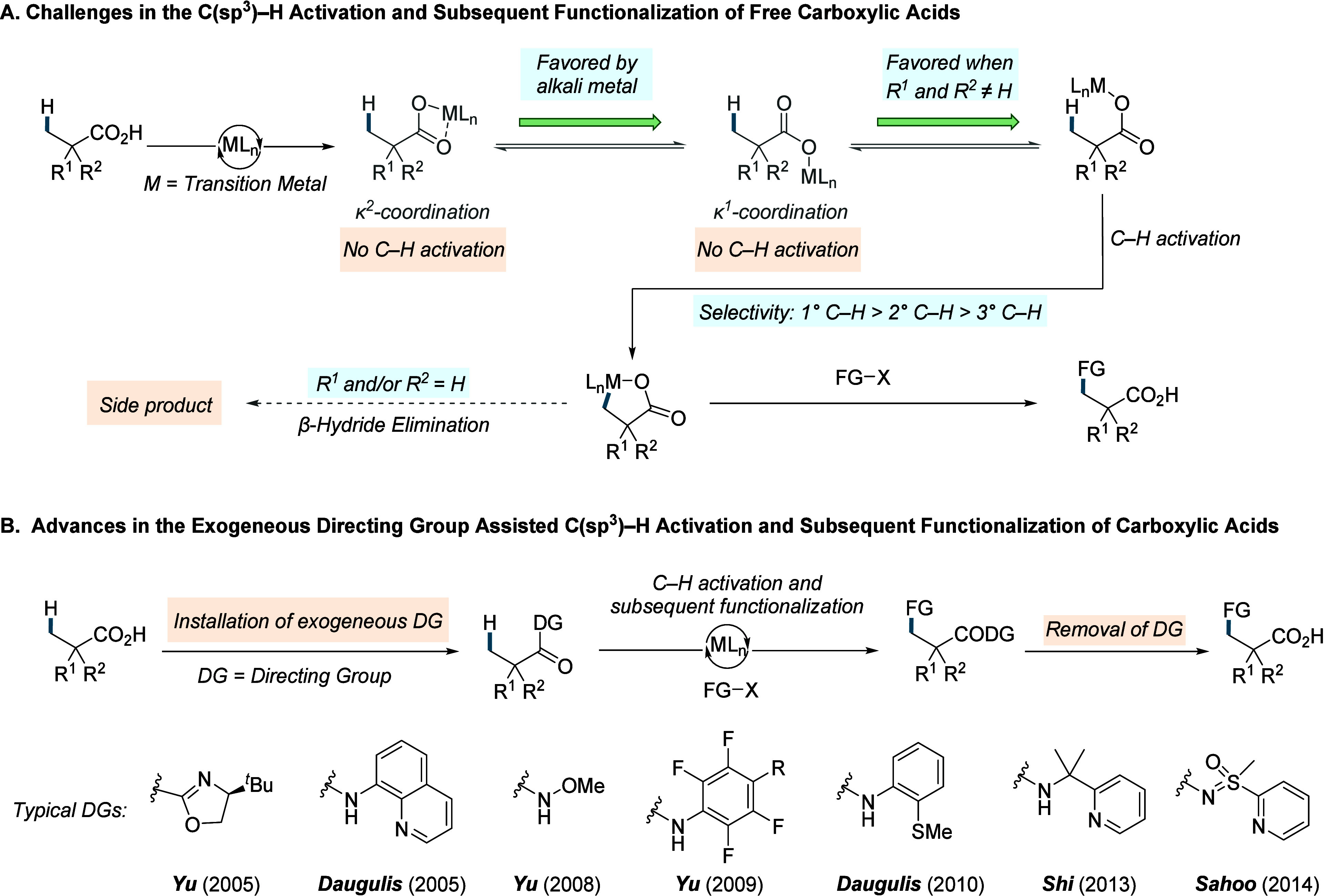
A. Challenges Associated with the C­(sp^3^)–H Activation/Functionalization of Free Carboxylic Acids; B. Recent Progress in the Exogeneous Directing Group Assisted C­(sp^3^)–H Activation and Subsequent Functionalization of Carboxylic Acids

### 2.1 Carbon–Carbon Bond Forming Reactions

#### 2.1.1 β-Methyl-C­(sp^3^)–C Bond Forming Reactions

Following reports by Kao and Sen on intramolecular Pt-catalyzed γ-C­(sp^3^)–H lactonizations,^40^ in 2007, Yu et al. reported the first intermolecular C­(sp^3^)–H bond activation and functionalization using a carboxylic acid as a native directing group (Scheme 2).^55^ The authors showed that α-quaternary carboxylic acids (**1**) can be arylated using organoboron reagents (**2**). The catalytic system involves a combination of Pd­(OAc)_2_, benzoquinone (BQ), Ag_2_CO_3_, and K_2_HPO_4_, which afforded a range of predominantly monoarylated acids (**3a**–**3d**, Scheme 2A) in moderate yields.

**Scheme 2:**
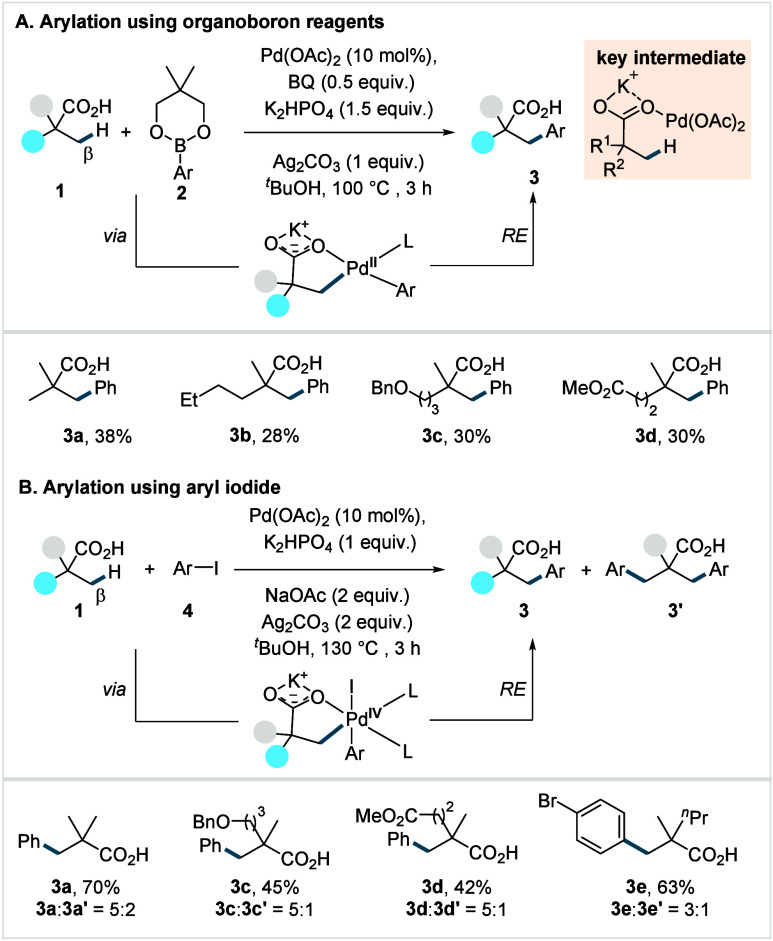
Yu et al. (**2007**): Direct β-C­(sp^3^)–H Arylation of α-Quaternary Carboxylic Acids

The reaction proceeds via a Pd^II^/Pd^0^ cycle, in which the initially formed C–H activated palladacycle undergoes transmetalation with **2**, transferring the aryl group to Pd^II^. Subsequent reductive elimination (RE) delivers **3** with concomitant generation of Pd^0^, which is then reoxidized to Pd^II^ by terminal oxidants (Ag-salt, BQ). Furthermore, the authors also employed aryl iodide (**4**) as arylating reagent under modified reaction conditions (Scheme 2B), which afforded the desired arylated acids (**3a**–**3e**) in moderate to good yields, albeit as a mixture of mono- and diarylated acids. In this case, the reaction is proposed to proceed via a Pd^II^/Pd^IV^ cycle. After the C–H activation step, Pd^II^ undergoes oxidative addition (OA) with **4** to generate high-valent Pd^IV^ intermediate. This species then liberates the product **3** via RE with concomitant generation of a Pd^II^IL_n_ species, from which the active catalyst is regenerated by anion exchange with either silver salt or acetate. The formation of diarylated product was attributed to the fact that Pd^II^ remains coordinated to carboxylate after the first arylation, resulting in iterative arylation. The key discovery that enabled this study was the addition of external salts including Lewis-acidic Na^+^ and K^+^ cations. These additives substantially enhanced the reactivity by shifting the κ^1^- to κ^2^- coordination equilibrium toward the required κ^1^-coordination for palladium as described in previous section (Scheme 1A).

In 2017, The groups of Yu and Zhao simultaneously reported the β-C­(sp^3^)–H arylation of *N*-phthalimide-protected amino acids to obtain unnatural *N*-protected arylated amino acids. Yu and co-workers developed a catalytic system based on a pyridine ligand fused with five-membered ring (**L1**, Conditions A, Scheme 3),^56^ whereas Zhao et al. employed *N*-acetyl glycine (Ac-Gly-OH) as ligand (Conditions B, Scheme 3).^57^ In both cases, a Pd^II^/Pd^IV^ catalytic cycle analogous to the previously discussed mechanism (Scheme 2B), is proposed to be operative. The use of 1,1,1,3,3,3-hexafluoroisopropanol (HFIP) was found to be crucial solvent for the reaction, presumably due to its unique combination of properties, such as high polarity, low nucleophilicity, and ability of H-bond donation by dimeric and trimeric solvent aggregates.^58–60^ Both studies demonstrated a broad scope of arene coupling partners, affording a range of synthetically useful functional group-containing arylated unnatural amino acids, including methoxy (**6a**), trifluoromethyl (**6b**) in moderate to good yields.

**Scheme 3:**
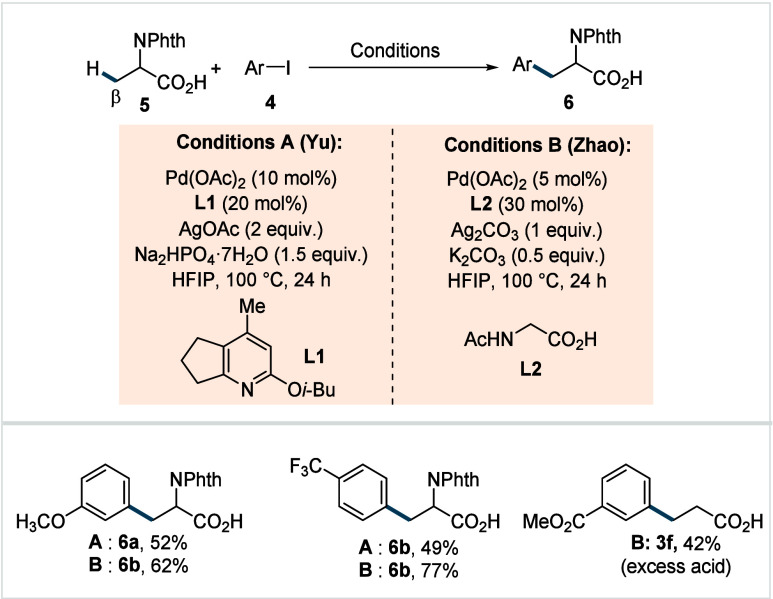
Yu and Zhao et al. (**2017**): Direct β-C­(sp^3^)–H Arylation of *N*-Phthalimide-Protected Carboxylic Acids

However, simple α-nonquaternary aliphatic acid substrates showed poor reactivity (32–34% yield) under Yu’s catalytic system, which could be attributed to the lack of an accelerating Thorpe–Ingold effect and the tendency of C–H activated palladacycle to undergo decomposition via β-hydride elimination as discussed in Section 2 (Scheme 1A). Zhao and co-workers observed that the challenges associated with such substrates could partially be addressed by employing carboxylic acid as an excess reagent (**3f**, 42%).

In parallel to the above-mentioned studies, van Gemmeren et al. developed a catalytic system particularly suited for the β-C­(sp^3^)–H arylation of aliphatic acid substrates (Scheme 4).^61^

**Scheme 4:**
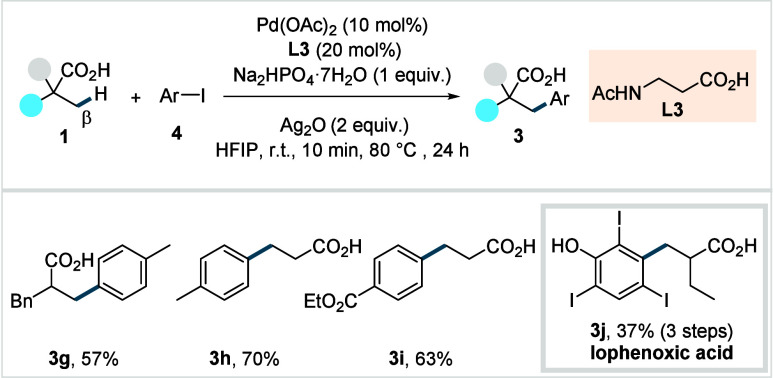
van Gemmeren et al. (**2017**): Direct β-C­(sp^3^)–H Arylation of Aliphatic Carboxylic Acids

This protocol employed carboxylic acid substrates as limiting reagent and is applicable not only to α-quaternary and α-nonquaternary acids, but also to the particularly challenging propionic acid. The reaction showed a broad functional group tolerance toward both acid substrates and aryl iodides, delivering arylated products (**3g**–**3i**) in good to excellent yields. The use of Ac-β-Ala-OH as a ligand (**L3**), which forms a six-membered chelate with the Pd-center, was found to be crucial for achieving the desired reactivity. The utility of their protocol was further demonstrated through the synthesis of iophenoxic acid (**3j**), formerly marketed as a radiopaque agent for X-ray imaging.

More recently, an imidazoline-based ligand was reported to enable the β-C­(sp^3^)–H arylation of α-nonquaternary acids; however, it should be noted that the studies claim to describe the first acid-limited method for α-nonquaternary acids is incorrect in light of the above-mentioned works.^62^

In 2020, Yu and co-workers extended the β-C­(sp^3^)–H arylation of aliphatic acids to DNA-tethered (het)­aryl iodides.^63^ Utilizing water as solvent and an ethylenediamine-derived ligand (**L4**, Scheme 5), the protocol furnished a diverse library of DNA-encoded (het)­arylated aliphatic acids (**3k**–**3m**) in good to excellent yields.

**Scheme 5:**
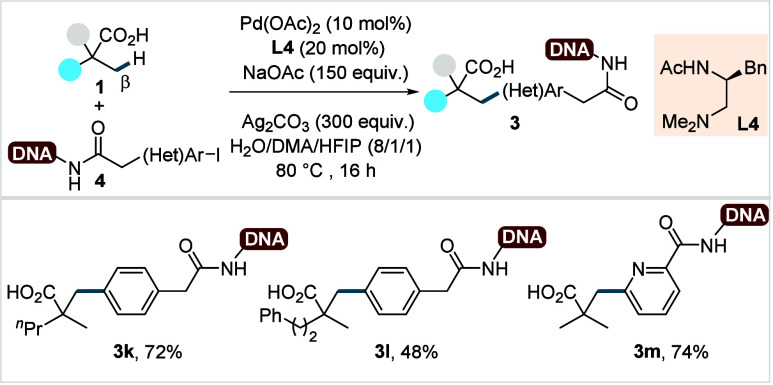
Yu et al. (**2020**): Direct β-C­(sp^3^)–H Arylation of Aliphatic Carboxylic Acids with DNA-Encoded Aryl Iodides

Iterative arylation of primary arylated acid was also demonstrated, enabling access to structurally complex DNA-tagged scaffolds. However, α-nonquaternary acids displayed moderate reactivity under these conditions.

Despite significant advances in the β-C­(sp^3^)–H arylation of aliphatic carboxylic acids, the existing methods remained ineffective for heteroaryl iodides. In 2022, Yu and co-workers developed a catalytic system based on a pyridine-pyridone ligand (**L5**, Scheme 6) to address this shortcoming.^64^ A broad range of heteroarylated aliphatic acids (**3n**–**3q**) was obtained in good to excellent yields. The bidentate pyridine-pyridone ligand was proposed to facilitate the C–H activation by mitigating the nonproductive coordination of the heteroaryl iodides Lewis-basic nitrogen atom to the Pd-center.

**Scheme 6:**
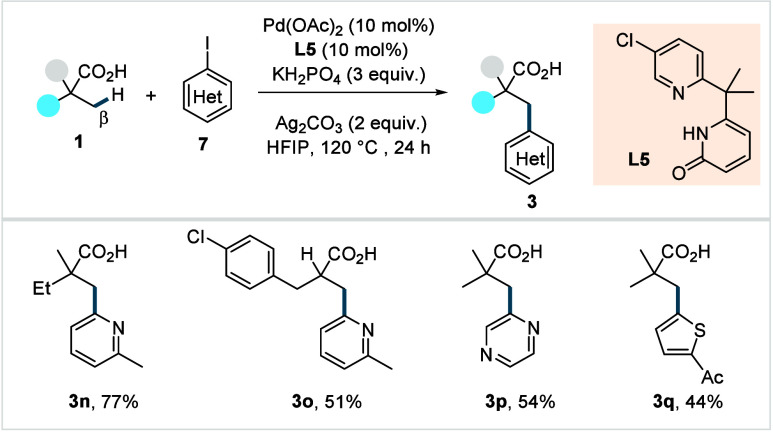
Yu et al. (**2022**): Direct β-C­(sp^3^)–H Heteroarylation of Aliphatic Carboxylic Acids

Preliminary mechanistic studies indicated that while monoheteroarylated acid undergoes C–H activation, the subsequent steps (i.e., OA and RE) are disfavored, affording exclusive monoselectivity for the α-quaternary acids. Furthermore, the protocol enabled a modular synthetic strategy in which sequential arylation and heteroarylation provided a diverse library of di- and trifunctionalized aliphatic acid architectures.

The mechanistic investigation by Lei and co-workers provided further insight into the origin of the monoselectivity observed in the Yu’s β-heteroarylation reaction (Scheme 6).^65^ Their results suggested that in a potential second heteroarylation step, the Pd^IV^ species generated via oxidative addition is exceptionally stabilized through coordination of the additional pyridine moiety in the monoheteroarylated substrate, rendering it reluctant to undergo subsequent reductive elimination, thus preventing the diheteroarylation.

In 2021, The Yu’s group reported a Pd­(II)-catalyzed cyclative C­(sp^3^)–H/C­(sp^2^)–H coupling to access biologically relevant scaffolds, including tetralines, chromanes, and indanes.^66^ The protocol employed a *cis*-cyclopentane-based ligand (**L6**, Scheme 7) and inexpensive sodium percarbonate (Na_2_CO_3_·1.5H_2_O_2_) as the sole oxidant. The superior reactivity of **L6** was proposed to arise from the cyclopentane linkage, which imparts greater conformational rigidity. The reaction is proposed to proceed via a Pd^II^/Pd^IV^ cycle, in which the initially formed C–H activated palladacycle, generated via β-C­(sp^3^)–H activation, undergoes oxidative addition with sodium percarbonate to form a high-valent palladium intermediate. Subsequent C­(sp^2^)–H activation results in the formation of **Int-1** (Scheme 7), which undergoes reductive elimination to deliver **9.** A diverse range of indanes (**9a**), tetralines (**9b**), and chromanes (**9c**) was obtained in good yields.

**Scheme 7:**
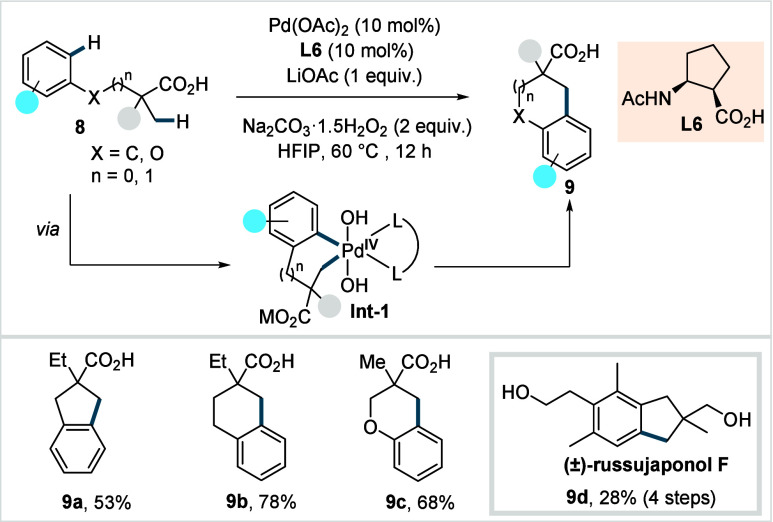
Yu et al. (**2021**): Pd­(II)-Catalyzed Cyclative C­(sp^3^)–H/C­(sp^2^)–H Coupling Reaction of Aliphatic Acids

Chromanes bearing electron-poor arenes, however, displayed moderate reactivity, presumably due to the sluggish nature of the C­(sp^2^)–H activation with electron-deficient arenes. The methodology was further demonstrated in the total synthesis of (±)-russujaponol F (**9d**) via multiple C–H functionalizations over four steps from readily available pivalic acid and phenyl acetic acid.

In the same year, Liu and Shi et al. independently disclosed a method for accessing a diverse range of chromane-3-carboxylic acid and tetralin derivatives through cyclative C­(sp^3^)–H/C­(sp^2^)–H coupling. The transformation proceeds via a Pd^II^/Pd^IV^ catalytic cycle, using TBHP as the oxidant. Notably, a 1:1 combination of an amino acid-derived ligand and a pyridine-derived ligand was found to be essential for achieving the desired reactivity.^67^

In 2018, Yu et al. reported desymmetrization of geminal dimethyl groups to achieve an enantioselective arylation of *N*-phthalimide protected 2-aminoisobutyric acid.^68^ The reaction employed a chiral bidentate *N*-acetyl aminoethyl-derived ligand (**L4**, Conditions A, Scheme 8) and afforded a range of arylated α-amino acid derivatives in good yields (**11a**–**11c**). A range of aryl iodides including the particularly challenging heteroaryl iodides were amenable under this protocol, enabling access to a broad spectrum of non-natural chiral amino acid derivatives (**11a**–**11c**).

**Scheme 8:**
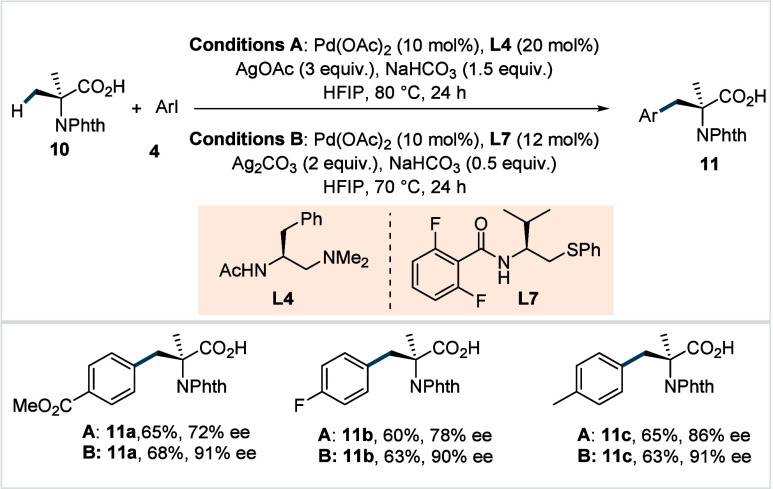
Yu et al. (**2018**): Pd­(II)-Catalyzed Enantioselective C­(sp^3^)–H Arylation of α-Aminoisobutyric Acid

The key rationale for choosing tertiary amines as σ-donors over quinoline- and oxazoline-type motifs lies in their distinct spatial arrangement and the more diffuse lone pair of the sp^3^-hybridized nitrogen relative to that of sp^2^-hybridized nitrogen. However, the reaction showed limited functional group compatibility, and moderate enantioselectivities were observed particularly with the electron-withdrawing aryl iodides. Later, the same group developed a chiral thioether-derived ligand (**L7**, Conditions B, Scheme 8), which substantially improved both the enantioselectivities and substrate scope of the previous method.^69^

Olefins represent one of the most versatile building blocks in organic synthesis. Traditional Heck coupling for forming C­(sp^3^)–(sp^2^) bonds are often challenged by the need for substrate prefunctionalization and the risk of premature β-hydride elimination from alkyl halide. In this context, the direct olefination via C­(sp^3^)–H activation using native directing group offers a valuable alternative. In 2018, Yu and co-workers reported the first β-C­(sp^3^)–H olefination of free carboxylic acids, enabled by a bidentate thioether-based ligand (**L8**, Scheme 9).^70^ The kinetic studies revealed that ligand **L8**, featuring sulfur as a soft σ-donor, substantially accelerates the initial reaction rate and enhances the stability of the Pd catalyst over time, thereby enabling higher turnover numbers.

**Scheme 9:**
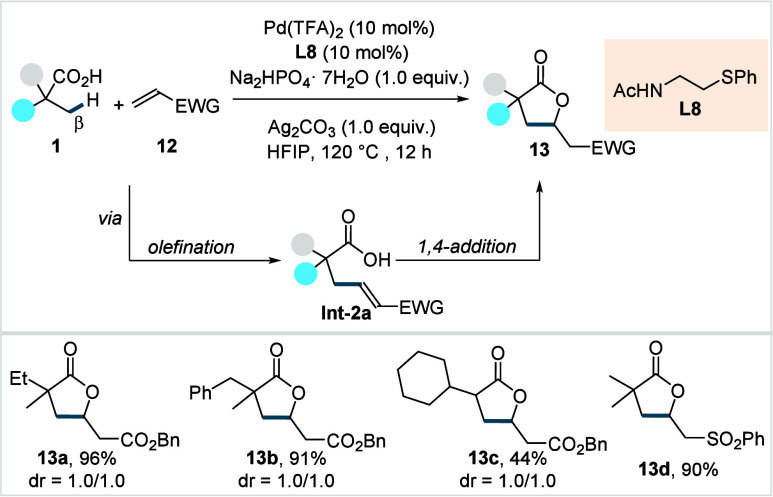
Yu et al. (**2018**): The Direct β-C­(sp^3^)–H Olefination of Aliphatic Acids

The reaction proceeds via a Pd^II^/Pd^0^ cycle to give olefinated acid (**Int-2a**), which undergoes subsequent 1,4-addition to furnish lactone **13**. Both α-quaternary and α-nonquaternary acids were amenable to this protocol, affording the corresponding lactones in moderate to excellent yields (**13a**–**13c**). A diverse range of activated olefins (**12**) bearing functional groups such as ester, sulfone (**13d**), and nitrile were compatible with their protocol. Furthermore, the authors performed gram scale synthesis of the lactone and demonstrated further derivatization into synthetically useful synthons, including β-olefinated and γ-hydroxylated aliphatic acids.

Later, the same group used thioether-based ligand (**L8**, Scheme 10) for the construction of bicyclo[3.2.1]­lactone scaffolds.^71^ Their protocol transformed a range of linear carboxylic acids possessing tethered olefins into bicyclo[3.2.1]­lactone motifs via a sequence of intramolecular β-C­(sp^3^)–H olefination (**Int-2b**) and lactonization. The reaction featured a broad functional group tolerance, affording the respective lactones, including piperidine and tetrahydropyran bicyclo[3.2.1]­lactones (**15a**–**15c**) in good to excellent yields. The method was extended to form bicyclo[4.2.1]­lactone (**15d**) motif. It was found that both (*Z*)- and (*E*)-alkene isomers furnished the desired lactone in comparable yields. The authors showcased the synthetic potential of this approach by synthesizing 6,6,5-tricyclic lactone core of the meroterpenoid cochlactone A in seven steps.

**Scheme 10:**
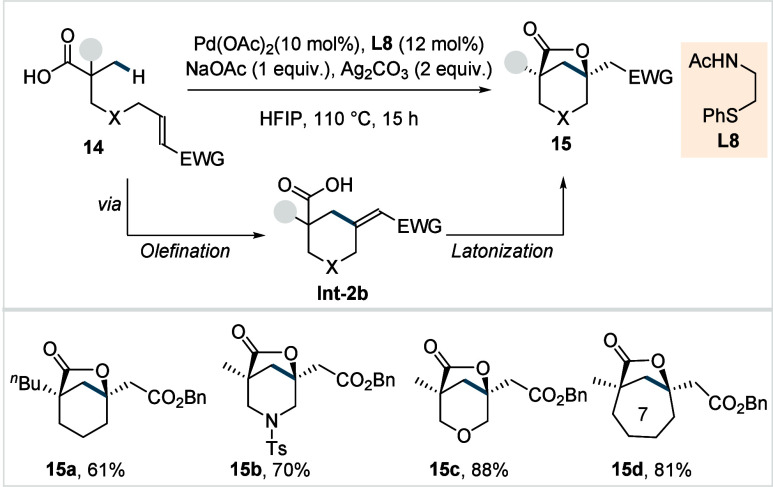
Yu et al. (**2022**): Synthesis of Bicyclo[3.2.1]­lactones via Direct β-C­(sp^3^)–H Olefination of Aliphatic Acids

Jeganmohan and co-workers recently reported the olefination of carboxylic acids (**1**) with a range of allyl electrophiles (**16**–**18**) using thioether-derived ligand (**L8**, Scheme 11).^72^ The transformation proceeded through a sequential olefination and 1,4 addition with allyl alcohols (**16**), affording saturated γ-lactones (**19a**). Allyl acetates (**17**) enabled the formation of γ-alkenylated lactones (**20a**, *E*/*Z* = 5:1) via β-acetoxy elimination, and analogous scaffolds (**21a**–**21b**) were obtained from other allyl electrophiles (**18**) through β-hydride elimination under slightly modified conditions. The protocol showed a broad functional group tolerance, although the substrate scope of carboxylic acids was predominantly limited to α-quaternary acids.

**Scheme 11:**
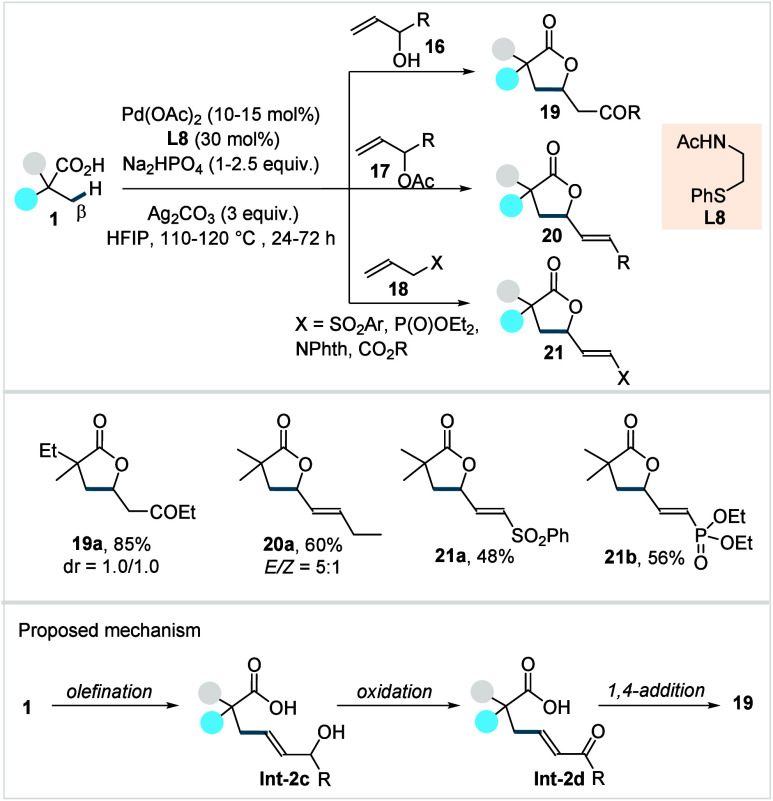
Jeganmohan et al. (**2024**): Pd­(II)-Catalyzed β-C­(sp^3^)–H Functionalization of Aliphatic Acids with Allyl Electrophiles

Mechanistically, the reaction was proposed to proceed via a Pd^II^/Pd^0^ catalytic cycle, in which the olefinated acid (**Int-2c**) is first formed via a β-C­(sp^3^)–H activation. This intermediate subsequently undergoes oxidation in the presence of Pd/Ag/air to generate **Int-2d**, which then forms the product (**19**) via a 1,4-addition. Control experiments further suggest that the in situ-generated α,β-unsaturated ketone, derived from oxidation of allyl alcohol (**16**), can also act as the olefinating agent.

In 2025, van Gemmeren et al. reported a protocol for the synthesis of γ-alkylidene lactones via a sequential olefination and lactonization.^73^ With the aid of a *N*-acyl sulfonamide-derived ligand (**L9**, Scheme 12), the method enabled access to a diverse library of γ-alkylidene lactones from readily available aliphatic acids and olefins through the sequential functionalization of three C–H bonds. The reaction was proposed to proceed via Pd^II^/Pd^0^ cycle, wherein the initially formed olefinated acid (**Int-2e**, Scheme 12) undergoes *anti*-oxypalladation followed by β-hydride elimination to afford the desired lactone **23**.

**Scheme 12:**
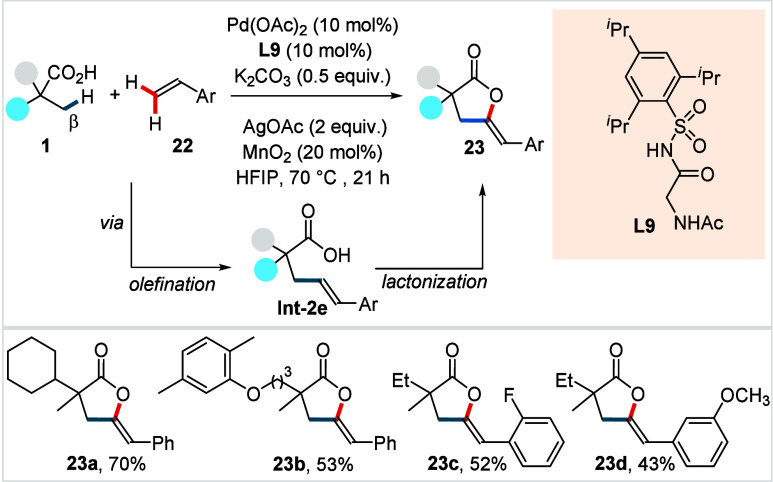
van Gemmeren et al. (**2025**): Synthesis of γ-Alkylidene Lactones via Molecular Stitching of Aliphatic Acids and Olefins

The protocol exhibits a broad compatibility with α-quaternary aliphatic acids and various styrenes (**22**) were well-tolerated by the protocol, resulting the respective lactones (**23a**–**23d**) in moderate to good yields. However, α-nonquaternary acids showed poor reactivity with this protocol. The authors showed the synthetic utility of the products by transforming them into a range of synthetically useful motifs.

The alkyne moiety serves as a versatile synthetic linchpin due to its tremendous synthetic versatility. In 2020, van Gemmeren and co-workers reported the first example of β-C­(sp^3^)–H alkynylation using 1-bromo-2-(triisopropylsilyl)­acetylene (**24**) as the alkynylating reagent (Scheme 13).^74^ The reaction was proposed to proceed via a rate-limiting β-C­(sp^3^)–H activation step, from which either a Pd^II^/Pd^0^ catalytic cycle involving migratory insertion followed by β-bromide elimination^75,76^ or a Pd^II^/Pd^IV^ pathway proceeding through oxidative addition and subsequent reductive elimination furnishes the desired product (**25**).^77^ In either scenario, the silver salt can be proposed to activate the alkyne and sequester bromide ions that might otherwise poison the catalyst. The catalytic system, comprised of Pd­(OAc)_2_, ethylenediamine-derived ligand (**L10** and **L11**, Scheme 13), Ag_2_O, and LiHFIP, provided access to a wide range of alkynylated α-quaternary acids (**25a**–**25b**) in synthetically useful yields.

**Scheme 13:**
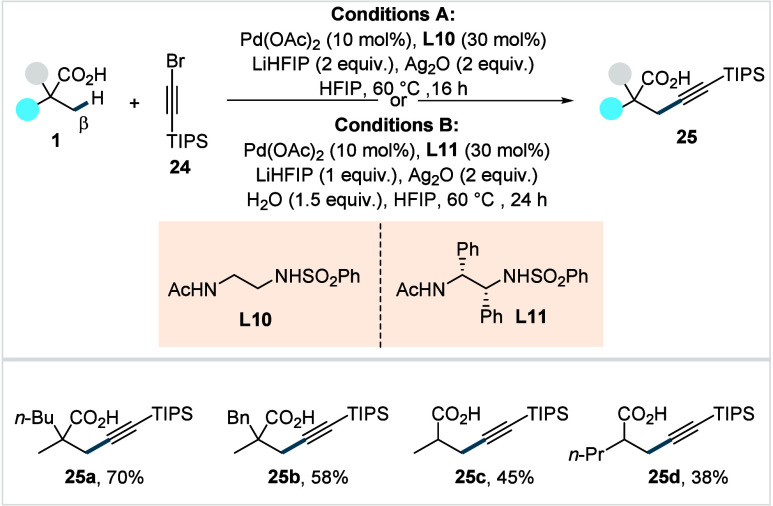
van Gemmeren et al. (**2020**): Direct β-C­(sp^3^)–H Alkynylation of Aliphatic Acids

The methodology was further extended to α-nonquaternary acids under slightly modified reaction conditions (Conditions B, Scheme 13), albeit affording the respective alkynated acids in moderate yields (**25c**–**25d**). Mechanistic studies revealed that the C–H activation is the rate-determining step of the reaction.

In 2021, the Yu’s group developed a method for the synthesis of cyclic anhydrides via β-C­(sp^3^)–H carbonylation of free carboxylic acids.^78^ The reaction employed a bidentate thioether-based ligand (**L8**, Scheme 14) in combination with Mo­(CO)_6_ as a convenient solid CO source. The superior reactivity of **L8** compared to other ligand classes was attributed to its neutral phenylthio moiety, which stabilizes Pd^0^ and facilitates the terminal oxidation step. A broad spectrum of aliphatic acids, including the challenging α-nonquaternary acids was compatible with this protocol, affording the respective anhydrides in moderate to good yields (**27a**–**27d**). For practical handling, the initially formed cyclic anhydrides were hydrolyzed and benzylated. The utility of the protocol was further highlighted by the selective generation of diverse β-functionalized products.

**Scheme 14:**
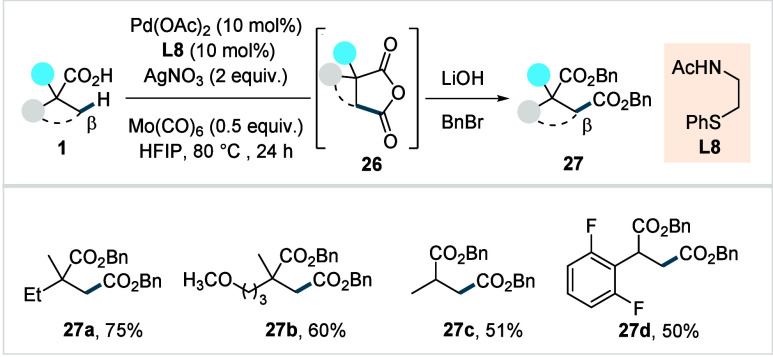
Yu et al. (**2021**): Direct β-C­(sp^3^)–H Carbonylation of Aliphatic Carboxylic Acids

More recently, the same group employed amino acid- and thioether-derived ligands, which selectively promoted β-C­(sp^3^)–H methyl olefination and arylation in the presence of methylene C­(sp^3^)–H bonds in cyclic carboxylic acids, enabling the synthesis of a diverse range of spiro carbocycles, including pharmaceutically relevant spiro-3,4-dihydrocoumarins and spirocyclic ketones.^79^

#### 2.1.2 γ-Methyl-C­(sp^3^)–C Bond Forming Reactions

While carboxylic acid directed β-C­(sp^3^)–H activation proceeds via formation of a kinetically favored five-membered palladacycle, distal γ-C­(sp^3^)–H activation requires the formation of a thermodynamically less stable six-membered palladacycle and is therefore more challenging to achieve. Advances in this direction have primarily been achieved with substrates lacking competing β-C­(sp^3^)–H bonds, thereby avoiding the intrinsic preference for β-C­(sp^3^)–H activation. Nevertheless, identifying suitable combinations of catalyst, reagent, base, and silver salt, has been crucial for the development of efficient methods. Despite the constraints to the substrate structure, the resulting protocols remain synthetically valuable, enabling the direct γ-C­(sp^3^)–H functionalization of aliphatic carboxylic acids, including the readily available 3,3-dimethylbutanoic acid and the naturally occurring 3-methylbutanoic acid.

In 2019, Maiti et al. reported the first example of γ-C­(sp^3^)–H arylation of aliphatic acids using a Pd­(OAc)_2_ and *N*-acetylglycine as catalytic system (**L2**, Scheme 15).^80^ Mechanistically, the reaction is proposed to proceed via a Pd^II^/Pd^IV^ catalytic cycle.

**Scheme 15:**
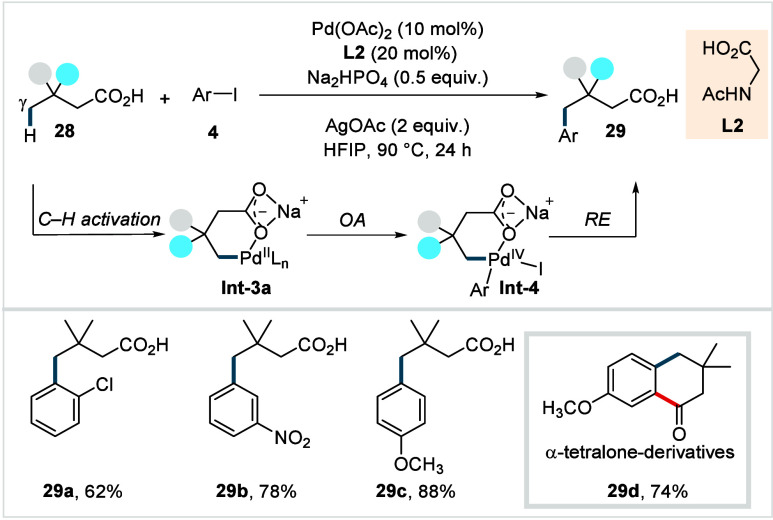
Maiti et al. (**2019**): Direct γ-C­(sp^3^)–H Arylation of Aliphatic Carboxylic Acids

The rate-determining γ-C­(sp^3^)–H activation step leads to the formation of a six-membered palladacycle **Int-3a**. A subsequent oxidative addition of the aryl iodide generates a Pd^IV^ intermediate **Int-4**, from which reductive elimination furnishes the desired product. Using the optimized conditions, 3,3-dimethyl butanoic acid was successfully arylated with diverse aryl iodides (**4**), including halide substituted (**29a**), electron-withdrawing (**29b**), and electron-donating groups (**29c**), delivering the corresponding products in good to excellent yields. The protocol was also compatible with complex molecule-derived aryl iodides. Notably, exclusive monoselectivity was observed across all examples. The authors further demonstrated iterative γ-arylation to generate diarylated acid architectures and showcased the synthetic utility of the products by converting the monoarylated acids into a library of α-tetralone derivatives in two steps (**29d**). Nevertheless, the scope of the aliphatic acids remained limited, and the even more challenging β-nonquaternary acids showed poor reactivity using this protocol.

Shi and co-workers have also detailed a γ-C­(sp^3^)–H arylation strategy for *N*-protected *tert*-leucine and its peptide derivatives.^81^ The study revealed that *N*-acetyl *tert*-leucine (**L12**, Scheme 16) as the ligand and Ag_3_PO_4_ as an additive were crucial to promote the desired reactivity. Ag_3_PO_4_ was proposed to play a dual role, acting as a halide scavenger and as a heteronuclear active species to facilitate C–H bond cleavage.

**Scheme 16:**
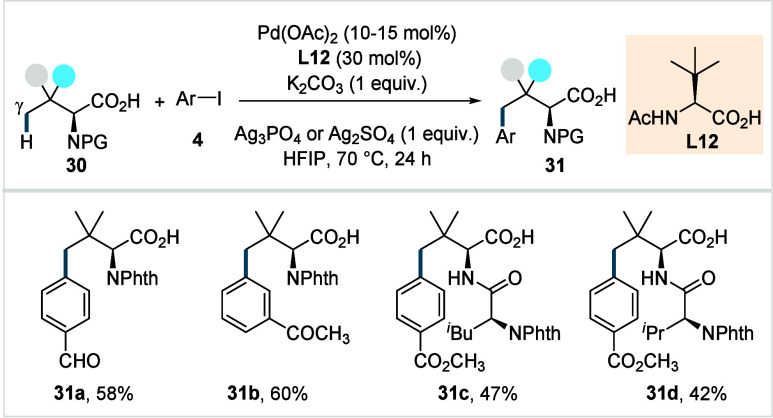
Shi et al. (**2020**): Direct γ-C­(sp^3^)–H Arylation of Aliphatic Carboxylic Acids

Various electronically substituted aryl iodides were coupled with *N*-phthaloyl protected *tert*-leucine, affording the corresponding arylated products in good yields (**31a**–**31b**), with minor quantities of diarylated products in some cases. The methodology was further extended to the late-stage γ-C­(sp^3^)–H arylation of di- and tripeptides, providing the functionalized peptides, albeit with moderate yields (**31c**–**31d**). Interestingly, the weakly coordinating carboxylate outcompeted the strongly coordinating bidentate directing group on the peptide backbone, enabling selective arylation of γ-C­(sp^3^)–H bonds of the *tert*-leucine moiety.

Given the challenges associated with the generation of γ-heteroarylated aliphatic acids via traditional carbonyl reactivity, the direct introduction of heteroaryl groups via a γ-C­(sp^3^)–H activation represents an attractive alternative. Yu et al. in their previously described β-C­(sp^3^)–H heteroarylation study employing a bidentate pyridine-pyridone ligand (cf. Scheme 6), also extended their methodology to the heteroarylation of γ-C­(sp^3^)–H bonds (Scheme 17).^64^

**Scheme 17:**
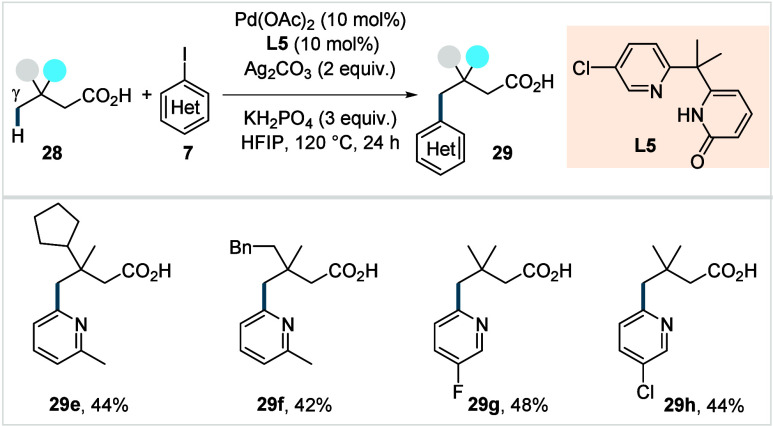
Yu et al. (**2022**): Direct γ-C­(sp^3^)–H Heteroarylation of Aliphatic Carboxylic Acids

Under the optimized conditions, a variety of heteroaryl iodides (**7**) was tolerated, affording the corresponding γ-heteroarylated acids in moderate yields (**29e**–**29h**). Comparable reactivity was observed for 2-iodopyridines irrespective of their substitution at the 6-position. However, the catalytic system was found to be ineffective for the γ-C­(sp^3^)–H heteroarylation of β-nonquaternary acids.

In 2020, van Gemmeren et al. disclosed a protocol for the γ-C­(sp^3^)–H olefination of aliphatic carboxylic acids.^82^ The reaction proceeds via a Pd^II^/Pd^0^ cycle to give γ-olefinated acid (**Int-6a**, Scheme 18), which undergoes a subsequent intramolecular Michael addition to afford γ-lactone **32**. Two types of six-membered chelating ligands Ac-β-Ala-OH (**L3**, Conditions A, Scheme 18) and *N*-acetyl anthranilic acid were found to be equally efficient for this transformation.

**Scheme 18:**
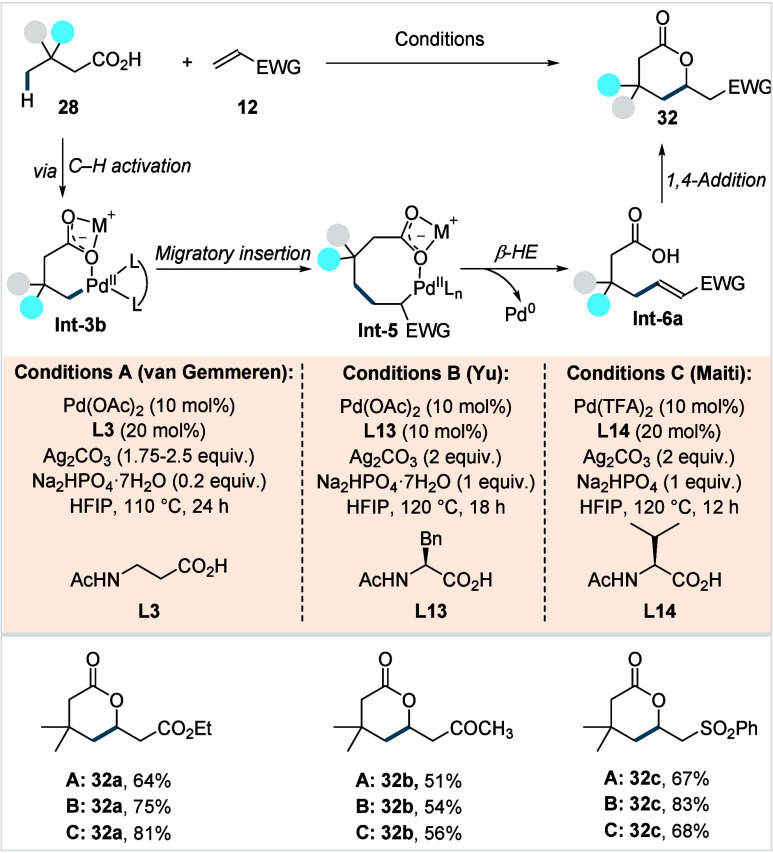
van Gemmeren, Yu, and Maiti et al. (**2020**): Direct γ-C­(sp^3^)–H Olefination of Aliphatic Carboxylic Acids

Ac-β-Ala-OH was chosen for scope investigation due to its broader availability. A variety of aliphatic acids bearing β-quaternary centers and activated olefins (**12**) were coupled to generate γ-lactones (**32a**–**32c**) in moderate to good yields. Contemporaneously, the groups of Yu and Maiti developed similar methodologies for the construction of γ-lactones.^83,84^ In both cases, five-membered chelating ligands (**L13**, Conditions B and **L14**, Conditions C; Scheme 18) with suitable substitutions on the backbone proved effective. Both protocols exhibited a broad functional group tolerance, enabling access to a diverse library of γ-lactones in good yields. The group of Maiti furthermore used *N*-alkyl and *N*-aryl substituted maleimides to form fused seven-membered δ-lactones. However, for all three protocols the scope remained limited to β-quaternary acids and activated olefins as reaction partners. The olefination reaction is proposed to operate via a Pd^II^/Pd^0^ catalytic cycle. The reaction is initiated by a γ-C­(sp^3^)–H activation, facilitated by the external ligand and the countercation of the base, to form a six-membered palladacycle **Int-3b**. Subsequent ligand exchange, olefin coordination, and migratory insertion (MI) generate an eight-membered cyclopalladated intermediate **Int-5**. From this intermediate, β-hydride elimination followed by decoordination affords **Int-6a**, which then undergoes intramolecular Michael addition to furnish the product. The resulting Pd^0^ species is subsequently reoxidized to Pd^II^ by Ag^I^.

In 2022, Maiti and co-workers reported a dual γ-C­(sp^3^)–H activation approach for the synthesis of γ-lactones through the coupling of simple carboxylic acids with allyl alcohols (**16**) or acetates.^85^ Employing *N*-acetyl *tert*-leucine as ligand (**L12**, Scheme 19), a range of β-quaternary acids and various primary secondary, and tertiary allyl alcohols, as well as allyl acetates were used to afford the corresponding γ-lactones (**33a**–**33d**) in useful yields.

**Scheme 19:**
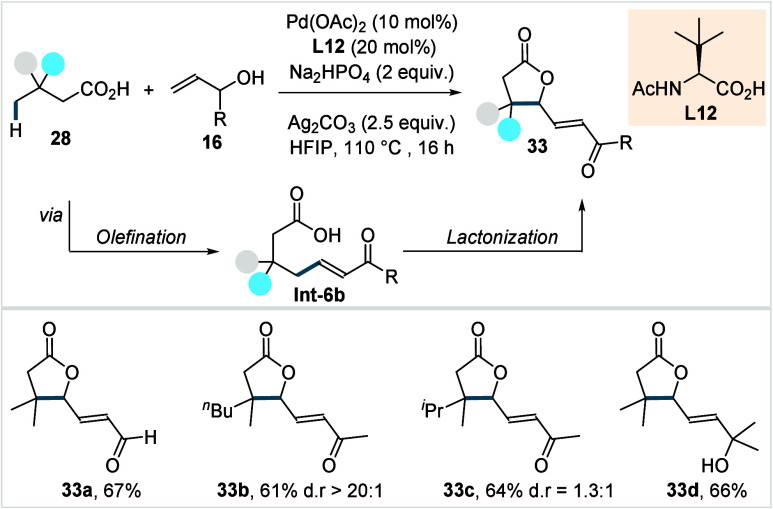
Maiti et al. (**2020**): Dual γ-C­(sp^3^)–H Activation of Aliphatic Carboxylic Acids with Allyl Alcohols

While electron-deficient aryl-substituted allyl alcohols were well-tolerated, their electron-rich counterparts were incompatible with the protocol. Preliminary mechanistic investigations suggested the involvement of a Pd^II^/Pd^0^ cycle, wherein the initially formed olefinated acid bearing a hydroxyl group would undergo oxidation to generate **Int-6b**. From this stage, a sequence of allylic C­(sp^3^)–H activation and C–O reductive elimination was proposed to deliver the final product.

The van Gemmeren group, in their previously discussed study on β-C­(sp^3^)–H alkynylation of aliphatic acids (Scheme 13)^74^ demonstrated that the method could also be adaptable for γ-C­(sp^3^)–H alkynylation. Using the bidentate ligand **L11** (Scheme 20) and introducing a slight adjustment to the standard conditions, the protocol delivered a range of γ-alkynylated acids (**34a**–**34d**). However, the yields were moderate and higher Pd-catalyst loadings were required to enable this transformation.

**Scheme 20:**
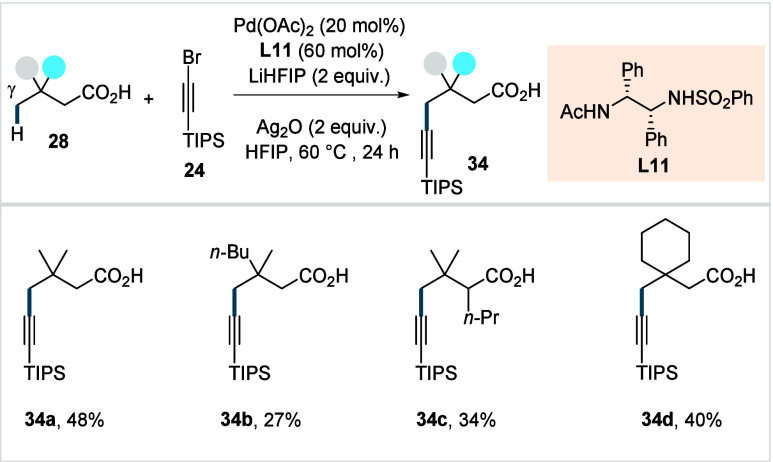
van Gemmeren et al. (2020): Direct γ-C­(sp^3^)–H Alkynylation of Aliphatic Carboxylic Acids

#### 2.1.3 Methylene-C­(sp^3^)–C Bond Forming Reactions

It should be noted that the activation of methylene C–H bonds is generally more challenging than the analogous activation of methyl C–H bonds, primarily due to increased steric hindrance and reduced C–H acidity, which increase the C–H activation barrier and destabilize the resulting palladacycle. In addition, the resulting C–H-activated palladacycles bearing secondary alkyl–Pd bonds are further destabilized, by their propensity to undergo β-hydride elimination. This process is facilitated by adjacent α- and γ-C–H bonds and becomes particularly prominent when subsequent steps (e.g., transmetalation or oxidative addition, RE) are slow. In the case of cyclopropane and cyclobutane carboxylic acids and related motifs, the C–H activation step is facilitated by ring strain, increased π character of the C–C bonds, and reduced activation entropy arising from their rigid and conformationally biased structures, which enable prealignment of the carboxylic acid moiety with the C–H bond to be activated.^86–89^ Additionally, the metallacycles derived from such small rings are less prone to decomposition via β-hydride elimination, as their rigid conformations prevent the system from adopting the *syn* periplanar geometry required for this process. Conversely, the free rotation about C–C bonds and the conformational flexibility of acyclic linear carboxylic acids impose a substantial entropic penalty for accessing the C–H activation transition state. Additionally, this flexibility facilitates adoption of the *syn* periplanar geometry required for β-hydride elimination, rendering this side reaction more facile in acyclic systems. Consequently, C­(sp^3^)–H functionalization of acyclic linear carboxylic acids has historically represented a formidable challenge. It should be noted, however, that in suitably designed catalytic manifolds, β-H-elimination can deliberately be harnessed to enable subsequent functionalizations (*vide infra*).

In 2018, Yu et al. disclosed the first enantioselective C­(sp^3^)–H arylation of cyclopropane carboxylic acids.^68^ This transformation generates two chiral centers through a desymmetrization process, in which two previously identical methylene groups are differentiated into a methylene and a methine group. The authors found that for this transformation an ethylenediamine-derived *N*,*N*-donor ligand (**L4**, Conditions A, Scheme 21) with a benzyl substitution was the most effective in terms of yield and stereoinduction. A variety of aryl iodides (**4**), including those bearing electron-withdrawing and electron-donating groups, was coupled with cyclopropane carboxylic acid derivatives to afford the corresponding products in high yields and excellent enantioselectivity (**36a**–**36b**).

**Scheme 21:**
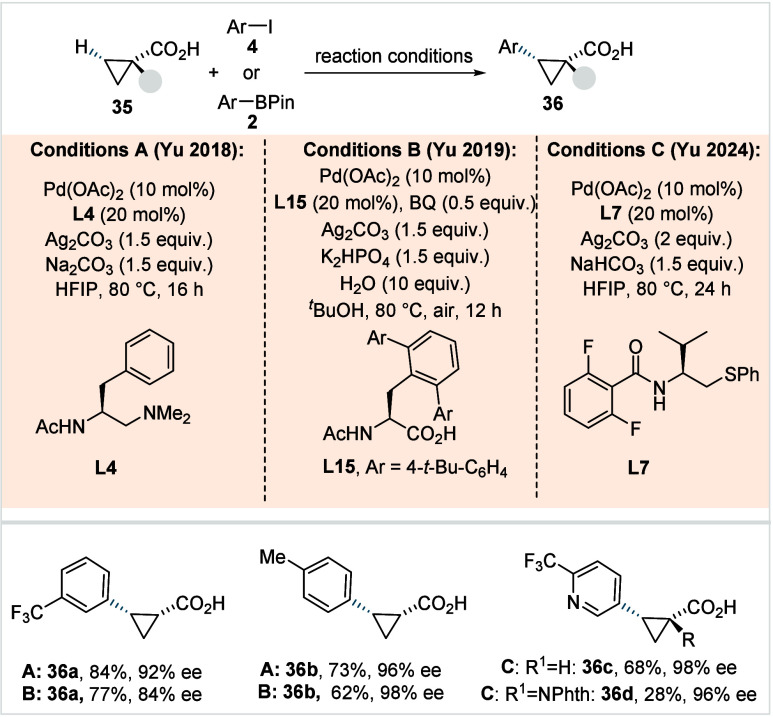
Yu et al. (**2018, 2019, 2024**): Enantioselective β-Methylene-C­(sp^3^)–H Arylation of Cyclopropane Carboxylic Acids

In the following year, the same group employed aryl pinacol boronates (**2**) as arylating agents to achieve enantioselective arylation of cyclopropane carboxylic acids via a Pd^II^/Pd^0^ cycle.^90^ The reaction employed a bidentate amino acid-derived ligand with a very bulky substituent on the backbone (**L15**, Conditions B, Scheme 21) and was performed in *tert*-butanol/H_2_O as the solvent. Various aryl pinacol boronates were successfully coupled with cyclopropane carboxylic acid derivatives. By switching to *N*,*N*-donor ligands with a suitably modified backbone, the method was extended to cyclobutane carboxylic acid derivatives, providing the respective arylated acids in useful yields and enantiomeric ratios typically above 95:5. Moreover, the methodology was successfully adapted for analogous enantioselective vinylation. However, none of these protocols were compatible with α-amino-substituted cyclopropane carboxylic acids and heterocyclic coupling partners. To overcome this, the authors recently developed a protocol employing a thioether-derived ligand (**L7**, Conditions C, Scheme 21) with an isopropyl substituent on the backbone and 2,6-difluoro benzoyl moiety as internal base.^69^ This protocol enabled the coupling of a wide range of aryl and heteroaryl iodides with both cyclopropane and α-amino-substituted cyclopropane carboxylic acids, affording the respective products (**36c**–**36d**) in moderate to good yields and excellent enantioselectivities.

More recently, the same research group described the use of chiral sulfonamide-derived ligands to achieve enantioselective β-C­(sp^3^)–H arylation of fluoroalkyl-substituted cyclopropane and cyclobutane carboxylic acids, providing access to a broad range of small-ring frameworks featuring fluoroalkyl-substituted quaternary centers.^91^

In their β-C­(sp^3^)–H alkynylation of aliphatic acids (Scheme 13),^74^ van Gemmeren et al. employed a chiral, enantiopure ethylene diamine-derived ligand, achieving the enantioselective alkynylation of cyclopropane carboxylic acid to furnish the product in a modest 35% yield and 39% ee.

In their previously discussed study (*cf*. Scheme 5),^63^ the Yu lab also demonstrated that cyclopropane and cyclobutane carboxylic acids could undergo β-C­(sp^3^)–H arylation with DNA-tethered (het)­aryl iodides to afford their respective DNA-labeled products in moderate yields (**36e**–**36f**, **38a**, Scheme 22).

**Scheme 22:**
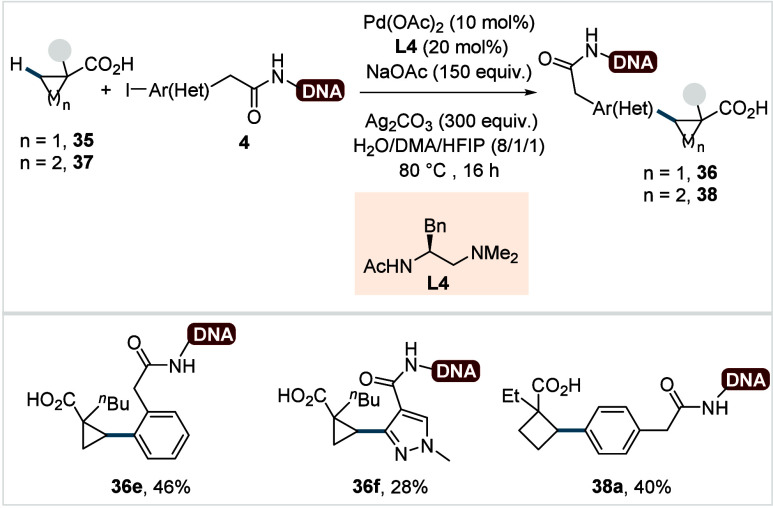
Yu et al. (**2020**): β-Methylene-C­(sp^3^)–H Arylation of Cycloalkane Carboxylic Acids DNA-Tethered (Het)­aryl Iodides

In 2022, Yu and co-workers reported a Pd­(II)-catalyzed β-methylene-C­(sp^3^)–H arylation of simple aliphatic acids.^92^ The authors observed that the ligand’s bite-angle was crucial for the desired reactivity. While a six-membered quinoline-pyridone-based bidentate ligand showed moderate reactivity, a substantial improvement in yield was achieved upon removal of the methylene spacer connecting the quinoline and pyridone units (**L16**, Scheme 23).

**Scheme 23:**
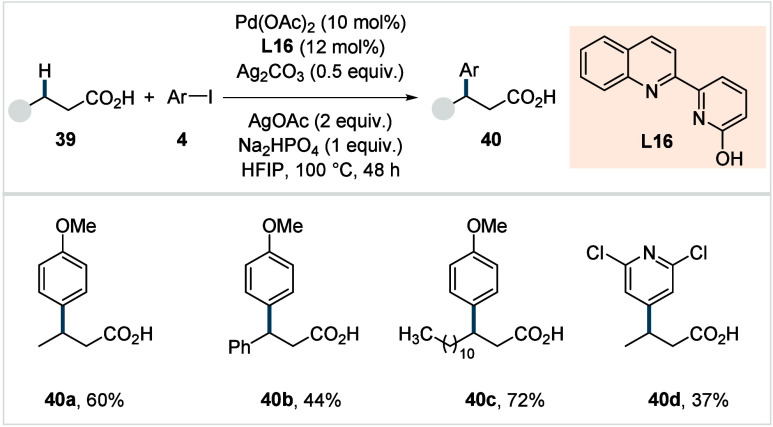
Yu et al. (**2022**): β-Methylene-C­(sp^3^)–H Arylation of Aliphatic Carboxylic Acids

The use of silver carbonate in combination with silver acetate was beneficial for the reaction outcome. A diverse set of arylated aliphatic acids, including linear acids (**40a**), phenylpropionic acid derivaties (**40b**), and fatty acids (**40c**) was obtained in moderate to good yields. The reaction was sensitive to the presence of sterically hindered substituents at the β-position of acid substrates, which resulted in a slight reduction of the yield. Various aryl iodides, including heteroaryl iodides were compatible with this protocol (**40d**); although it should be noted that electron-neutral or electron-rich aryl iodides outperformed electron-poor ones. While a plausible reaction mechanism was not explicitly proposed in this study, based on mechanistic studies on related systems it seems reasonable that after C–H activation the reaction either proceeds though a desaturation/Heck-type reaction pathway or via oxidative addition followed by reductive elimination.

In 2023, Yu et al. reported a formal [2 + 2] cycloaddition strategy for the regio-controllable synthesis of benzocyclobutane (BCB) scaffolds via double C–H functionalization of adjacent β- and γ-methylene C­(sp^3^)–H bonds.^93^ The method utilizes pyridone-amide-based ligands (**L17** and **L18**, Scheme 24) and dihaloarenes (**43**) as coupling partners.

**Scheme 24:**
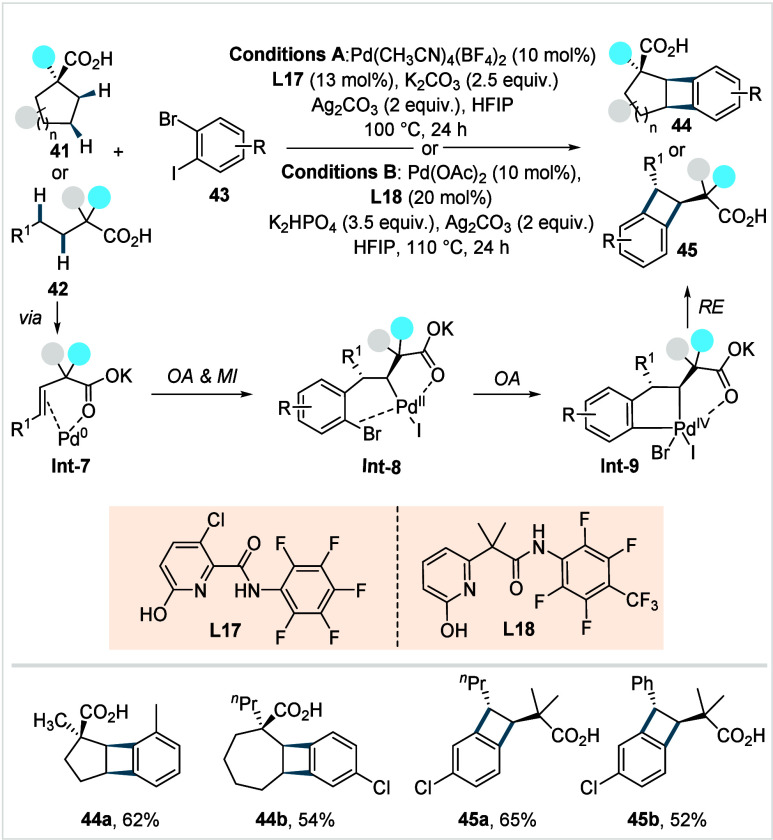
Yu et al. (**2023**): [2 + 2] Annulation of Two Adjacent Metheylene C­(sp^3^)–H Bonds

Mechanistic studies supported a Pd^II^/Pd^0^/Pd^II^/Pd^IV^ catalytic cycle, wherein ligand-enabled β,γ-desaturation generates **Int-7** and gives Pd^0^. The Pd^0^ species then undergoes selective oxidative addition into the aryl–iodine bond. A subsequent carboxylic acid-directed 1,2-migratory insertion delivers the γ-aryl intermediate **Int-8**. From this stage, intramolecular oxidative addition into the aryl–bromine bond forms **Int-9**, and a subsequent reductive elimination furnishes the BCB products (**44** or **45**). Interestingly, while the ligand was found to be essential for the β,γ-dehydrogenation step, it exerted no influence on the subsequent steps of the catalytic cycle. A wide range of cyclic aliphatic acids, including five-membered and seven-membered rings, was compatible, affording *cis*-BCB products as single isomers (**44a**–**44b**), whereas 11-or higher-membered ring containing cyclic acids delivered *trans*-BCB products. Under slightly modified conditions (Conditions B, Scheme 24), a diverse range of acyclic acids afforded the respective *trans*-BCB products in moderate to good yields (**45a**–**45b**). The observed diastereoselectivity of the BCB products was attributed to the dehydrogenation step, with small cyclic acids forming *cis*-alkenes and acyclic acids favoring *trans*-alkene. The protocol also displayed a broad functional group tolerance across a range of haloarenes. Recently, the authors extended this method to the γ,δ-methylene C­(sp^3^)–H coupling of aliphatic acids lacking β-C–H bonds with dihaloarenes, enabled by bidentate carboxyl-pyridone ligand.^94^

This year, the same group disclosed a protocol that, using a pyridine-pyridone ligand, enabled a formal Diels–Alder reaction between cyclic carboxylic acids and dienophiles, resulting in bridged bicyclic motifs with exclusive endo selectivity. In this protocol, dienes were generated in situ from saturated cyclic acids through a sequence of β,γ-dehydrogenation, decarboxylation, and subsequent regioselective β-hydride elimination, and could then be intercepted with a variety of dienophiles.^95^

In 2023, Yu et al. disclosed a highly regioselective transannular γ-C­(sp^3^)–H arylation of cycloalkane carboxylic acids, ranging from cyclobutanes to cyclooctanes.^96^ Ligand selection was tailored to the ring size to achieve optimal reactivity. Quinuclidine-pyridone ligands (**L19** and **L20**, Scheme 25) were used for γ-arylation of 5- to 8-membered cycloalkane rings, affording the respective products in moderate to excellent yields (**46a**–**46b**).

**Scheme 25:**
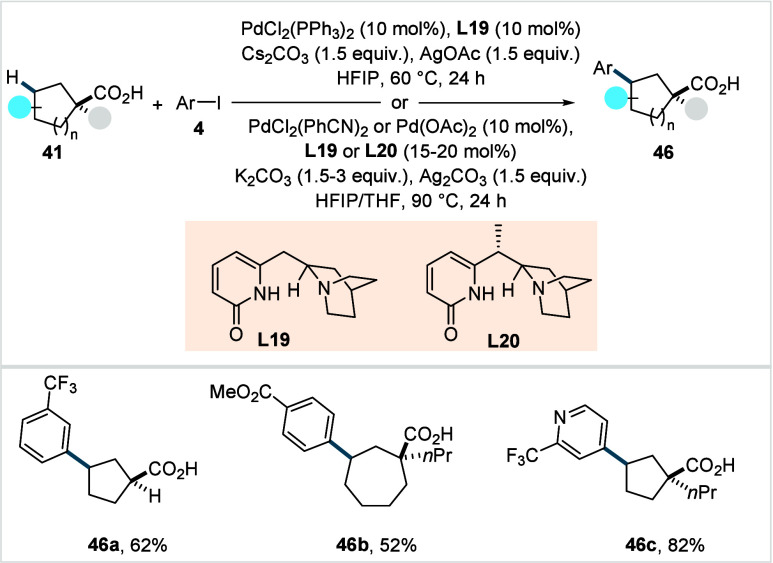
Yu et al. (**2023**): Transannular γ-C­(sp^3^)–H Arylation of Cycloalkane Carboxylic Acids

X-ray analysis confirmed *cis*-installation of the aryl substituent relative to the carboxylic acid directing group. A broad range of aryl iodides, including heteroaryl iodides, was well tolerated by this protocol (**46c**), albeit α-heteroatom-substituted cycloalkane acids and aryl iodides bearing acidic functionalities (e.g., −CO_2_H, NHTs, etc.) exhibited poor reactivity. The synthetic utility of the method was demonstrated by the preparation of various precursors for histone deacetylase inhibitors. Furthermore, the authors employed a combination of sulfonamide-pyridone-based bidentate ligand and a monodentate pyridone derivative to achieve exclusive γ-arylation of cyclobutane carboxylic acids via cross-dehydrogenative coupling with various arenes as coupling partners.

Recently, Lei and co-workers conducted a DFT investigation of the Yu’s γ-C­(sp^3^)–H (hetero)­arylation of cycloalkane carboxylic acids (Scheme 25).^97^ Their study suggested that the β-C­(sp^3^)–H bond is preferentially activated first, from here, a transient β,γ-unsaturated acid intermediate is formed through a ligand assisted pathway with concomitant formation of Pd^0^. Subsequently, a sequence of oxidative addition, carbopalladation delivering the aryl group to the γ-position and protodepalladation delivers the observed γ-arylated products.

In the following year, Yu et al. developed a protocol for the enantioselective methylene γ-C­(sp^3^)–H (hetero)­arylation of 5- to 8-membered cycloalkane carboxylic acids.^98^ Using a chiral Pd/oxazoline-pyridone catalyst (**L21**, Scheme 26), the method enabled formation of γ-tertiary stereocenters while concurrently desymmetrizing the α-quaternary center of the cycloalkane acids. A broad range of aryl and heteroaryl iodides, as well as cycloalkane carboxylic acids (**46d**–**46f**) was well-tolerated, furnishing the respective *cis*-arylated products in moderate to high enantioselectivities and good yields.

**Scheme 26:**
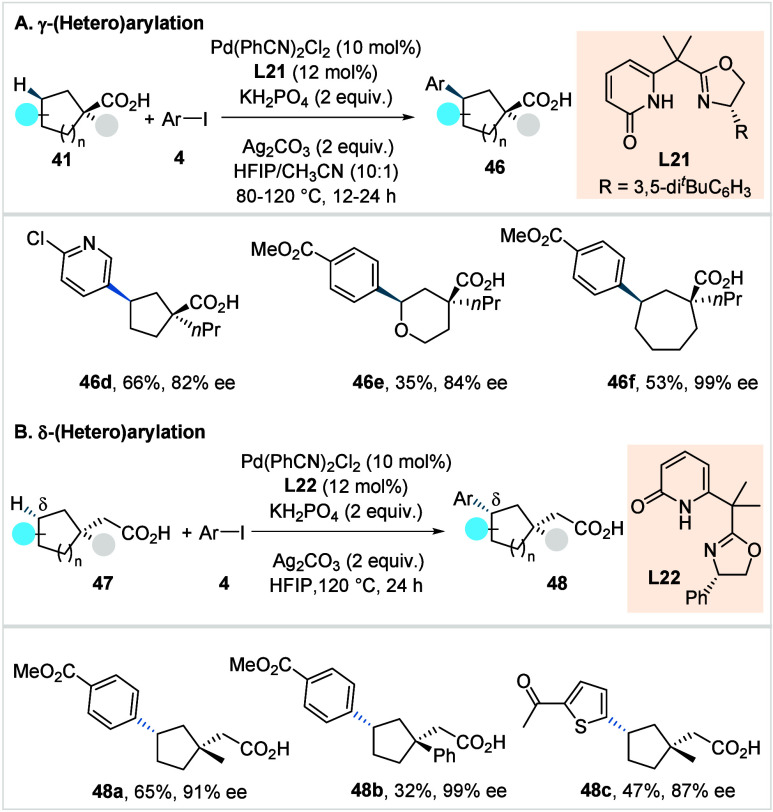
Yu et al. (**2024**): Enantioselective Methylene γ-C­(sp^3^)–H (Hetero)­arylation of Cycloalkane Carboxylic Acids

However, α-hydrogen bearing cycloalkane carboxylic acids resulted in diminished yields. To further expand the scope, a modified chiral Pd/oxazoline-pyridone catalyst (**L22**, Scheme 26) was introduced, enabling enantioselective δ-(hetero)­arylation for the simultaneous construction of β-quaternary and δ-chiral centers in carbocycles. The transformation featured a broad functional group compatibility, including challenging heterocycle-containing aryl iodides (**48a**–**48c**), although cycloalkane acids bearing β-hydrogens delivered δ-arylated acids in poor yield and modest enantioselectivity. Furthermore, the synthetic utility of the method was demonstrated through sequential γ-C–H arylation and heteroarylation to access carbocycles bearing three stereocenters.

Also in 2024, Yu and co-workers reported a protocol for the one-step synthesis of a complex chiral 9,10-dihydrophenanthrene scaffolds directly from α-substituted cyclohexane carboxylic acids and aryl iodides.^99^ With the aid of an enantiopure thioether-derived ligand (**L23**, Scheme 27), the catalytic system enabled the activation of two methylene C­(sp^3^)–H bonds and three C­(sp^2^)–H bonds, leading to the construction of four C–C bonds and three chiral centers in one pot. The catalytic cycle is proposed to commence with a stereoselective C­(sp^3^)–H activation, followed by dehydrogenation to generate β,γ-unsaturated intermediate **Int-10**. Subsequent, Heck-type carbopalladation introduces an aryl substituent at the γ-position, affording **Int-11**. Consecutive activations of *ortho*-C­(sp^2^)–H bonds and intermolecular arylations furnish **Int-12**, which undergoes a final C­(sp^2^)–H activation intramolecular C­(sp^2^)–C­(sp^3^) coupling to deliver **50**. A range of α-substituted cyclohexane carboxylic acids could be coupled with various aryl iodides, affording the respective products in decent yields and good enantioselectivities (**50a**–**50c**), with the diastereomeric ratio typically above 20:1. It was observed that electron-rich aryl iodides resulted in lower yields, while *ortho*- and/or *meta*-substituted iodoarenes were not compatible with this protocol.

**Scheme 27:**
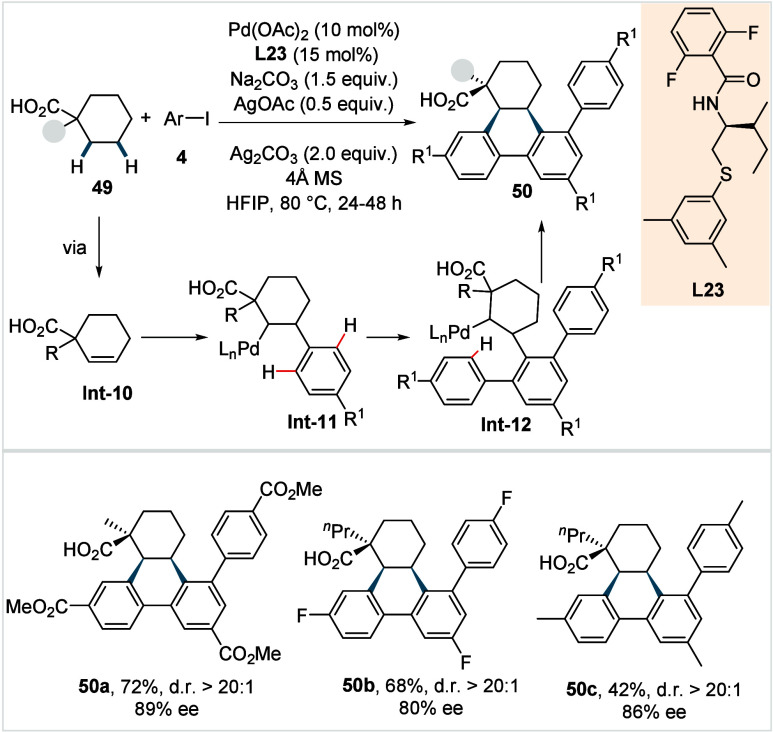
Yu et al. (**2024**): Enantioselective Cascade β,γ-Methylene C­(sp^3^)–H Diarylation of Cyclohexane Carboxylic Acids

### 2.2 Carbon–Oxygen Bond Forming Reactions

#### 2.2.1 β-Methyl-C­(sp^3^)–O Bond Forming Reactions

Carbon–oxygen bonds are ubiquitous in drugs, agrochemicals, and natural products. The direct oxygenation of unactivated C­(sp^3^)–H bonds via transition metal-catalysis offers an efficient yet challenging synthetic strategy. In 2019, van Gemmeren et al. disclosed the first Pd­(II)-catalyzed β-C­(sp^3^)–H activation/acetoxylation of free carboxylic acids.^100^ The reaction employed (diacetoxyiodo)­benzene (**51**) as the oxidant and was conducted in a mixture of Ac_2_O and HFIP.

The reaction was initially found to be efficient using preformed alkali carboxylate salts. It was observed that the reaction proceeded substantially worse with free carboxylic acids **1** alongside typical alkali metal salts for in situ deprotonation, presumably due the detrimental effect exerted by the conjugated acid formed after substrate deprotonation. The use of the sodium salt of HFIP as a “traceless” base restored the desired reactivity. Interestingly, external ligands did not have a beneficial effect on the reaction outcome. For various aliphatic acids, the corresponding acetoxylated acids were obtained in moderate to good yields (**52a**–**52c**, Scheme 28). The authors further expanded the protocol to enable analogous acyloxylations by modifying the oxidant and anhydride (**52d**). However, this protocol required an excess of the acid substrates and remained limited to α-quaternary acids.

**Scheme 28:**
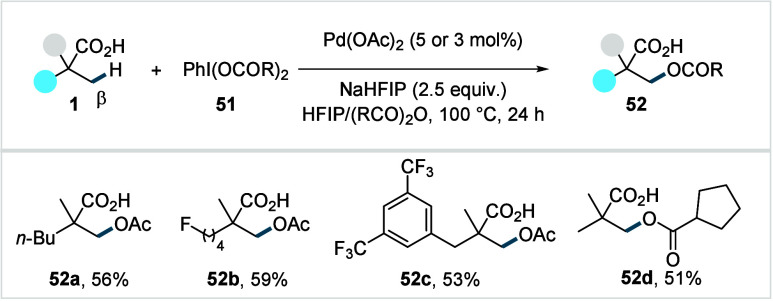
van Gemmeren et al. (**2019**): β-C­(sp^3^)–H Acetoxylation of Aliphatic Carboxylic Acids

In 2020, Yu et al. reported a protocol for the synthesis of strained β-lactones directly from aliphatic carboxylic acids via β-C­(sp^3^)–H activation.^101^ Using Pd­(CH_3_CN)_2_Cl_2_ as the precatalyst and TBHP as an inexpensive oxidant, the method was enabled by a six-membered chelating β-amino acid-derived ligand (**L24**, Scheme 29) featuring a methyl substituent on the backbone. The ligand was proposed to promote the C­(sp^3^)–O reductive elimination due to its increased bite angle compared to five-membered chelating ligands. The methodology provided a diverse array of β-lactones, including those bearing saturated heterocycle (**53a**) and ketone (**53b**) moieties, as well as examples for late-stage functionalization (**53c**) in good to excellent yields. While a few α-nonquaternary acid-derived lactones (**53d**) were obtained, the scope of aliphatic acids was mostly restricted to α-quaternary acids. The utility of the protocol was further demonstrated by a gram-scale synthesis of **53c** using a reduced catalyst loading Pd (1 mol %), as well as diverse nucleophilic ring opening reactions of the β-lactone products to access a library of β-functionalized acids.

**Scheme 29:**
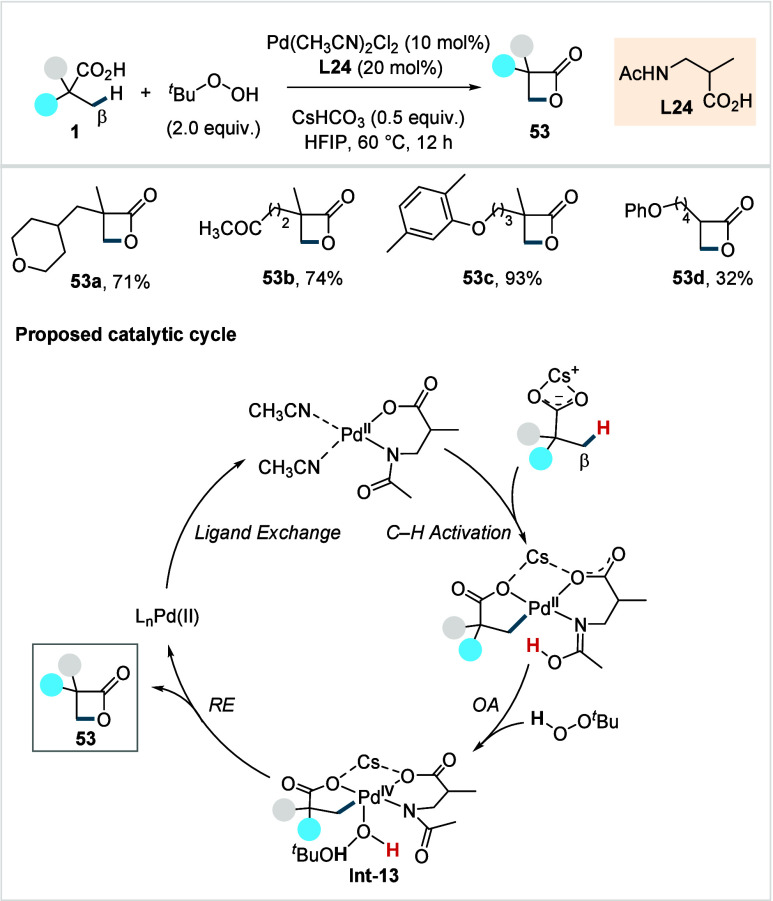
Yu et al. (**2020**): β-C­(sp^3^) Lactonization of Aliphatic Carboxylic Acids

The reaction was proposed to proceed via a Pd^II^/Pd^IV^ cycle, a hypothesis further supported by computational studies.^102^ The catalytic cycle begins with β-C­(sp^3^)–H activation, which is accelerated by both the alkali metal ion (Cs^+^) and bidentate ligand (**L24**). The resulting palladacycle undergoes a rate-limiting oxidative addition with TBHP to form **Int-13**. Interestingly, unlike the classical three-centered transition-state, the oxidative addition is proposed to involve the participation of the H atom of TBHP in a 1,2-hydrogen shift, which facilitates proton shuttling between the CMD group of **L24** and the Pd-coordinated hydroxyl oxygen of TBHP. From this stage, **Int-13** undergoes reductive elimination to yield **53** and the subsequent ligand exchange regenerates the catalyst. Importantly, both the external ligand **L24** and the OH-bearing oxidant were found to play essential roles in enabling the key steps of the catalytic cycle.

Building upon their previous lactonization study involving a Pd^II^/Pd^IV^ cycle, the Yu group later disclosed a more broadly applicable protocol for the β-C­(sp^3^)–H acetoxylation of aliphatic carboxylic acids, enabled by a cyclopentane-based β-amino acid ligand (**L6**, Scheme 30).^103^ This method uses inexpensive *tert*-butyl hydroperoxide (TBHP) as the sole oxidant and Ac_2_O as a promoter, affording the acetoxylated products under mild conditions (60 °C).

**Scheme 30:**
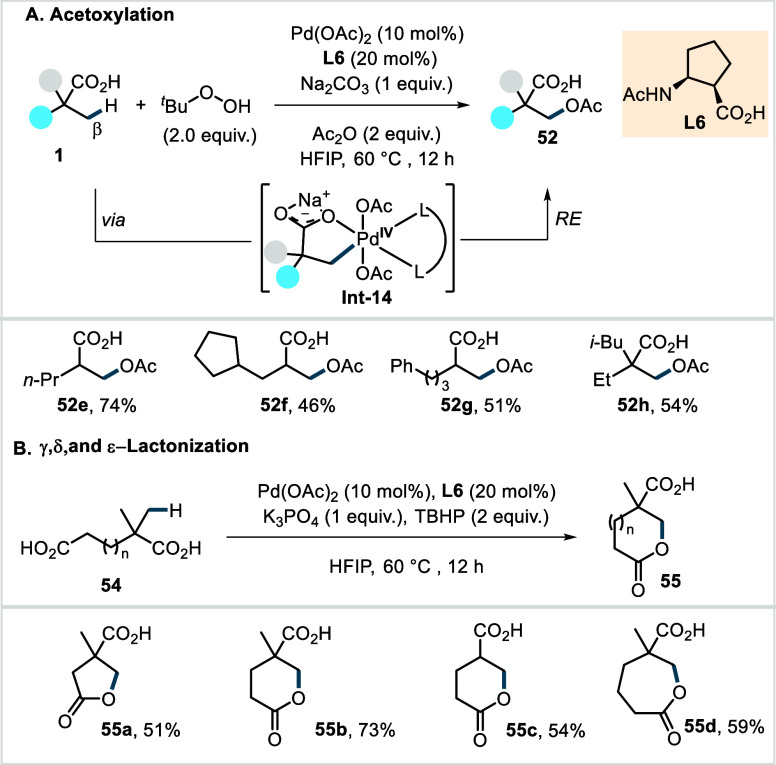
Yu et al. (**2020**): β-C­(sp^3^)–H Acetoxylation of Aliphatic Carboxylic Acids

The proposed mechanism involves a Pd^II^/Pd^IV^ cycle, initiated by ligand- accelerated β-C­(sp^3^)–H activation. The resulting palladacycle is then oxidized by TBHP to give a Pd^IV^ intermediate, which undergoes ligand exchange with Ac_2_O to generate **Int-14**. From this stage, reductive elimination affords **52** and regenerates the active catalyst. Notably, this protocol permitted the use of acid substrates as limiting reagents and exhibited good functional group tolerance. A wide variety of aliphatic acids, including the notoriously challenging α-nonquaternary acids, was successfully converted to the corresponding acetoxylated acids in moderate to good yields (**52e**–**52h**, Scheme 30A). Exclusive monoselectivity was observed for α-quaternary acids, which was attributed to a bidentate coordination of the monoacetoxylated products on the catalyst through the carboxylate group and the newly introduced OAc-group. This bidentate coordination prevents the remaining methyl groups from reaching the catalyst and thus suppresses difunctionalization. At the same time, a product inhibition of the reaction results, which was sometimes found to limit the conversions/yields. The protocol was further extended to intramolecular acyloxylations, wherein appropriately positioned second carboxylate groups within the substrates intercepted the catalytic cycle to furnish γ-(**55a**), δ-(**55b**–**55c**), and ε-lactones (**55d**) in good yields (Scheme 30B).

In 2025, van Gemmeren and co-workers reported a protocol for the direct β-C­(sp^3^)–H hydroxylation of aliphatic acids.^104^ The reaction employed TBHP as both the oxidant and hydroxyl source in the reaction, and was promoted by a pyridone-derived ligand (**L25**, Scheme 31).^105,106^ The reaction was proposed to proceed via a Pd^II/^Pd^IV^ catalytic cycle, initiated by C­(sp^3^)–H activation followed by oxidative addition with TBHP to generate **Int-15**. Subsequent intramolecular C­(sp^3^)–O reductive elimination furnishes **56**. The addition of DMF as an additive proved beneficial, likely due to its ability to stabilize high-valent Pd^IV^ intermediates by coordination and/or to facilitate the ligand exchange during catalyst regeneration.

**Scheme 31:**
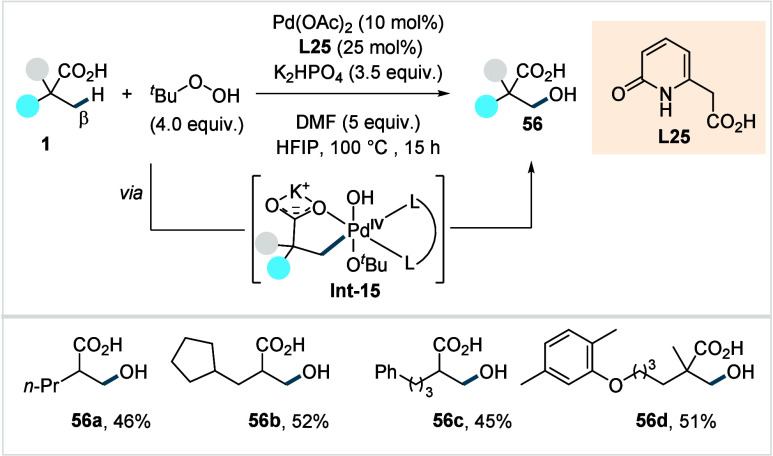
van Gemmeren et al. (**2025**): Direct β-C­(sp^3^)–H Hydroxylation of Aliphatic Acids

The method displayed good functional group tolerance, enabling the hydroxylation of diverse aliphatic acids, including the particularly challenging α-nonquaternary acids, as well as late-stage functionalization. Exclusive monoselectivity was observed for acids bearing α-dimethyl groups, a result attributed to the strong bidentate chelating ability of the monohydroxylated acids which prevents iterative hydroxylation. Furthermore, the moderate yields observed in the reaction were reasoned to arise from such competing coordination of the product to catalyst.

#### 2.2.2 γ-Methyl-C­(sp^3^)–O Bond Forming Reactions

In 1991, Kao and Sen reported the Pt-catalyzed lactonization of aliphatic carboxylic acids using potassium tetrachloroplatinate­(II) in D_2_O.^40^ For example, butanoic acid (**57**) furnished a mixture of γ-butyrolactone (**58**), resulting from γ-methyl C­(sp^3^)–H activation, and β-butyrolactone **59**, originating from β-methylene-C­(sp^3^)–H activation, along with the ring-opened γ-hydroxy acid (**60**) as a side product. Mechanistically, the carboxylate was proposed to coordinate to Pt^II^, followed by C­(sp^3^)–H activation to form a platinacycle. Subsequent reductive elimination gives the lactone product and generates a Pt^0^ species, which is reoxidized to the catalytically active Pt^II^ by the terminal oxidant (K_2_PtCl_6_) to close the catalytic cycle. Analysis of the product ratio revealed that γ-C­(sp^3^)–H bonds were preferentially activated, consistent with the formation of a less strained six-membered platinacycle. Interestingly, while significant reactivity was observed for β-, δ-, and ε-C­(sp^3^)–H bonds, the rates of oxidation for α-C–H bonds were very low and such processes were not even observed in the presence of β-C–H bonds. The protocol suffered from low turnover numbers and the need for stoichiometric platinum, which served as both catalyst and oxidant.

In 2001, Sames and co-workers reported an improved protocol for the γ-lactonization of amino acids in water, by employing CuCl_2_ as the oxidant.^107^ For l-valine (**61**), the γ-C­(sp^3^)–H lactonization product (**63**) was obtained as a mixture of stereoisomers in 27% overall yield (anti:syn = 3:1, Scheme 32b) after derivatization of the primary product **62**. However, the requirement for a high catalyst loading and the narrow substrate scope, due to the poor solubility of organic substrates in water, limit the practical application of this method. These limitations underscore the need for more efficient C­(sp^3^)–H oxidation strategies that operate with low catalyst loading and employ sustainable terminal oxidants, such as molecular oxygen or air, to enable wide adoption and large-scale applications of such methods.

**Scheme 32:**
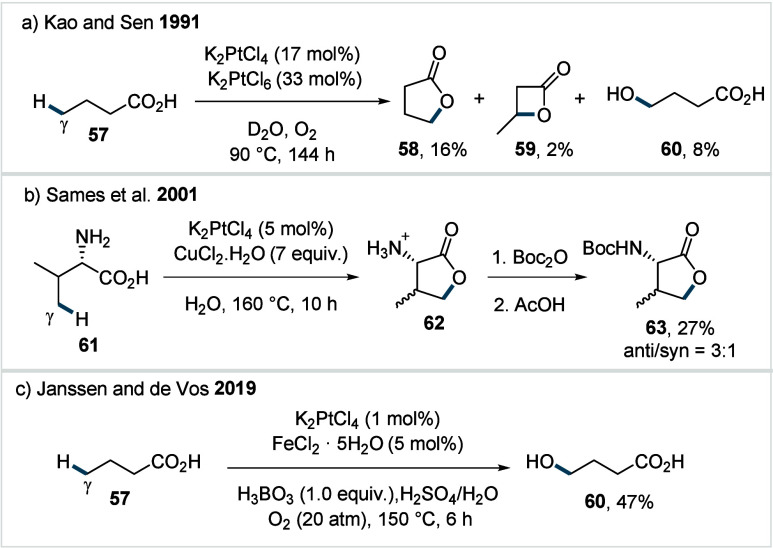
a) Lactonization of Aliphatic Carboxylic Acids Developed by Kao and Sen; b) Lactonization of Amino Acids by Sames et al.; c) Hydroxylation of Butanoic Acid by Janssen and de Vos

In 2019, Janssen and de Vos reported that terminal γ-C­(sp^3^)–H hydroxylation of butanoic acid (**57**) can be achieved using 1 mol % K_2_PtCl_4_ as the catalyst and oxygen as the terminal oxidant to afford γ-hydroxy butanoic acid (GHB, **60**) (Scheme 32c).^41^ The authors employed FeCl_2_ as a suitable redox mediator to overcome the kinetic barrier associated with the direct reoxidation of Pt^0^ by oxygen. Catalyst deactivation via reduction to Pt^0^ was effectively suppressed by either the addition of monodentate pyridine ligands or the modulation of the N_2_ partial pressure. Alternatively, the in situ protection of the hydroxyl group of GHB with boric acid was found to enhance the selectivity for the desired product, suppressing the formation of side products such as succinic acid and degradation products like acetic acid formed through overoxidation of GHB.

Although substantial progress has been made in transition metal-catalyzed benzylic C­(sp^3^)–H lactonization,^108–110^ the synthesis of γ-lactones via nonactivated C­(sp^3^)–H activation has remained a long-standing challenge. In 2024, Yu and co-workers disclosed a Pd­(II) catalyzed γ-C­(sp^3^)–H lactonization of aliphatic acids, enabled by five-membered chelating quinoline-pyridone ligand (**L16**, Scheme 33).^111^ The reaction proceeds via a Pd^II^/Pd^0^ cycle involving ligand-accelerated, irreversible γ-C­(sp^3^)–H activation to form a palladacycle. Next, a direct C­(sp^3^)–O RE delivers the γ-lactone **64** with concomitant generation of Pd^0^, which is subsequently reoxidized to Pd^II^. Although C­(sp^3^)–O reductive elimination is generally difficult from low-valent Pd^II^ species, it was rendered feasible by the optimal bite-angle of the ligand. The method exhibited a broad functional group tolerance, providing a range of γ-lactones, including an aliphatic side chain (**64a**), a benzyl group (**64b**), and spirocyclic scaffolds (**64c**), in moderate to good yield. However, the method remained ineffective for the functionalization of the β-nonquaternary acids (**64d**).

**Scheme 33:**
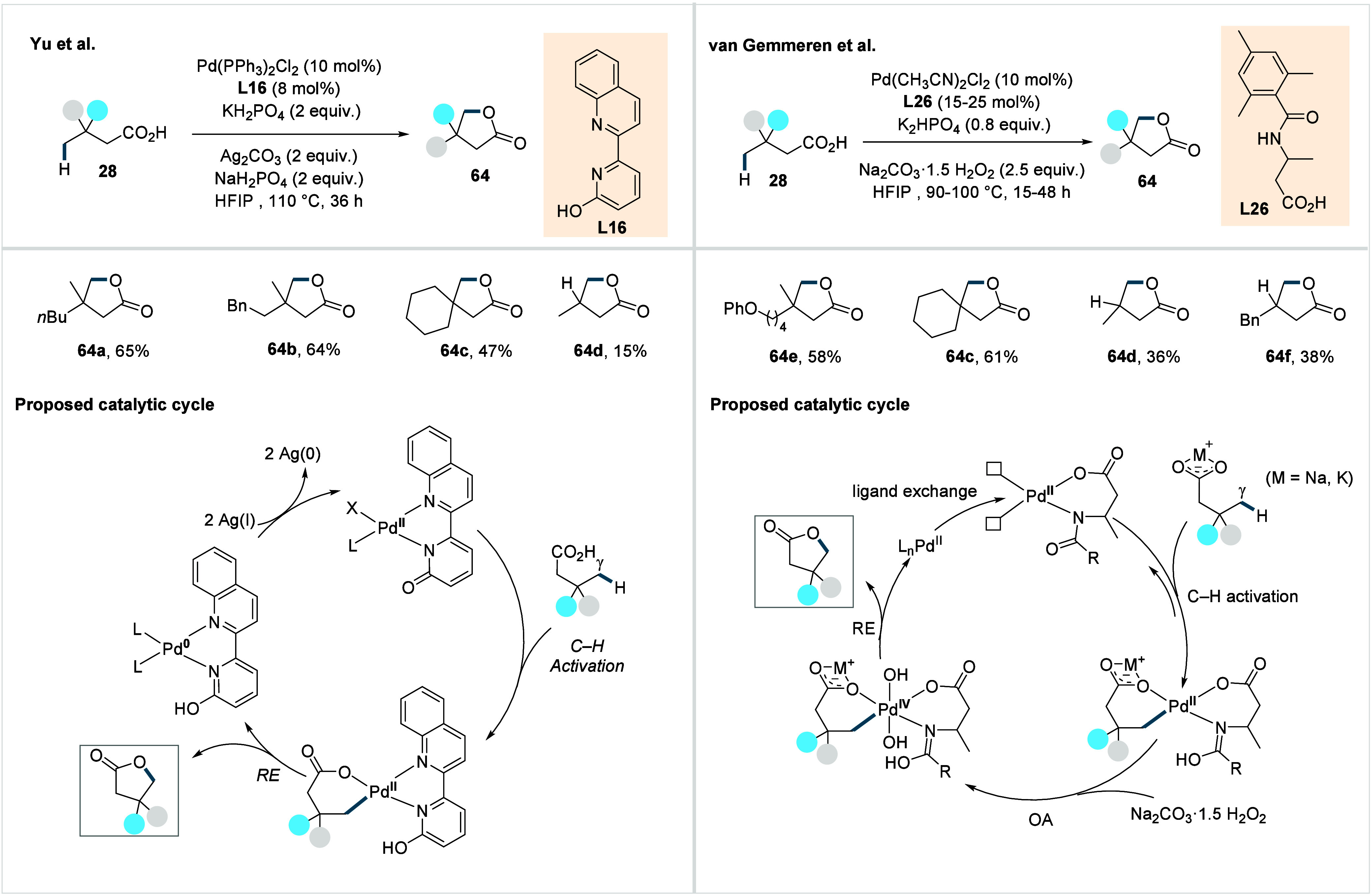
Yu et al. (**2024**) and van Gemmeren et al. (**2025**): Direct γ-C­(sp^3^)–H Lactonization of Aliphatic Carboxylic Acids

Contemporaneously, van Gemmeren et. al developed a protocol for the synthesis of γ-lactones based on a Pd^II^/Pd^IV^ cycle.^112^ The reaction employed a six-membered β-amino acid-derived ligand (**L26**, Scheme 33) and sodium percarbonate as a convenient oxidant. The incorporation of 2,4,6-trimethyl benzoyl group as CMD-promoting internal base and a methyl substituent on the ligand backbone proved crucial for the optimal reactivity. A range of γ-lactones with diverse substitution patterns was obtained in good yields.

Furthermore, the method was applicable to the functionalization of the challenging β-nonquaternary acids in moderate yields (**64d**, **64f**). Mechanistically, the transformation proceeds through ligand accelerated C–H activation to form a palladacycle, which is subsequently oxidized by sodium percarbonate to a high-valent Pd^IV^ intermediate. Subsequent C­(sp^3^)–O RE from the Pd^IV^ center affords the desired product and regenerates the active catalyst. A key advantage of the Pd^II^/Pd^IV^ manifold is the resistant of Pd^IV^ toward β-hydride elimination, an otherwise dominant decomposition pathway for palladacycles derived from the γ-C­(sp^3^)–H activation of β-nonquaternary acids, due to electronic (reduced nucleophilicity of Pd^IV^ relative to Pd^II^) and geometric constraints (absence of vacant coordination sites at coordinatively saturated octahedral Pd^IV^ complexes, and the energetically costly ligand dissociation required to create a suitably positioned vacant site for β-hydride elimination).^113–117^ This suppression of one of the undesired pathways is likely a decisive factor enabling the successful functionalization of β-nonquaternary acids.

#### 2.2.3 Methylene-C­(sp^3^)–O Bond Forming Reactions

In 2022, Yu and co-workers reported site-selective methylene C–H lactonization of dicarboxylic acids.^118,119^ The authors identified two distinct catalysts based on quinoline-pyridone-derived ligands for the site-selective β- and γ-methylene C­(sp^3^)–H activation of dicarboxylic acids, enabling three complementary modes of lactonization for the synthesis of γ- and δ-lactones (Scheme 34). The five-membered chelating quinoline-pyridone ligand **L16** (Scheme 34A) enabled β-C­(sp^3^)–H activation directed by one carboxyl group, while the other appropriately positioned carboxyl group, engaged in a subsequent γ- or δ-lactonization. The developed methodology provided access to a broad array of γ- (**67a**–**67b**) and δ-lactones (**68a**–**68b**) with diverse substitution patterns along the aliphatic chain, obtained in moderate to good yields. However, either a substituent in the β-position or an aromatic carboxyl group were required in order to form δ-lactones in synthetically useful yields. Additionally, the authors identified a six-membered chelating quinoline-pyridone-based ligand (**L27**, Scheme 34B), which enabled a selective γ-C­(sp^3^)–H activation even in the presence of competing β-C­(sp^3^)–H bonds. The method enabled the synthesis of a diverse range of γ-lactones, including α,β-unsubstituted (**70a**) and α-substituted (**70b**–**70c**) substrates as well as endocyclic γ-methylene C­(sp^3^)–H bonds (**70d**). Finally, the synthetic utility of the method was showcased through two total syntheses, enabling the construction of the natural products pedicellosine and myrotheciumone directly from dicarboxylic acids. Control experiments on the reaction mechanism revealed that α,β-unsaturated dicarboxylic acids, when subjected to the standard reaction conditions, afforded the desired products **67** or **68**, albeit in lower yields. The product **70** could not be obtained from the potential α,β-desaturated intermediate. This led the authors to conclude that the reaction most likely proceeds via a C–O reductive elimination rather than via desaturation, although this process may be operative as a secondary pathway for product formation.

**Scheme 34:**
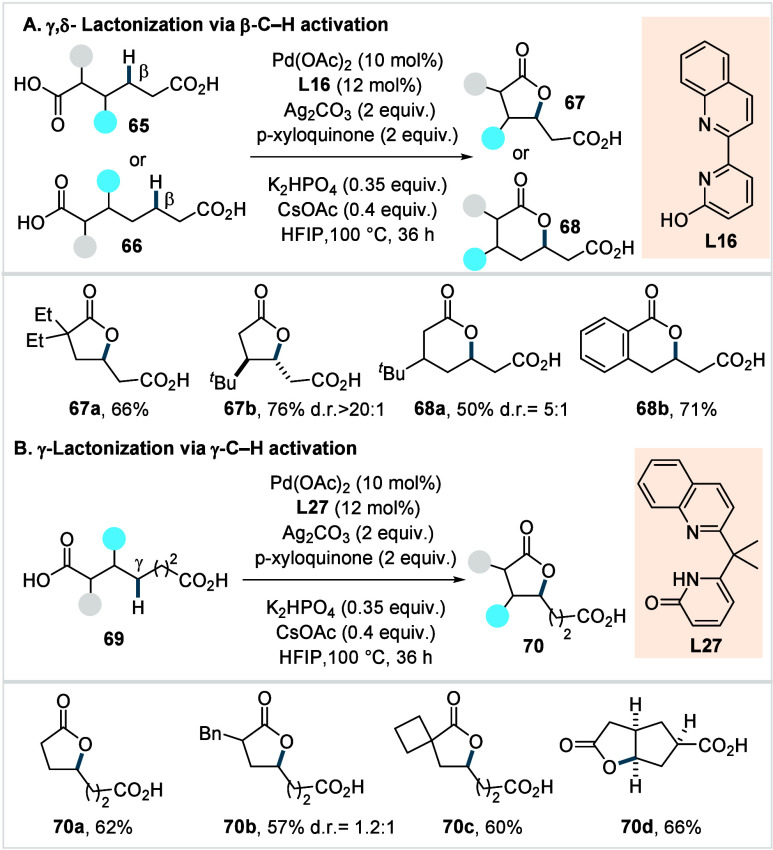
Yu et al. (2022): Methylene-C­(sp^3^)–H Lactonization of Dicarboxylic Acids

In 2023, Ge, Zhang, and Maiti et al. introduced a strategy for the construction of unsaturated bicyclic lactones via γ-methylene C­(sp^3^)–H activation.^120^ Using *N*-Ac-*tert*-leucine as bidentate ligand (**L12**, Scheme 35), the method enabled the selective functionalization of γ-methylene-C­(sp^3^)–H bonds even in the presence of intrinsically more reactive γ-methyl-C­(sp^3^)–H bonds. The preference for methylene over methyl activation was rationalized not by an unusually selectivity in the site of the C–H activation, but by a reversible C–H activation in both the methyl and methylene positions in conjunction with a prohibitive energy barrier for C–O reductive elimination from γ-methyl-derived palladacycle, whereas methylene-derived intermediate could undergo facile β-hydride elimination. Accordingly, the reaction is initiated by ligand-assisted γ-methylene C­(sp^3^)–H activation via a [5,6]-palladacyclic transition state (TS). The resulting methylene C­(sp^3^)–H-activated complex then undergoes γ,δ-dehydrogenation to form **Int-16**, outcompeting the energetically unfavorable C–O reductive elimination, as supported by DFT studies. Subsequently, stereoselective oxypalladation followed by β-hydride elimination furnishes **71**. The protocol delivered a diverse set of unsaturated lactones bearing substituents in the α-, β-, and γ-positions in excellent yields (**71a**–**71b**) and with high syn stereochemistry. The methodology also enabled efficient synthesis of fused bicyclic lactones across a broad range of ring sizes, spanning [5,5] to [15,5] systems (**71c**–**71d**). Furthermore, a broad library of bicyclic lactones was synthesized (**74a**–**74d**) by using acrylates and allyl alcohols (**73**) as coupling partners. In this case, the interception of the γ-C­(sp^3^)–H activated palladacycle by an olefin or allyl alcohol was proposed to from **Int-17**, which subsequently undergoes of allyl C­(sp^3^)–H activation and subsequent β-hydride elimination to produce **Int-18**. A final oxypalladation and β-hydride elimination sequence then delivers the desired lactones **74**. The synthetic utility of the protocol was underscored by its application to the formal synthesis of natural products and bioactive compounds.

**Scheme 35:**
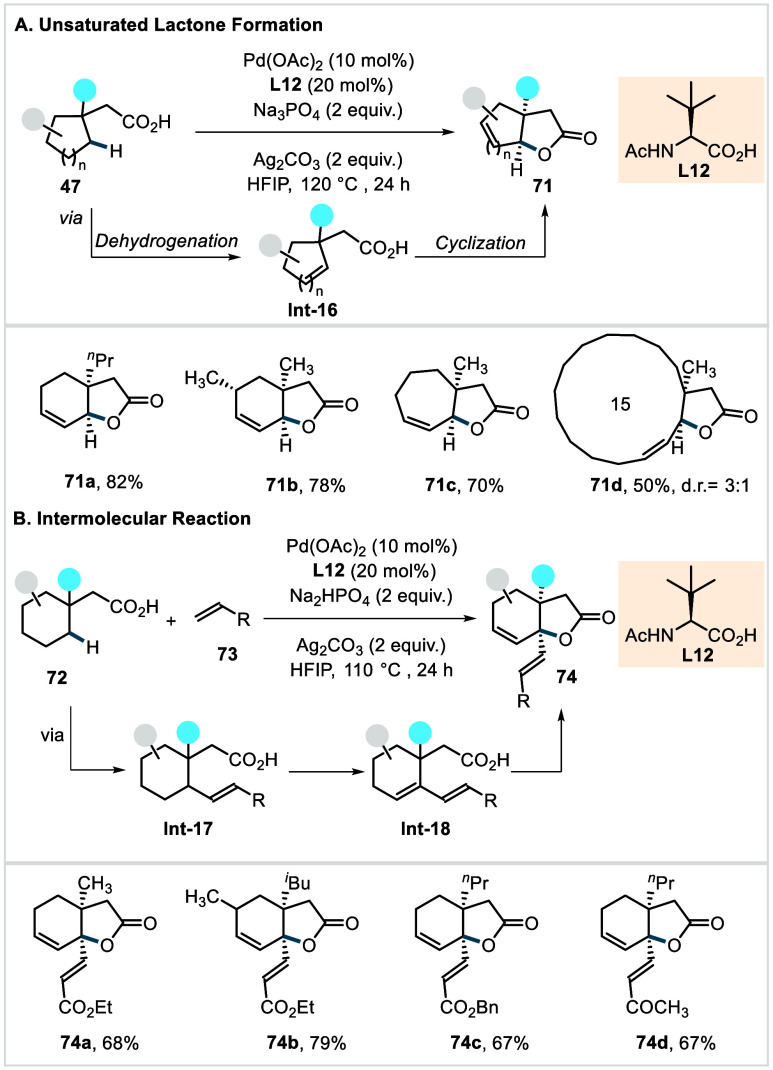
Maiti et al. (**2023**): Synthesis of Unsaturated Bicyclic Lactones via γ-Methylene-C­(sp^3^)–H Activation

In 2023, Yu and co-workers reported a one-step strategy for the synthesis of γ-Arylated γ-lactones from aliphatic carboxylic acids and aryl iodides.^121^ The method employed a pyridone-derived ligand (**L28**, Scheme 36) and enabled the double functionalization of γ-methylene-C­(sp^3^)–H bonds, even in the presence of β-methyl C­(sp^3^)–H bonds.

**Scheme 36:**
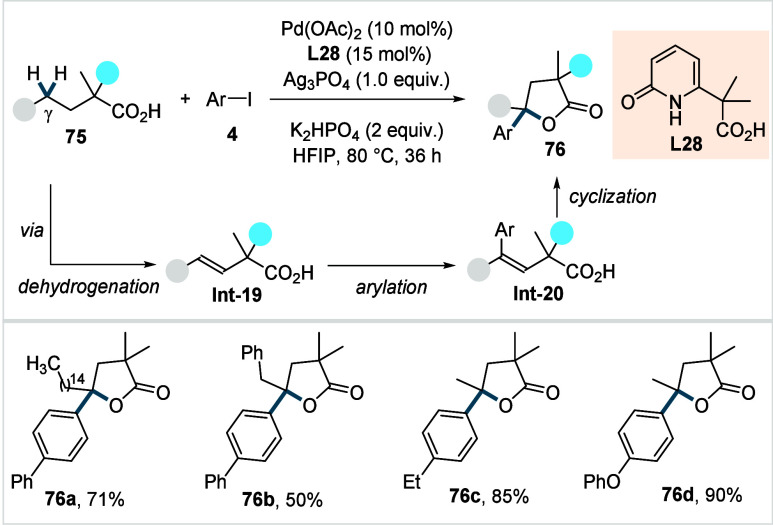
Yu et al. (**2023**): Synthesis of γ-Arylated γ-Lactones via Double γ-C­(sp^3^)–H Functionalization

An H/D exchange study revealed that β-methyl-C­(sp^3^)–H activation is reversible and does not progress further, whereas γ-methylene C­(sp^3^)–H activation is irreversible, leading to rapid product formation and thus accounting for the observed regioselectivity. Mechanistic investigations suggested that the reaction initiates via a β,γ-dehydrogenation to form **Int-19**, along with a Pd^0^-species. A subsequent oxidative addition of aryl iodide (**4**), followed by a 1,2-migratory insertion and β-hydride elimination, produces **Int-20**. Finally, **Int-20** undergoes cyclization to form **76**. A diverse range of aliphatic acids could be coupled with various aryl iodides, affording the respective γ-arylated γ-lactones (**76a**–**76d**) in moderate to excellent yields. However, aliphatic acids bearing α-nonquaternary centers showed poor reactivity using this protocol. 2,2 dimethyl butanoic acid, bearing primary γ-C­(sp^3^)–H bonds, yielded no product, underscoring the catalyst’s selectivity toward γ-methylene over γ-methyl C­(sp^3^)–H bonds.

In their previously reported γ-lactonization study (Scheme 33), Yu et al. identified another quinoline-pyridone-based six-membered chelating ligand that enabled γ-lactone formation via exclusive γ-methylene-C­(sp^3^)–H functionalization (**L27**, Scheme 37).^111^

**Scheme 37:**
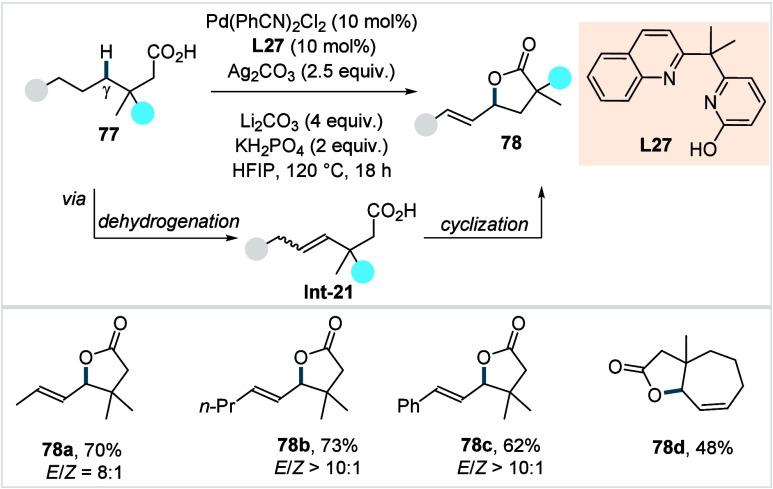
Yu et al. (**2024**): γ-Methylene-C­(sp^3^)–H Lactonization of Aliphatic Carboxylic Acids

An H/D exchange study indicated that **L27** can activate both γ-methylene and γ-methyl C­(sp^3^)–H bonds; however, facile β-hydride elimination from the methylene C–H activated palladacycle outcompetes C­(sp^3^)–O reductive elimination from the methyl C–H activated palladacycle, resulting in exclusive chemoselectivity for γ-methylene C–H lactonization. The reaction was proposed to proceed via γ-methylene-C­(sp^3^)–H palladation followed by β-hydride elimination to generate **Int-21**. This is followed by oxypalladation and β-hydride elimination to furnish **78**. Various aliphatic acids with diverse substitution patterns were compatible under this protocol, affording the respective lactones (**78a**–**78d**) in good yields with high *E*/*Z* selectivity for the newly formed alkene.

In 2006, White and co-workers described a Pd-catalyzed macrolactonizations of ω-alkenoic acids via allylic C–H activation.^122,123^ Using a Pd­(OAc)_2_/phenyl bis-sulfoxide catalyst in combination with benzoquinone (BQ), the reaction proceeds through the formation of a carboxylate directed Pd-templated-π-allyl intermediate (**Int-22**, Scheme 38) that undergoes a subsequent C­(sp^3^)–O reductive elimination to give macrolactonization product **80**. Mechanistic studies revealed that BQ-promoted an inner-sphere C­(sp^3^)–O bond formation. The method furnished a variety of 14–19-membered macrolactones featuring diverse substitution patterns, including *ortho* substituted salicylates (**80a**), cycloalkanes with multiple chiral centers (**80b**), and densely oxygenated acetonide (**80c**) scaffolds. The protocol enabled the synthesis of the macrolactones at a 10 mM concentration, eliminating the requirement for high-dilution conditions typically necessary for macrocyclizations. In 2011, the authors showcased the utility of this strategy method in the late-stage C–H oxidation toward 6-deoxyerythronolide B, a key precursor in the total synthesis of erythromycin.^124^

**Scheme 38:**
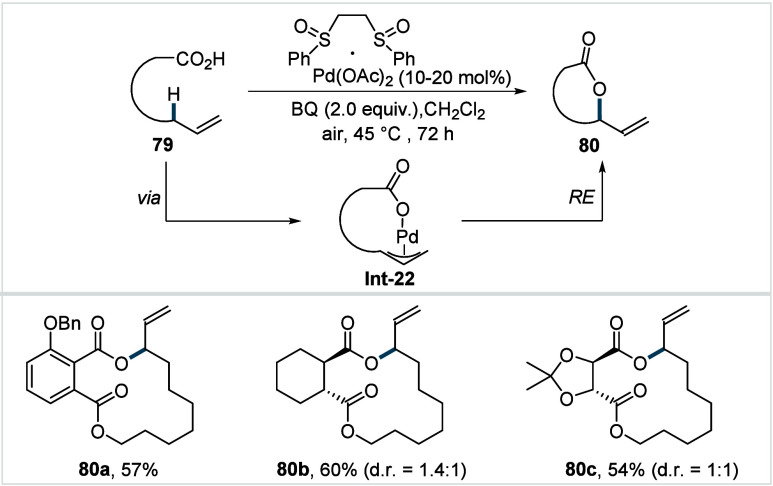
White et al. (**2006**): Macrolactonization via Hydrocarbon Oxidation

### 2.3 Miscellaneous Functionalizations

#### 2.3.1 Methyl-C­(sp^3^)–H Functionalization: Deuterations and Halogenations

In 2021, van Gemmeren and co-workers reported a protocol for the regioselective β-C­(sp^3^)–H deuteration of aliphatic acids.^125^ This transformation was realized based on the reversible nature of C–H activation. Carboxylic acid directed β-C­(sp^3^)–H activation, in which the ligand functions as an internal base, affords **Int-23**. Subsequent functionalization with an electrophilic deuterium source furnishes the corresponding deuterated acids. The method utilizes an ethylenediamine-derived ligand in conjunction with d_1_-HFIP as the deuterium source. A 2,4,6-trialkyl-substituted benzamide moiety on the ligand serving as internal base, outperformed the typically employed acetamide group and was crucial in establishing the protocol (**L29**, Scheme 39). The slight beneficial effect exerted by a catalytic amount of silver salts in this reaction was likely due to its Lewis-acidic nature, which accelerates the C­(sp^3^)–H cleavage step and/or its ability to act as an oxidant, preventing catalyst decomposition via Pd^0^ formation.

**Scheme 39:**
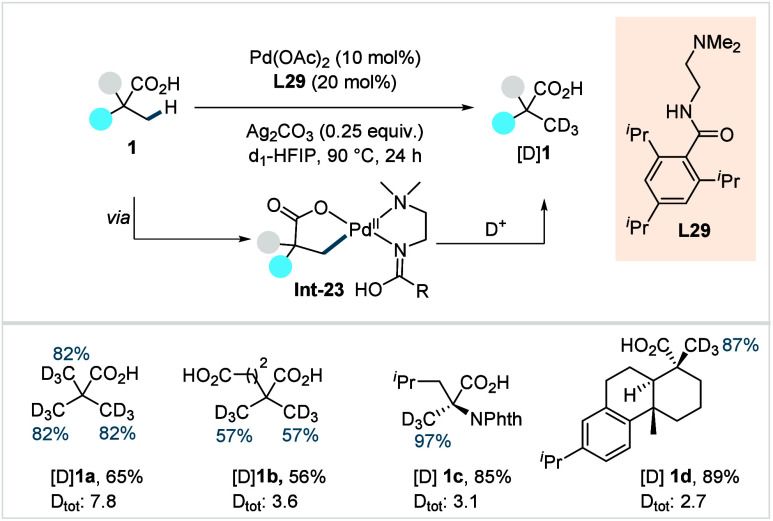
van Gemmeren et al. (**2021**): β-Methyl-C­(sp^3^)–H Deuteration of Aliphatic Carboxylic Acids

The protocol displayed a broad functional group tolerance, enabling the synthesis of a diverse range of isotopically labeled aliphatic acids, including α-nonquaternary acids and bioactive molecule frameworks in good to excellent yields and with high degrees of deuterium incorporation ([D]**1a**–[D]**1d**). The authors also developed an alternative set of reaction conditions using D_2_O as deuterium source, which facilitated hydrogen isotope exchange, albeit with lower efficiency compared to d_1_-HFIP.

Building on the previous studies on pivalic acid bromination by the Yu lab;^126^ Jazzar, Bertrand, Houk, and Yu et al. reported a Pd­(II)-catalyzed β-C­(sp^3^)–H bromination of free carboxylic acids in 2023.^127^ The authors developed a new class of pyridine-based ligands featuring a hydrogen bond donor that interacts with a carboxyl directing group of the substrate, thereby enhancing the substrate–metal affinity. Optimization studies revealed the optimum ligand as a quinoline-derived scaffold with an NHAc group serving as the hydrogen bond donor (**L30** or **L31**, Scheme 40). Importantly, employing an arene as the linker and positioning the NHAc group with an alkyl chain of suitable length at the *meta*-position of the linker were crucial to enable the proposed *meta*-macrocyclophane H-bonding interaction.

**Scheme 40:**
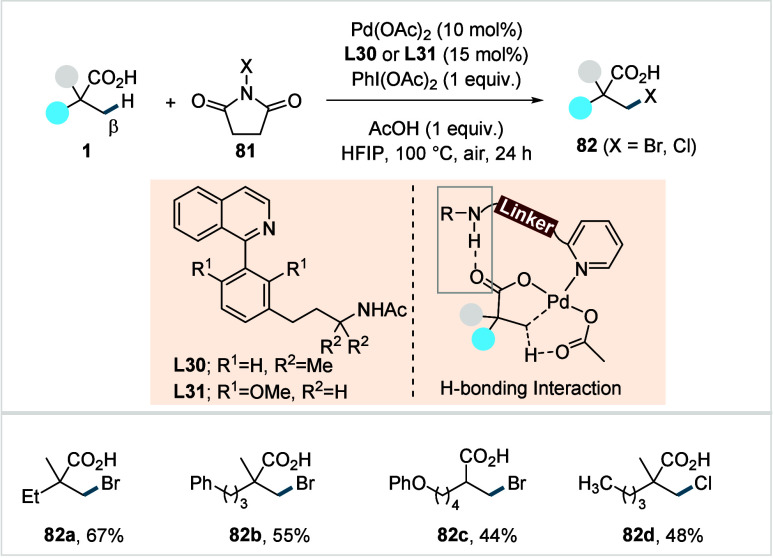
Yu et al. (**2023**): β-Methyl-C­(sp^3^)–H Bromination and Chlorination of Aliphatic Carboxylic Acids

The reaction employed N-bromo succinimide (**81**) as the electrophilic brominating reagent and exhibited a broad functional group tolerance. A variety of α-quaternary acids was converted to the corresponding brominated products in moderate to good yields with exclusive monoselectivity (**82a**–**82b**), while α-nonquaternary acids showed moderate reactivity (**82c**). The authors also demonstrated downstream derivatizations of the brominated products, underscoring the synthetic utility of the methodology. Additionally, the same ligand-scaffold was effective for β-C­(sp^3^)–H chlorination providing the chlorinated products (**82d**) in moderate yields when using N-chloro succinimide as chlorinating reagent.

In the following year, van Gemmeren et al. detailed a protocol for the β-C­(sp^3^)–H fluorination of aliphatic acids using a Pd-catalyst.^128^ The protocol relies on silver fluoride as an operationally simple fluoride source and organic peroxide-based oxidants to promote regioselective fluorination. The reaction is proposed to proceed via a Pd^II^/Pd^IV^ pathway, wherein the initially formed five-membered palladacycle, generated through β-C­(sp^3^)–H activation, undergoes oxidative addition with the peroxide. Subsequent ligand exchange delivers fluoride to the palladium center (**Int-24**), from which reductive elimination furnishes the product. The authors described two sets of reaction conditions: one (Conditions A, Scheme 41) effective for α-quaternary acids, and the other (Conditions B) for α-nonquaternary acids. In Conditions A, a *tert*-butyl benzoyl peroxide bearing a *para*-NO_2_ group proved optimal, whereas a *para*-CF_3_ substituted dibenzoyl peroxide was most effective in Conditions B. Both reaction conditions involve six-membered chelating amino acid-derived ligands featuring an electron-deficient amide as the internal base (**L32** and **L33**). The method showed broad functional group tolerance, providing fluorinated acids in moderate to good yields (**83a**–**83d**). The moderate yields were attributed to incomplete conversion of the starting materials rather than their decomposition.

**Scheme 41:**
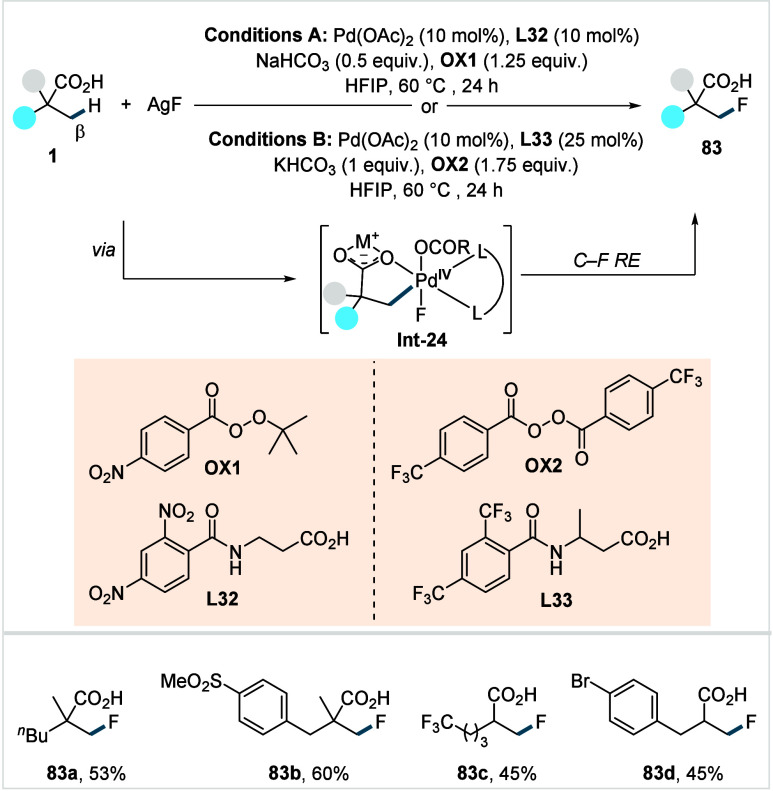
van Gemmeren et al. (**2024**): β-Methyl-C­(sp^3^)–H Fluorination of Aliphatic Carboxylic Acids

A preliminary mechanistic investigation excluded the possibility of β-lactone formation as an intermediate, lending support to a pathway involving a direct C–F reductive elimination from a high-valent Pd^IV^ species.

#### 2.3.2 Methylene C–H Functionalization: Deuteration, Amination, and Desaturation-Based Processes

The van Gemmeren group, in their previously discussed β-C­(sp^3^)–H deuteration study (Scheme 39)^125^ also demonstrated that the same ethylenediamine-derived ligand with 2,4,6-trialkyl substituted benzamide as the internal base was able to promote β-methylene-C­(sp^3^)–H deuteration (**L29**, Scheme 42). Notably, this finding represented the first example of a Pd^II^-catalyzed methylene C­(sp^3^)–H activation and functionalization of structurally unbiased linear aliphatic carboxylic acids. Various substrates, including α-quaternary acids ([D]**39a**), α-nonquaternary acids ([D]**39b**–[D]**39c**) and cycloalkane carboxylic acids ([D]**39d**), as well as bioactive molecules could be deuterated in high yields and with reasonable degrees of deuterium content.

**Scheme 42:**
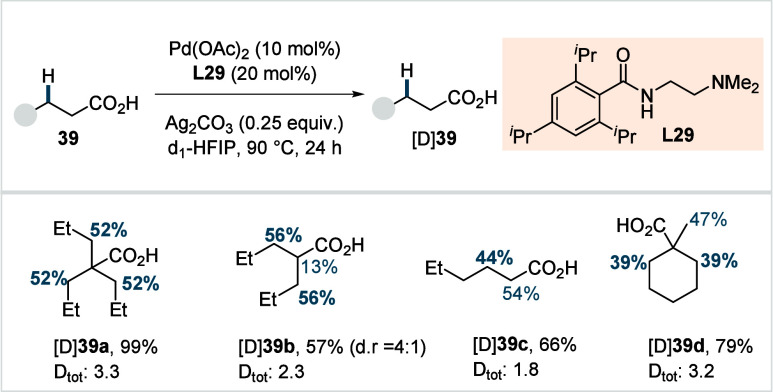
van Gemmeren et al. (**2021**): β-Methylene-C­(sp^3^)–H Deuteration of Aliphatic Carboxylic Acids

In 2024, Yu et al. disclosed a protocol for the intramolecular methylene-C­(sp^3^)–H lactamization and cycloamination of *N*-protected ω-amino acids.^129^ The catalytic system, employing a chlorinated pyridine-pyridone ligand (**L34** and **L35**, Scheme 43), enabled carboxylic acid directed β-methylene C­(sp^3^)–H activation and facilitated a subsequent C­(sp^3^)–N bond formation while suppressing competing amine- and amide-directed processes. The selectivity for acid directed C–H activation over an alternative amide-directed C–H activation was primarily attributed to steric repulsion between the tosyl amide moiety and five-membered chelating ligand **L34** during a potential amide-directed C–H activation process.

**Scheme 43:**
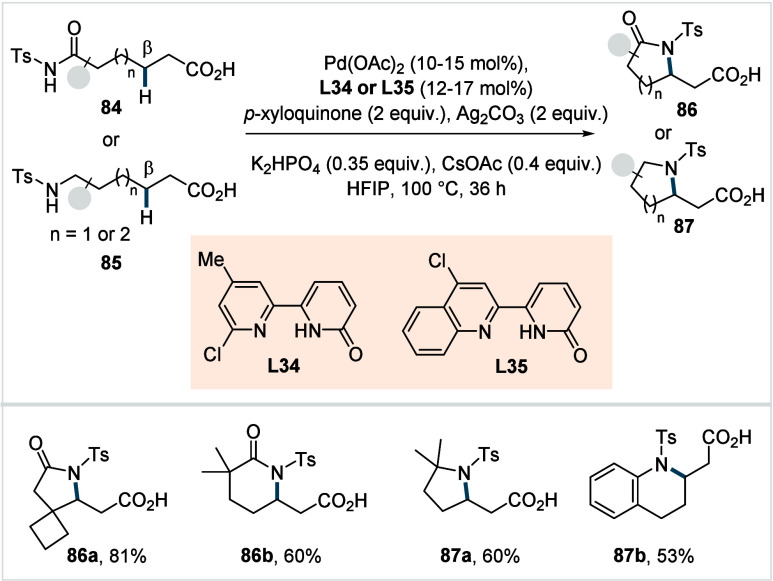
Yu et al. (**2024**): Methylene-C­(sp^3^)–H Lactamization and Cycloamination of Aliphatic Acids

The superior reactivity of **L34** compared to other 4,6-disubstituted pyridine-pyridone derivatives was ascribed to its ability to establish a stronger agostic interaction to the C–H bond of interest in both the prereactive complex and the C–H activation transition state, thereby rendering the rate-determining concerted-metalation-deprotonation process more favorable. A range of γ- and δ-lactams with diverse substitution patterns was synthesized in good yields (**86a**–**86b**). Interestingly, quaternization at the α-position of the acid completely suppressed the reactivity, and subsequent H/D exchange studies indicated that methylene C–H activation was inhibited in the presence of β-methyl C–H bonds. While it is interesting to note these observations with α-quaternary acids, they do not allow for an unambiguous determination if the reaction proceeds via a direct C–N bond formation or through an α,β-desaturation pathway. The method was further extended to synthesize a library of cyclic β-amino acids featuring pyrrolidine and tetrahydroquinoline scaffolds (**87a**–**87b**). However, an analogous piperidine formation remained inaccessible under the reported conditions. The authors furthermore demonstrated the synthetic utility of the protocol through the formal synthesis of stemoamide.

In 2021, Yu and co-workers reported Pd-catalyzed dehydrogenation reactions of free carboxylic acids via methylene C–H activation.^130^ The authors identified a five-membered chelating quinoline-pyridone ligand (**L16**, Scheme 44) that effectively promoted α,β-dehydrogenation of aliphatic acids. Mechanistic investigations revealed that six-membered chelating pyridine-pyridone ligands (bite angle 88.2) provided poor yields due to product inhibition arising from unproductive β-vinylic-C­(sp^2^)–H activation of the initially formed α,β-unsaturated acid. In contrast, five-membered chelating ligands (bite angle 78.7) minimized such inhibition by introducing structural distortion in the corresponding C­(sp^2^)–H cleavage transition state, making them optimal for this transformation. The protocol tolerated a wide range of aliphatic acids, including those bearing branched side chains, saturated heterocycles, and protected hydroxyl groups, affording the α,β-dehydrogenated products with exclusive or predominant *E*-selectivity (**88a**–**88c**).

**Scheme 44:**
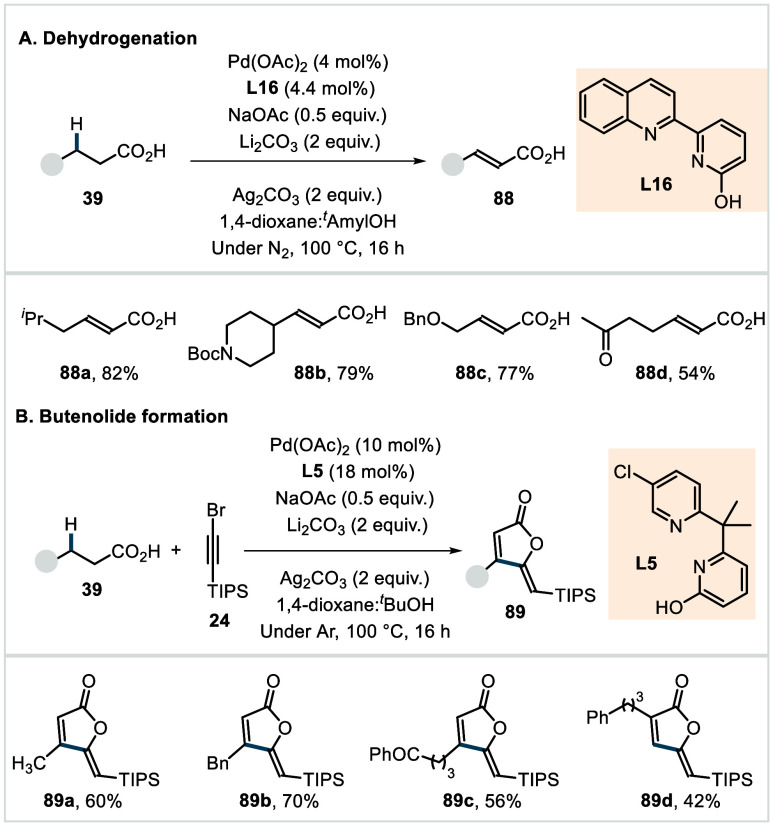
Yu et al. (**2021**): Dehydrogenative Reactions of Aliphatic Acids via Methylene C–H Activation

Furthermore, the selective α,β-dehydrogenation of carboxylic acid was achieved even in the presence of enolizable ketones or esters (**88d**), offering chemoselectivity orthogonal to classical enolate-based methods. Additionally, a second set of reaction conditions enabled the one-step synthesis of γ-alkylidene butenolides from aliphatic acids and bromoalkynes (**24**). This protocol employed a six-membered chelating ligand (**L5**, Scheme 44) and achieved tandem dehydrogenation-alkynylation-cyclization via sequential C­(sp^3^)–H and C­(sp^2^)–H activation. A variety of γ-alkylidene butenolides with diverse substitution patterns was obtained in good yields (**89a**–**89c**). Aliphatic acids bearing β-cycloalkyl substituents showed moderate reactivity. For substrates containing both β-methyl and γ-methylene C–H bonds, a preferential butanolide formation at the methyl C–H bonds was observed (**89d**).

In 2024, Lei et al. provided mechanistic insights through their computational study on Yu’s divergent dehydrogenation (Scheme 44).^77^ For the butenolide formation via dehydrogenative lactonization, the initially formed α,β-unsaturated acid was proposed to undergo C–C coupling via oxidative addition with bromoalkyne, catalyzed by a heterodimeric Pd–Ag species. The final cyclization, leading to C–O bond formation, is then catalyzed by a homodimeric Ag–Ag species.

Later, the same group developed a strategy for the one-step synthesis of β-alkylidene-γ-lactones via β,γ-dehydrogenation of aliphatic acids.^131^ The key to success for this transformation was the discovery of bidentate oxime ether-pyridone and morpholine-pyridone ligands (**L36** and **L37**, Scheme 45), alongside the use of HFIP:MeCN (10:1) as solvent mixture and KF as base. The reaction was proposed to proceed via a tandem dehydrogenation–olefination–cyclization sequence, wherein an initially formed β,γ-dehydrogenated acid (**Int-25**) undergoes carboxylic acid-directed vinylic C­(sp^2^)–H activation/olefination to form **Int-26**, which subsequently cyclizes to afford **91**. Control experiments revealed that the ligand had a beneficial effect on both the β,γ-dehydrogenation and the vinylic C­(sp^2^)–H olefination step.

**Scheme 45:**
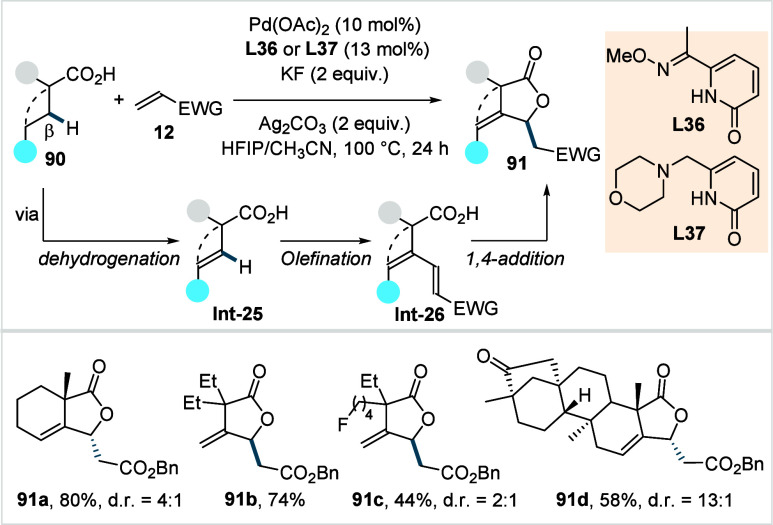
Yu et al. (**2022**): Synthesis of β-Alkylidene-γ-lactones via β,γ-Dehydrogenation of Aliphatic Acids

A range of aliphatic acids, including cycloalkanes with varying ring size, acyclic acids, and bioactive molecules was coupled with various activated olefins to afford the respective lactones in moderate to good yields and with high diastereoselectivities (**91a**–**91d**). Cyclic acids bearing a cyclopentane ring, however, resulted in poor yield of the respective lactones and heterocycles such as piperidine or tetrahydropyran were incompatible with the protocol. It was also noted that α-nonquaternary acids resulted in the formation of α,β-dehydrogenated vinyl olefination products.

Recently, Maiti et al. introduced a method for distal γ,δ-C­(sp^3^)–H functionalization of acyclic carboxylic acids using *N*-acetyl *tert*-leucine as a ligand. In this protocol, acyclic acids featuring a β-quaternary carbon with an aryl group undergo distal dehydrogenative diarylation, followed by intramolecular γ-lactonization, leading to the formation of γ,δ-dehydrogenated γ-lactones. In contrast, substrates with β-alkyl substitution preferentially undergo intermolecular γ,δ-dehydrogenative δ-(di)­arylation.^132^

In the following year, the Yu group reported a one-step strategy for the synthesis of 1,3-dienes via a sequential dehydrogenation of aliphatic acids.^133^ Interestingly, whereas the previously established α,β-dehydrogenation conditions (Scheme 44) failed to furnish dienes, a simple solvent switch from dioxane/*tert*-amyl alcohol (1:4) to CH_3_C­(CF_3_)_2_OH/*tert*-amyl alcohol (4:1) enabled efficient access to desired 1,3-dienes. Systematic ligand modifications revealed a five-membered chelating quinoline-pyridone ligand (**L16**, Scheme 46) to be optimal. The method proved applicable to a range of substrates, with acyclic acids containing cyclic amines, enolizable ketones and esters, affording the respective *E*,*E*-1,3-dienes in moderate to good yields (**94a**–**94c**). Under slightly modified reaction conditions, **L27** promoted sequential dehydrogenation of cycloalkyl carboxylic acids, providing benzoic acid derivatives in synthetically useful yields (**95a**–**95b**).

**Scheme 46:**
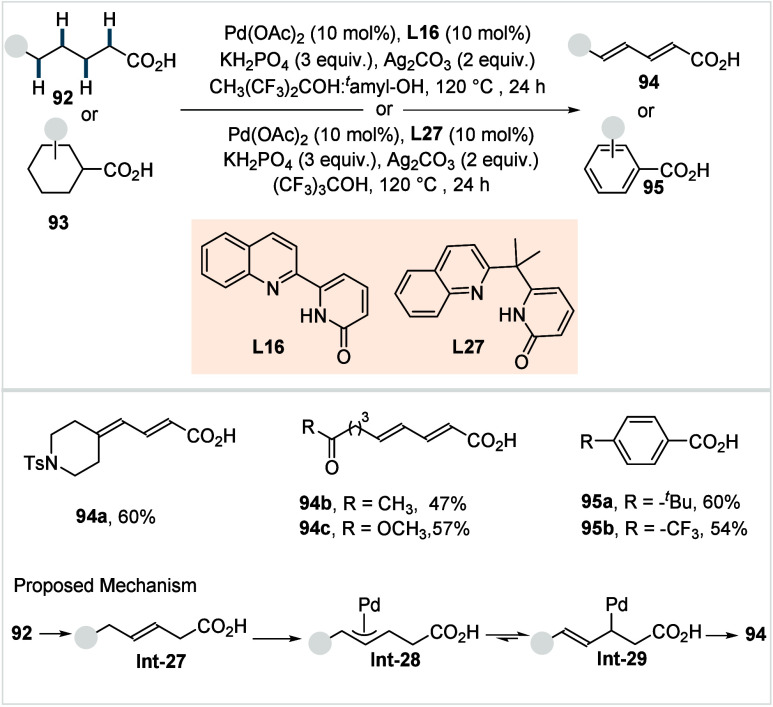
Yu et al. (**2023**): Sequential Dehydrogenation of Aliphatic Carboxylic Acids

In contrast, piperidine carboxylic acids underwent both dehydrogenation and decarboxylation, producing pyridine. The synthetic utility was further highlighted through downstream derivatizations of the conjugated dienes, and by formal syntheses of sorbic acid and a piperine precursor. Based on preliminary mechanistic investigations, the reaction was proposed to proceed via carboxylic acid directed β-methylene-C­(sp^3^)–H activation, followed by β,γ-dehydrogenation to give **Int-27**. From this stage, formation of an allyl-Pd species (**Int-28** and **Int-29**) and subsequent β-hydride elimination affords the diene product (**94**).

In 2024, Ge and Maiti et al. reported a protocol using an *N*-acetyl valine ligand (**L14**, Scheme 47) to access olefinated arenes directly from cyclohexane carboxylic acids via sequential dehydrogenation-olefination-decarboxylation-aromatization.^134^ Based on mechanistic experiments, the reaction was proposed to proceed via a Pd^II^/Pd^0^ cycle, wherein ligand-enabled β-methylene-C­(sp^3^)–H activation, followed by site-selective dehydrogenation forms an β,γ-unsaturated intermediate **(Int-30)**. Subsequent carboxylic acid directed vinylic olefination yields **Int-31**, which then undergoes decarboxylation with olefinic migration to generate **Int-32**. Finally, sequential allylic C–H activation and dehydrogenation delivers **97**. A range of alkyl substituted cyclohexyl carboxylic acids provided the corresponding olefinated arenes in good yields (**97a**–**97c**). However, 1-, and 2-substituted cyclohexanoic acids showed attenuated reactivity. Interestingly, cyclohexanoic acids bearing hydroxy or 4-acetoxy groups provided dehydroxylated or deacetoxylated olefinated arenes. The method proved compatible with various olefinic partners, including activated olefins, styrenes, and allyl alcohols. Cycloalkane carboxylic acids that could not undergo aromatic stabilization instead formed difunctionalized cycloalkenes (**100a**–**100c**) through a sequence of dehydrogenation–olefination–decarboxylation–allylic acyloxylation. Multicomponent reactions involving two different acid partners, yielded primarily the desired heterocoupled products, with homocoupled byproducts being observed to a minor extent. The synthetic utility of this protocol was further demonstrated through the late-stage functionalization of drug molecules and the preparation of key intermediates in the synthesis of bioactive compounds.

**Scheme 47:**
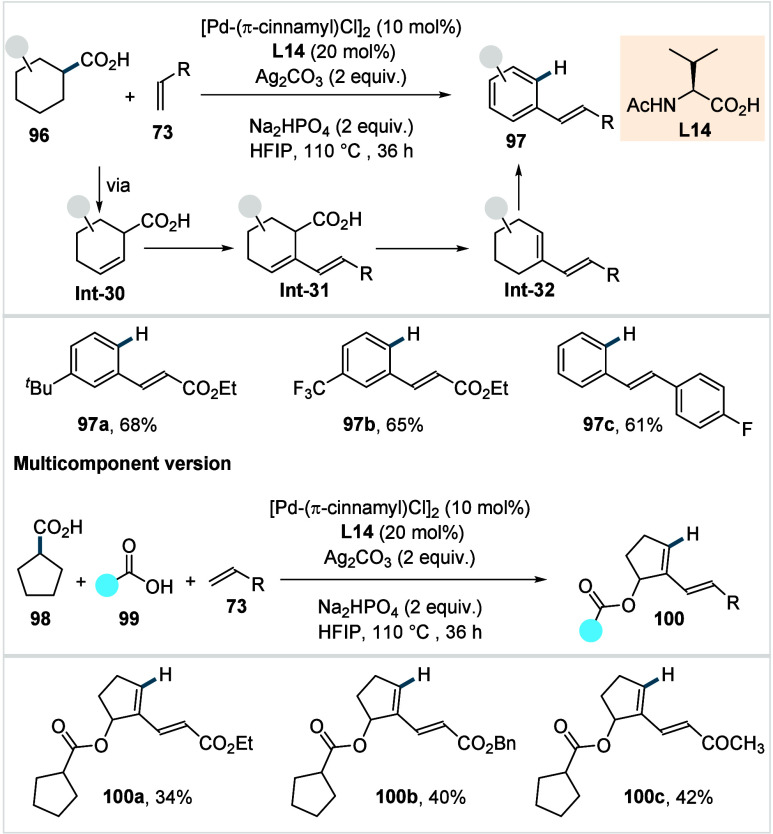
Maiti et al. (**2024**): Tandem Dehydrogenation-Olefination-Decarboxylation of Cycloalkyl Carboxylic Acids via Multifold C–H Activation

In 2023, The Yu group developed a strategy for the synthesis of β,γ-unsaturated aliphatic acids via dehydrogenation of methylene C­(sp^3^)–H bonds.^135^ Optimization studies identified two classes of six-membered chelating ligands, quinuclidine-pyridone (**L19**, Scheme 48) and pyridine-pyridone (**L38** and **L39**) to be optimal. Interestingly, the use of HFIP:CH_3_CN (10:1) as solvent mixture was crucial for reactivity, as no desired product was obtained in the absence of acetonitrile. The method displayed remarkable substrate generality, furnishing β,γ-unsaturated acyclic (**102a**) and cyclic acids spanning from five-membered (**102b**) to macrocyclic frameworks, as well as derivatives of complex bioactive terpene natural products (**102c**) in good to excellent yields. For cyclic substrates, dehydrogenation occurred exclusively within the cycloalkane rings (**102b**), even in the presence of additional potentially reactive β- and γ-positions. Furthermore, aliphatic acids possessing acidic α-hydrogen substituents underwent selective β,γ-dehydrogenation, delivering the respective products in moderate yields (**102d**).

**Scheme 48:**
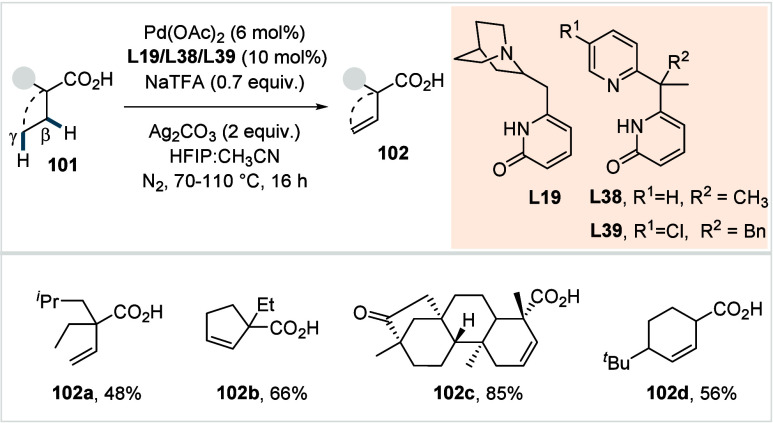
Yu et al. (**2023**): β,γ-Dehydrogenation of Aliphatic Carboxylic Acids

An H/D exchange study with **101d** revealed that the C–H activation is reversible, occurring preferentially at the β-position in a syn-orientation relative to the directing group. It is followed by a syn-stereospecific β-hydride elimination occurring at the γ-position only, which results the observed site selectivity. The synthetic utility was further demonstrated by transforming the β,γ-double bond into diverse functional motifs, including epoxides, aziridines, and β-lactones.

In 2025, Yu et al. detailed a protocol for the one-step synthesis of butenolides directly from aliphatic carboxylic acids via a one-pot functionalization of three C­(sp^3^)–H bonds.^136^ The method is enabled by a triazole-pyridone-based ligand (**L40**, Scheme 49) and TBHP as the sole oxidant. The reaction was proposed to proceed via a Pd^II^/Pd^0^/Pd^II^/Pd^0^ cycle, wherein ligand-accelerated β,γ-dehydrogenation forms **Int-33**, with the concomitant formation of Pd^0^. Reoxidation by TBHP generates Pd^II^, which then promotes a 5-endo-trig-type cyclization, followed by site-selective β-hydride elimination to give **104**. The use of benzoquinone derivatives duroquinone or anthraquinone-2,6-disulfonic acid disodium salt was proposed to suppress Pd black formation by coordinating to Pd^0^. The reaction featured broad functional group tolerance and enabled the preparation of structurally diverse butenolides, including those with amides (**104a**), protected alcohols (**104b**), and heterocycles (**104c**) in good yields. Additionally, a variety of spirocyclic (**104d**) and macrocycle-bridged butenolides was efficiently synthesized. Aliphatic acids lacking α-substitution gave moderate yields of the respective butenolides due to a competing α,β-dehydrogenation pathway. Lastly, the synthetic utility was highlighted by the efficient total or formal synthesis of 12 bioactive natural products and drug molecules, including anticancer and anti-HIV agents.

**Scheme 49:**
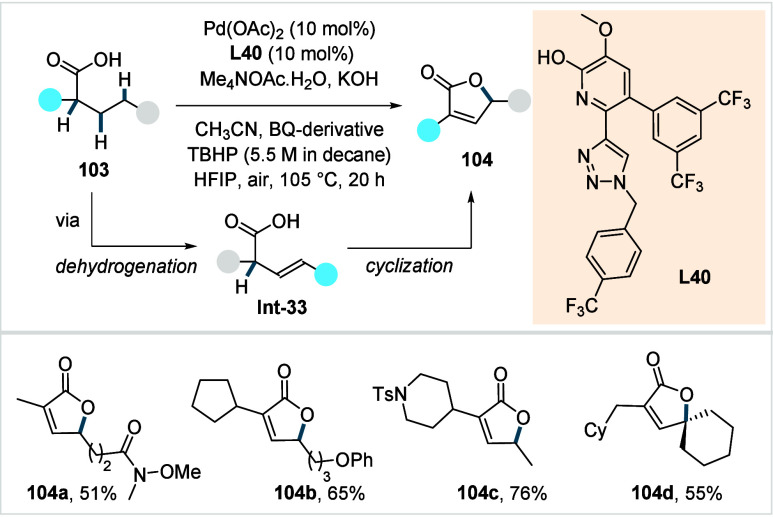
Yu et al. (**2025**): Syntheses of Butenolides via Triple C­(sp^3^)–H Functionalization of Aliphatic Carboxylic Acids

Recently, Maiti et al. developed a protocol based on an amino acid-derived ligand that involves a Pd^II^-catalyzed γ-C­(sp^3^)–H activation and a strain-promoted C–C bond cleavage to achieve the skeletal reorganization of cyclopropane carboxylic acid derivatives, thereby enabling efficient access to a diverse library of 3-oxabicyclo[3.1.0]­hexan-2-one scaffolds.^137^

While most progress in carboxylic acid directed C­(sp^3^)–H activation and functionalization has centered on methyl and methylene sites, the Yu group recently described a protocol for the synthesis of arylated bicyclo[3.2.0]­heptane lactones via β-methine-C­(sp^3^)–H activation of bicyclo [1.1.1] pentane carboxylic acid.^138^

### 2.4 Ligand Design in Carboxylic Acid Directed C–H Activation of Aliphatic Acids

Following the seminal discovery of the countercation effect as a foundational element in C­(sp^3^)–H activation of free carboxylic acids (Scheme 1A), the rational design and identification of external ligands have dramatically expanded the scope and efficiency of this transformation. The success of ligand-accelerated catalysis is largely attributed to the weakly coordinating nature of carboxylate, which minimizes competitive binding at the Pd center and thereby facilitates incorporation of external ligands. Ligand-accelerated C–H activation is governed by multiple interrelated design principles. Among the most crucial considerations are the reduction of the activation barrier for concerted metalation–deprotonation (CMD)^54,139–141^ step and the stabilization of Pd^II^ species by suppressing reduction to Pd^0^ and controlling speciation equilibria to prevent the formation of inactive species.^142^ These factors operate in concert with additional constraints arising from the coordination geometry. For example, Pd­(OAc)_2_ exists in solution predominantly as an acetate-bridged trimer, necessitating ligand-promoted dissociation to generate catalytically competent mononuclear species. Given the square-planar geometry of Pd^II^, ligand architectures must preserve two adjacent coordination sites to enable substrate binding and subsequent C–H cleavage. Collectively, these mechanistic and structural considerations have driven the development of both monodentate and bidentate ligand architectures. Monodentate σ-donor ligands such as pyridines facilitate trimer dissociation but frequently generate coordinatively saturated mononuclear Pd complexes that disfavor ligand exchange, thereby diminishing catalytic efficiency. Sterically modified pyridines bearing 2,6-substituents mitigate this issue by promoting dynamic ligand dissociation through steric repulsion between the ligands, thereby favoring complexes with a 1:1 Pd:L ratio over complexes with a 1:2 ratio and thus enabling substrate coordination while retaining sufficient metal stabilization (Approach 1, Scheme 50).^56,126^

**Scheme 50:**
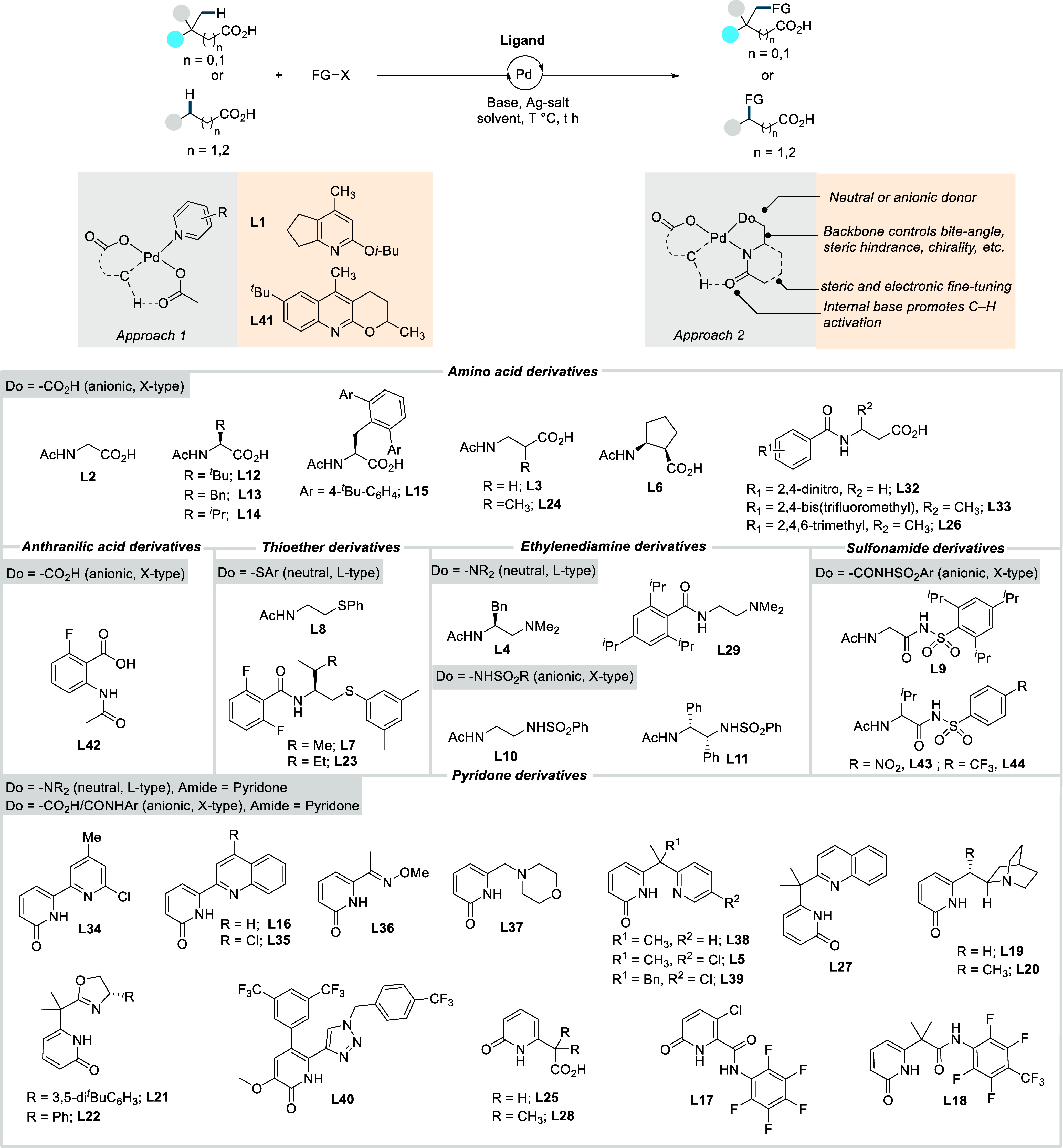
Ligand Design in the Carboxylic Acid Directed C–H Activation of Aliphatic Acids

As an expansion of this approach, monodentate ligands bearing a pendant NHAc functionality capable of hydrogen bonding with the carboxylate ligand in a *meta*-macrocyclophane-like arrangement have been shown to enhance substrate–catalyst interactions.^127^ Monodentate ligand-enabled C–H activation with simple Pd^II^ salts relies on acetate or carbonate ligands acting as internal bases in the CMD step, suggesting that the incorporation of related basic functionalities into the ligand itself could accelerate the CMD process. This concept motivated the development of bidentate ligands, wherein chelation enhances catalyst stabilization while enforcing a *cis* relationship between the vacant coordination sites as required for substrate coordination and subsequent C–H cleavage (Approach 2, Scheme 50). The discovery of *N*-acyl amino acid-derived ligands by the Yu lab was particularly influential, marking the starting point for all research on the design of bidentate ligands containing an internal base motif. The amidate functionality contained in these ligands, serves as internal base for the CMD step, the trivalent nature of nitrogen providing the required point of attachment to the ligand backbone.^143^

Many bidentate ligands feature an X-type amidate donor paired with either a neutral (L-type) or an anionic (X-type) auxiliary donor. Beyond modulating steric and electronic properties, the donor moiety positions the amidate unit proximal to the C–H activation site. The ligand backbone governs rigidity, steric hindrance, bite angle and, in chiral systems stereochemical information. Building upon this platform, diverse bidentate ligand classes have emerged, including amino acid-derived (X,X-type) ligands with variable ring sizes and internal base motifs (**L2**–**3**, **L6**, **L12**–**15**, **L24**, **L26**, **L32**–**33**);^128,144^ anthranilic acid-derived (X,X-type)^82^ ligands featuring rigid phenylene linkers (**L42**); thioether-based (L,X-type) systems featuring neutral sulfur donors (**L7**–**8**, **L23**);^70,125^ ethylenediamine-derived (L,X and X,X-type) frameworks bearing either neutral (**L4**, **L29**)^68^ or anionic donors (**L10**–**11**);^74^ and N-acyl sulfonamide-based (X,X-type) ligands (**L9**, **L43**–**44**)^73,91^ that allow systematic tuning of donor p*K*_a_ and steric profile. Collectively, these scaffolds have demonstrated particular efficacy in the activation and functionalization of methyl C­(sp^3^)–H bonds.

A key advance in bidentate ligand design has been the use of pyridone units as internal bases in conjunction with either a neutral, strongly σ-donating L-type donor or an anionic X-type donor. This ligand design has once more proven to be highly flexible with neutral donors that have been used successfully in this context including aromatic N-heterocycles (**L5**, **L16**, **L27**, **L34**–**35**, **L38**–**40**), oxime ethers (**L36**), amines (**L19**–**20**, **L37**), and oxazolines (**L21**–**22**), and anionic donors including carboxylate (**L25**, **L28**) and amidate units (**L17**–**18**). These ligands have demonstrated notable effectiveness in methylene C­(sp^3^)–H functionalization.^142,145^ This can be rationalized by the aromatization of the pyridone to give a hydroxypyridine during the concerted metalation-deprotonation process, which provides a favorable energetic contribution to this key step.

## 3 C–H Functionalization Reactions of Aliphatic Acids via Hydrogen Atom Transfer (HAT)

Complementary to C–H functionalization via C–H activation, alternative mechanisms exploiting the carboxylic acid moiety as directing group have as well been investigated. Radical-mediated C–H functionalization reactions, often involving organic radicals and radical-like high-valent metal-oxo species, proceed via a hydrogen atom transfer (HAT) pathway. The selectivity in these reactions is primarily governed by homolytic cleavage of C–H bond, generating alkyl radical intermediates (**Int-34** or **Int-35**, Scheme 51). Consequently, the relative reactivity of C–H bonds toward HAT decreases in the order: 3 °C–H > 2 °C–H ≫ 1 °C–H, mirroring trends in C–H bond dissociation energies (BDEs): This trend is inverse compared to C–H activation processes, such that the processes discussed in this chapter often display complementary regioselectivity patterns. to those presented in Section 2.

**Scheme 51:**
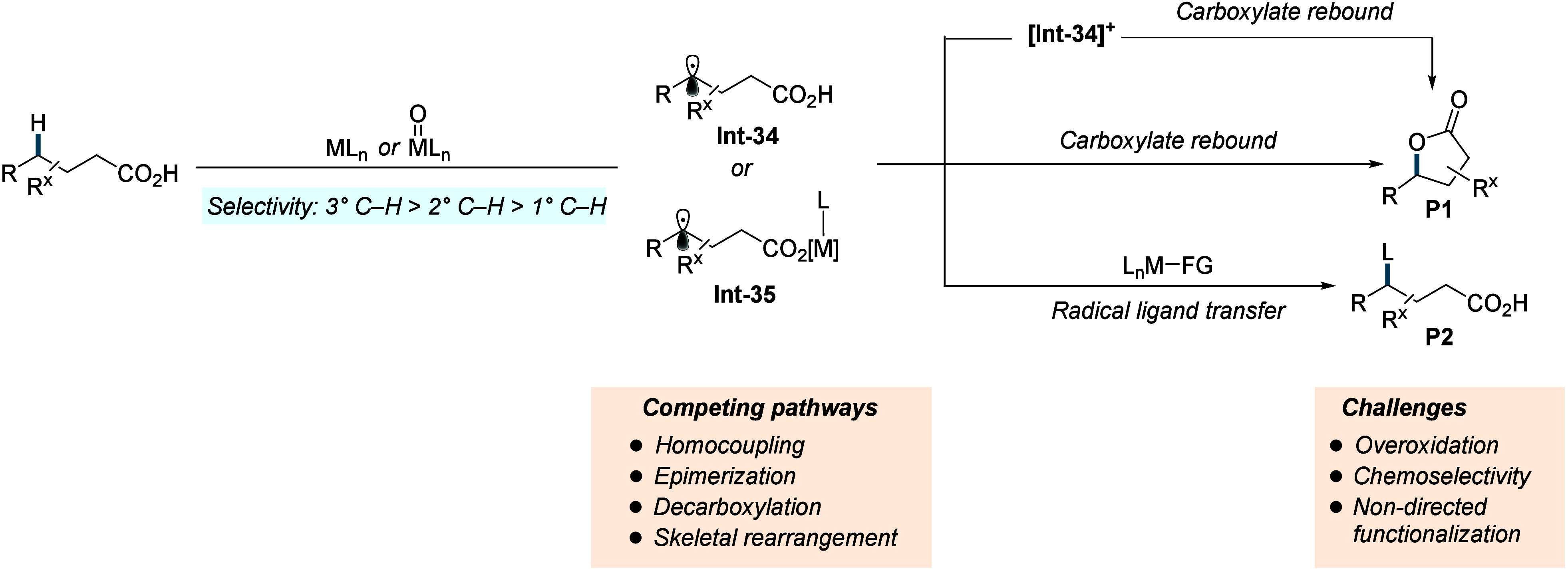
General Reaction Pathways and Associated Challenges in the C­(sp^3^)–H Functionalization via Radical Intermediates

The formation of a carbon-centered radical can occur in a nondirected manner, producing **Int-34**. This intermediate undergoes lactonization via carboxylate rebound, either directly or through the generation of a transient carbocation (**[Int-34]** ^**+**^), yielding the lactone product (**P1**). While such processes are strictly speaking not carboxylic acid directed, they require the presence of the carboxylic acid moiety in a defined position relative to the site of radical generation. Considering that in some cases the involvement of **Int-34** or **Int-35** remains ambiguous and processes designed to be carboxylic acid directed were sometimes discovered to proceed through **Int-34**, a number of such examples is included in this chapter, also to provide historic context to some developments, but the main focus remains on truly acid-directed methods. In such methods, the radical generation is accompanied by an interaction between the transition metal catalyst and the carboxylate moiety, leading to intermediates **Int-35**, bearing carboxylate-bound metal-species. Such species can undergo either carboxylate rebound to yield a lactone product (**P1**) or radical ligand transfer^146^ to afford a C–L bond between the former radical and a ligand previously bound to the catalyst (L = OH or halide, **P2**).^147^ Such transformations face a number of inherent challenges. First, metal-bound carboxylates have a tendency to undergo decarboxylation via homolytic cleavage of the O–M bond, leading to the formation of alkyl radicals, which then further decompose. Although undesired in this context, it should be noted that such processes have also been exploited to enable useful transformations.^148,149^ Second, the high reactivity of the carbon-centered radical generated during the course of the desired transformations introduces competing pathways, such as homocouplings and undesired skeletal rearrangements. Additional challenges include overoxidation, for example initially formed secondary alcohols are susceptible to oxidation at the α-C–H bond to produce ketones,^147^ and chemoselectivity, for example when the directed functionalization of a targeted C­(sp^3^)–H bond competes with the functionalization of an intrinsically more reactive one. Finally, a potential stereospecific C–H functionalization requires a sufficiently short radical lifetime to prevent epimerization between hydrogen atom transfer and the rebound step.^150^ In light of these challenges it is not surprising that catalysts with finely tuned electronic and steric properties, alongside an appropriate selection of solvent and reaction conditions, are essential to mitigate these issues and achieve the desired outcome.

### 3.1 Allylic/Benzylic-C­(sp^3^)–H Functionalization

In 2003, Mahmoodi and co-workers disclosed a protocol for the synthesis of γ-substituted phthalides from *ortho*-aryl benzoic acids in aqueous medium using stoichiometric copper chloride and sodium persulfate as oxidant.^151^ The reaction was proposed to proceed via a radical pathway involving a benzylic intermediate of type **Int-34**, and delivered a range of γ-substituted phthalides in moderate to good yields.

In 2024, Du Bois et al. reported a protocol for the oxidative cyclization of carboxylic acids to access lactone products.^152^ The reaction employed catalytic amounts of Cu­(OAc)_2_ with potassium persulfate as the terminal oxidant and was conducted in aqueous acetic acid. Mechanistic studies suggested a pathway initiated by a sulfate radical (SO_4_^•–^) mediated C–H abstraction to generate radical intermediate **Int-34a** (Scheme 52). The nondirected nature of the C–H abstraction, was evidenced by control experiments. In the presence of Cu^II^, **Int-34a** is then trapped and/or oxidized to the corresponding carbocation **([Int-34a]** ^**+**^ **)**, which subsequently undergoes cyclization with carboxylate to furnish lactone **106**. Interestingly, While Cu­(OAc)_2_ was indespensable for turnover, it had negligible impact on product selectivity.

**Scheme 52:**
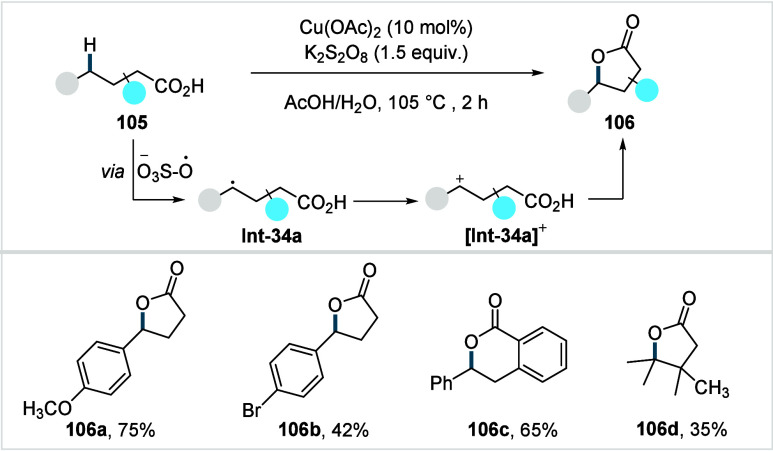
Du Bois et al. (**2016**): Copper Catalyzed Oxidative Cyclization of Aliphatic Acids

Aryl-tethered carboxylic acids afforded the respective five-membered lactones (**106a**–**106b**) in moderate to good yields, with electron-rich arenes (**106a**) proving superior to their electron-deficient counterparts (**106b**). Interestingly, benzoic acid substrates with arene substitution in the 5- and 6-position (and consequently very similar BDEs) favored the formation of six-membered lactones (**106c**). The method was particularly suited for benzylic C–H functionalization and, to a lesser extent, could be applied to tertiary C­(sp^3^)–H bonds in conformationally constrained aliphatic systems, affording the corresponding lactones in moderate yields (**106d**).

In 2024, Yu and co-workers disclosed a strategy for the synthesis of γ-lactones that enabled the functionalization of a broad range of C­(sp^3^)–H bonds with different electronic properties, including benzylic and allylic sites.^153^ The protocol employs a copper catalyst with Selectfluor as the oxidant and proceeds in the fluorinated solvent HFIP (Scheme 53). The reaction was proposed to operate via a Cu^I^/Cu^II^ catalytic cycle, in which Cu^I^ is oxidized by Selectfluor to form Cu^II^ with concurrent generation of an N-centered radical, which promotes nondirected HAT to produce radical intermediate **Int-34a**. A subsequent oxidation by Cu^II^ furnishes a carbocation species (**[Int-34a]** ^**+**^), which undergoes intramolecular cyclization with carboxylate to give lactone **106**.

**Scheme 53:**
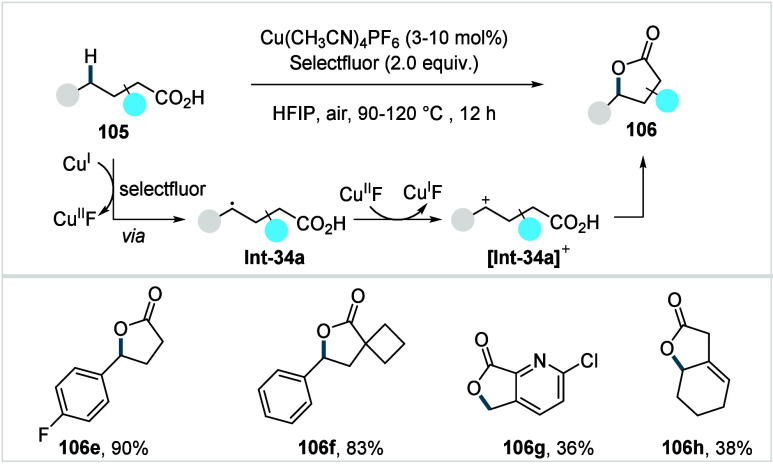
Yu et al. (**2024**): Copper Catalyzed Benzylic and Allylic C­(sp^3^)–H Lactonization of Aliphatic Acids

A variety of benzo-fused lactones, including cases with electron-deficient arenes (**106e**), a strained cyclobutane ring at the α-position (**106f**), and electron-deficient heteroarenes (**106g**), was obtained in moderate to excellent yields. Polar functional groups such as nitro, primary amine, and sterically unhindered pyridines were found to be incompatible with the protocol. Additionally, allylic C­(sp^3^)–H bonds despite being adjacent to a reactive double bond, delivered the respective lactones in moderate yields (**106h**).

### 3.2 Tertiary (3°)-C­(sp^3^)–H Functionalization

In 2011, White et al. demonstrated that a small-molecule, non-haem iron-catalyst can promote mixed hydroxylase activity with aliphatic substrates bearing carboxylic acids.^154^ Batchwise addition of Fe­(PDP) catalyst and H_2_O_2_ enabled an efficient oxidation in acetonitrile (Scheme 54), furnishing lactone and hydroxylactone products. Cycloalkane carboxylic acids bearing tertiary C­(sp^3^)–H bonds predominantly yielded butyrolactones (**108a**–**b**) alongside minor hydroxylactones (**109a**–**b**). The oxidation of enantioenriched alkanoic acid (**108c**) proceeded with complete retention of stereochemistry and the method was compatible with highly functionalized frameworks such as a picrotoxinin-derivative (**108d**).

**Scheme 54:**
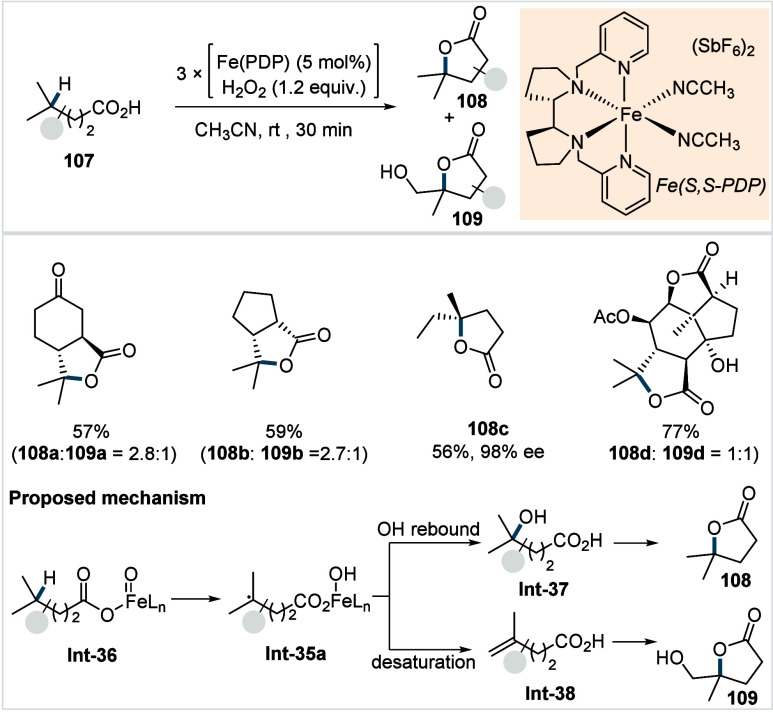
White et al. (**2011**): Non-haem Iron Catalyzed Hydroxylation and Desaturation of Aliphatic C–H Bonds

Mechanistic studies supported a pathway involving a high-valent iron-oxo carboxylate species (**Int-36**), which enables hydrogen abstraction to generate a carbon-centered radical species **Int-35a**. This short-lived intermediate diverges along two pathways: hydroxyl rebound, a radical ligand transfer process involving a hydroxo ligand, delivers lactones (**108**) via **Int-37**, while further oxidation and deprotonation affords desaturated intermediates (**Int-38**), which undergo epoxidation and intramolecular lactonization to form hydroxylactones (**109**).

In the subsequent year, the same group described a general strategy harnessing the directing effect of the carboxylate moiety in a metal­(oxo)-promoted C­(sp^3^)–H hydroxylation/lactonization.^155^ The mechanism of this Fe­(PDP)-catalyzed process is analogous to the previously discuss hydroxylation pathway (Scheme 55). An ^18^O-labeling study supported the formation of **111** from **Int-35b** via a hydroxyl rebound followed by lactonization, rather than through a carboxylate rebound pathway. The highly electrophilic, sterically encumbered Fe­(PDP)-oxo-species preferentially targeted electron-rich and sterically accessible C­(sp^3^)–H bonds.

**Scheme 55:**
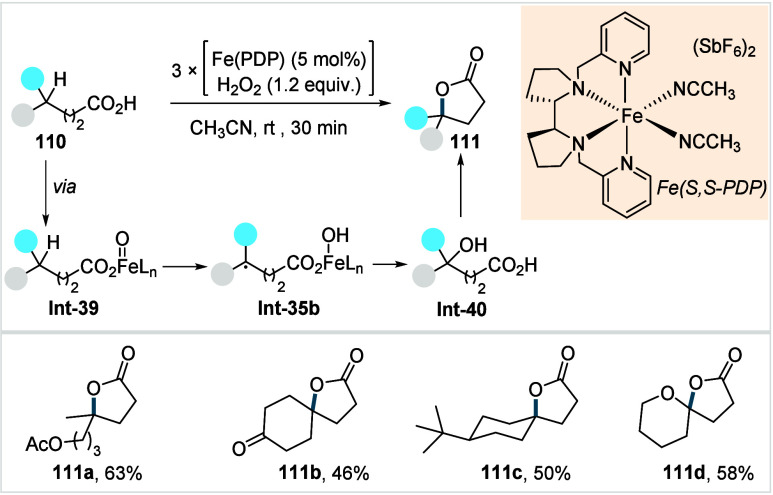
White et al. (**2012**): Directed Metal­(oxo) Aliphatic C–H Hydroxylation

Carboxylate ligation to Fe­(PDP) was crucial in overriding the electronic, steric, and stereoelectronic biases that typically dominate in nondirected oxidations. Consistent with these effects, the protocol enabled selective oxidation of tertiary C–H bond located remote from an electron-withdrawing acetoxy group (**111a**) and a carbonyl moiety (**112b**). Likewise, the sterically hindered axial C–H bond in **111c** and the tertiary ethereal C–H bond in the presence of secondary ethereal C–H bond in **111d** were efficiently and selectively functionalized. In contrast, the corresponding methyl esters of **110** showed poor reactivity, underscoring the importance of carboxylate coordination for both reactivity and selectivity. The utility of the protocol was further highlighted by the C-2 oxidation of the paclitaxel framework.

In the lactonization strategy developed by the Yu group discussed above (*cf*. Scheme 53),^153^ tertiary C–H bonds were also shown to undergo efficient functionalization. A diverse library of lactones containing synthetically useful functional groups such as hydroxy (**113a**, Scheme 56), phthalimide (**113b**), and spirocyclic scaffolds (**113c**) was obtained in modest to excellent yields. Interestingly, replacing the copper catalyst with Pd­(CH_3_CN)_2_Cl_2_, which is known to undergo single-electron transfer (SET) process with Selectfluor to generate a Selectfluor radical dication,^156^ proved crucial for specific substrates, particularly those bearing cycloalkane rings at the γ-position (**113c**). Furthermore, aliphatic acids possessing tertiary δ-C–H bonds underwent smooth oxidation to provide the corresponding δ-lactones (**113d**).

**Scheme 56:**
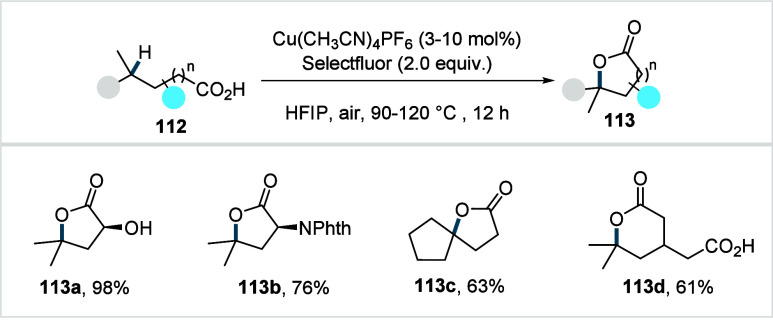
Yu et al. (**2024**): Copper Catalyzed 3° C­(sp^3^)–H Lactonization of Aliphatic Acids

### 3.3 Secondary (2°)-C­(sp^3^)–H Functionalization

In 2007, White et al. employed an Fe­(*S*,*S*-PDP) catalyst to achieve site-selective 2°-C­(sp^3^)–H oxidation of a tetrahydrogibberellic acid analogue.^157^ An iterative addition of the Fe-catalyst and H_2_O_2_ furnished the corresponding five-membered lactone in 52% overall yield after a single recycling of the recovered starting material. Subsequently, the group developed a slow addition protocol in which the Fe-catalyst and H_2_O_2_ were simultaneously added over 45–60 min via a syringe pump, delivering the same lactone in 51% yield without the need for recycling.^158^ In 2010, the authors extended their Fe­(PDP)/H_2_O_2_-based oxidation methodology to a pleuromutilin derivative, achieving diastereoselective hydroxylation of the more electron-rich and sterically accessible equatorial methylene C­(sp^3^)–H bond of the cyclohexane ring.^159^

While Fe-based catalysts have proven highly effective for the functionalization of tertiary C­(sp^3^)–H bonds, their application to secondary C­(sp^3^)–H sites often resulted in either poor yields of the respective lactones or undesired overoxidation to ketoacids. In 2020, Bietti and Costas et al. developed a protocol for the enantioselective C–H oxidation of unactivated methylene C­(sp^3^)–H bonds in aliphatic acids to form lactones.^160^ The transformation, catalyzed by a chiral Mn-complex in the presence of H_2_O_2_, proceeded efficiently in a fluorinated alcohol solvent (Scheme 57). The addition of a catalytic amount of triflic acid proved beneficial, presumably by promoting the heterolytic cleavage of the O–O bond in H_2_O_2_ to generate the reactive Mn-oxo species. The use of (*S*,*S*)-Mn­(^TIBS^PDP) enabled the lactonization of adamanataneacetic acid and 2,2-dimethyladamantaneacetic acid (**115a**) in high yield and with excellent enantioselectivity. The presence of hydrogen bond acceptor (HBA) groups (hydroxy, ether, amide, etc.) at the 3′ position favored distal over proximal lactonization.

**Scheme 57:**
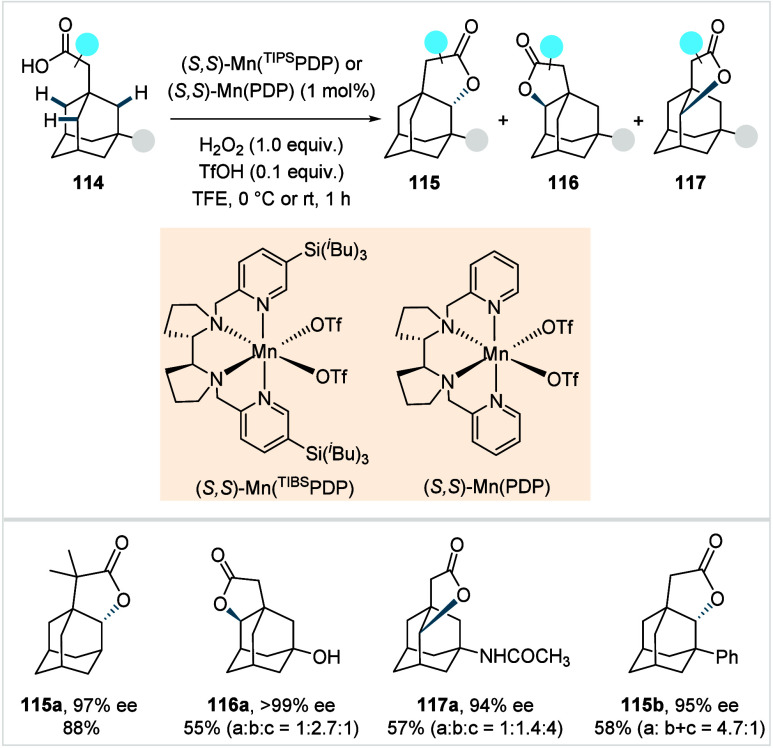
Costas et al. (**2020**): Enantioselective Methylene C–H γ-Lactonization of Adamantaneacetic Acid Derivatives

For the distal lactonization, the sterically encumbered (*S*,*S*)-Mn­(^TIBS^PDP) preferred oxidation of the *exo* C–H bonds (**116**), whereas less sterically hindered (*S*,*S*)-Mn­(PDP) preferentially oxidized *endo* C–H bonds (**117**). A range of synthetically useful substituents, including hydroxy (**116a**), amide (**117a**), and ester groups, was well tolerated, affording the respective major lactone isomers in good yields and excellent enantioselectivities. In contrast, substrates bearing non-HBA functional groups at the 3′ position favored proximal lactonization. A diverse library of proximal lactones, including those featuring electronically different phenyl substituents (**115b**), was prepared in good yields and high enantioselectivities for the major isomer. Lastly, the author successfully applied their strategy to the synthesis of a chiral bicyclo [3.3.1] nonane.

In the subsequent year, the authors introduced a protocol for the site-selective γ-methylene-C–H lactonization of natural and unnatural α-amino acids.^161^ Using the (*S*,*S*)-Mn­(^TIPS^PDP) catalyst, which features trialkylsilyl-substituted pyridine moieties, together with H_2_O_2_ as the oxidant, the reaction proceeded under mild conditions and within short reaction times (Scheme 58).

**Scheme 58:**
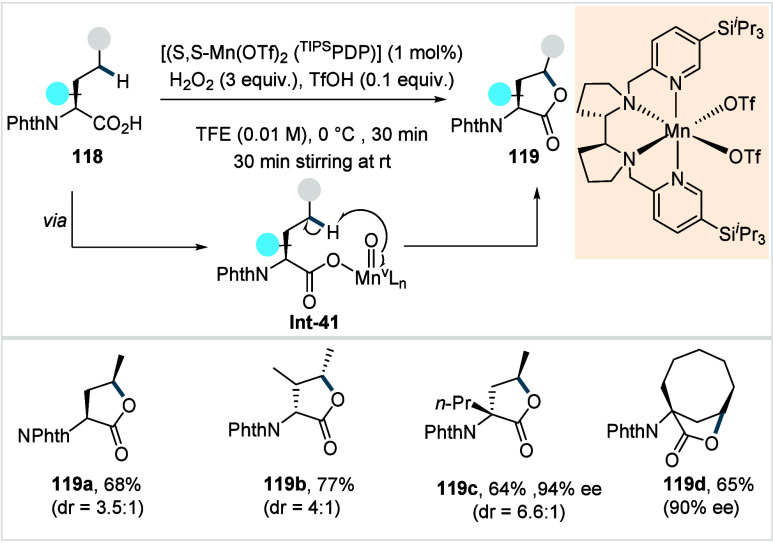
Costas et al. (**2021**): Stereoselective γ-Lactonization of α-Amino Acid Derivatives

The exclusive γ-selectivity was rationalized by the directing effect of the carboxylate group, which coordinates to the catalyst and orients the γ-C–H bond toward the high-valent metal-oxo species (**Int-41**), thereby enabling a 1,7-HAT pathway. A broad range of α-amino acids with aliphatic side chain (**119a**–**119b**) was transformed into the corresponding lactones in useful yields and with diastereoselectivities of 3.5:1 to 4.5:1. However, substrates bearing remote amide moieties on the side chain gave substantially dimished yields. The method was extended to the γ-C–H functionalization of achiral α,α-disubstituted α-amino acids, enabling a desymmetrization. While α,α-disubstituted linear amino acids furnished the respective lactones with high diastereoselectivities and excellent enantioselectivities for the major diastereomer (**119c**), α,α-disubstituted cyclic amino acids afforded single diastereomers with high enantioselectivities (**119d**).

In 2022, Bryliakov, Ottenbacher et al. reported a synthetic strategy for direct, regioselective conversion of fatty acids to γ-lactones via 2°-C­(sp^3^)–H functionalization.^162^ The optimization studies identified a Mn-catalyst with pyridine-groups bearing highly sterically demanding benzhydryl and strongly electron-donating methoxy-substituents (**C1**, Scheme 59). Using this catalyst and H_2_O_2_ as the oxidant (Conditions A), a variety of long-chain aliphatic and branched acids was transformed into the respective γ-lactones in good yields (**121a**–**121b**).

**Scheme 59:**
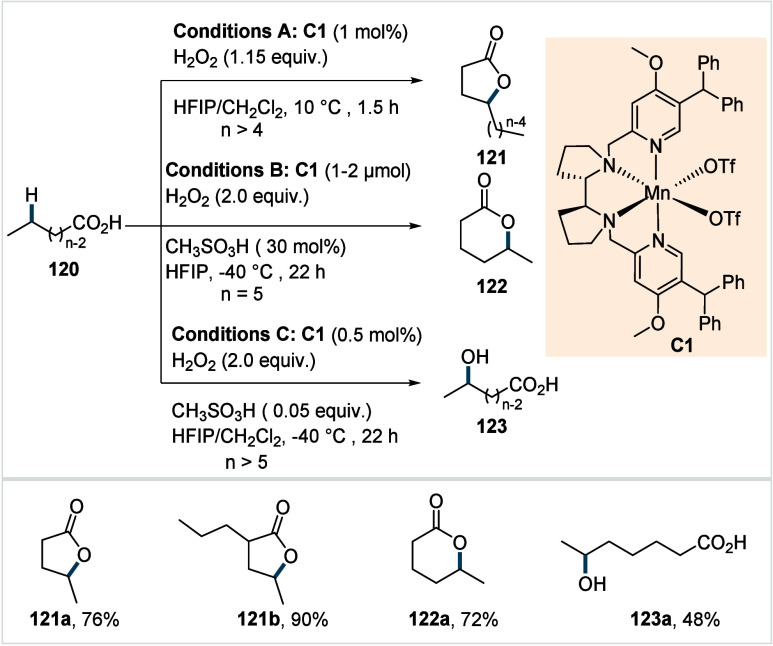
Bryliakov et al. (**2022**): Mn-Catalyzed Regioselective C­(sp^3^)–H Lactonization and Hydroxylation of Fatty Acids

A modest decrease in γ-lactone yield was noted with increasing the chain length. Interestingly, in the case of hexanoic acid, a preferential formation of the δ-lactone over the γ-lactone was observed under conditions employing a strong Bronsted acid additive at low temperature (**122a**, Conditions B). Furthermore, fatty acids containing seven or more carbon atoms were found to give (ω-1)-hydroxy products as the major products in moderate yields (**123a**), along with lactones as minor products. Recently, the authors reported an extended investigation of this method for the regio- and enantioselective γ-lactonization of a broad range of linear carboxylic acids. Mechanistic studies indicated that γ-lactonization proceeds via a carboxylic acid-directed intramolecular HAT mediated by a typical high-valent metal-oxo species, whereas the (ω-1)-hydroxylation process involves a nondirected intermolecular HAT.^163^

The Mn-based catalysts previously developed by the Costas group for the enantioselective γ-lactonization were limited to the use of adamantaneacetic acids and *N*-phthalimido protected α-amino acids as substrates. In 2023, Call, Bietti, and Costas et al. introduced a new class of sterically congested chiral Mn-based catalysts featuring *N*-ethylbenzimidazole moieties (**C2** and **C3**, Scheme 60), which enabled the desymmetrization of carboxylic acids via a highly enantioselective γ-lactonization of unactivated 2 °C–H bonds using H_2_O_2_.^164^

**Scheme 60:**
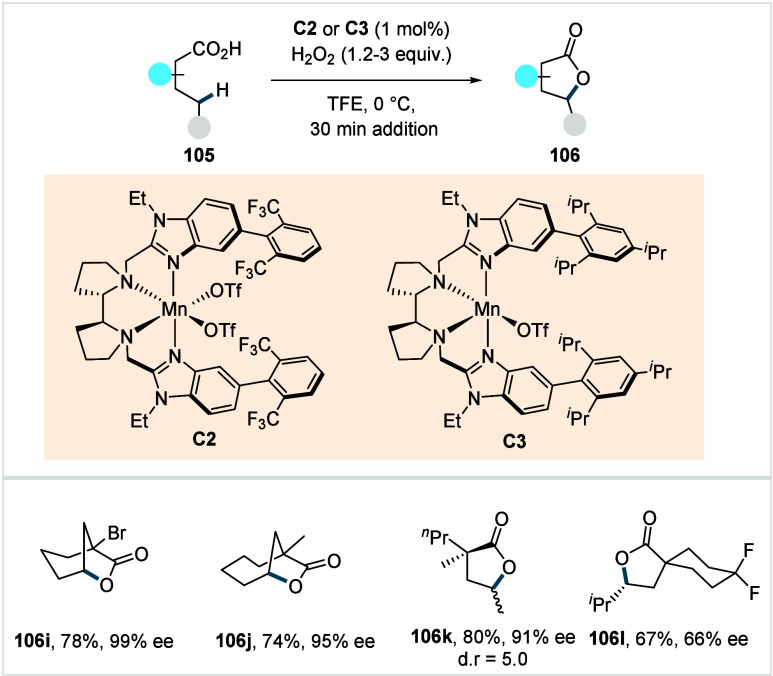
Costas et al. (**2023**): Mn-Catalyzed Enantioselective γ-Methylene-C–H Lactonization of Aliphatic Acids

Using catalyst **C2**, a series of α-substituted bicyclic lactones ranging from bicyclo [3.2.1] to bicyclo [5.2.1] frameworks was obtained in high yields and with excellent ee-values (**106i**–**106j**). However, α-unsubstituted cyclohexanecarboxylic acids exhibited poor reactivity using this protocol. α-Quaternary linear aliphatic acids afforded the respective lactones (**106k**) in higher yields and with higher ee-values for the major diastereomer compared to their α-nonquaternary analogs. Interestingly, the presence of electron-withdrawing groups within the cycloalkane ring suppressed endocyclic lactone formation, instead leading to spirocyclic γ-lactones through exclusive functionalization of exocyclic γ-C–H bonds of the alkyl side chain (**106l**).

Recently, the same group developed a catalytic system based on a manganese catalysts and hydrogen peroxide as the oxidant, enabling the desymmetrization of inherently less reactive, electron-poor C­(sp^3^)–H bonds in α,α-dialkylmalonic acid monoesters and malonic acids through a highly enantioselective directed C–H oxidation, yielding γ- and δ-lactones in good yields.^165^

The Yu group, in their previously discussed Cu^I^-catalyzed γ-lactonization protocol,^153^ also extended their method to the 2 °C–H functionalization of aliphatic acids (Scheme 61). While acyclic aliphatic acids delivered a mixture of γ- and δ-lactones, due to conformational constraints in the lactonization transition state, various cyclic substrate–derived lactones, including examples with a protected amine (**125a**) or derived from complex bioactive compounds (**125b**), were obtained with exclusive γ-selectivity and in good yields. Moreover, the method proved effective for the synthesis of bicyclo [3.2.1] and [2.2.1] lactones with diverse substitution patterns (**125c**–**125d**).

**Scheme 61:**
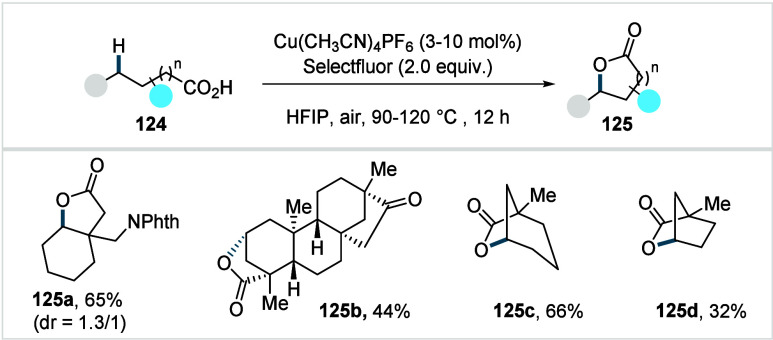
Yu et al. (**2024**): Copper Catalyzed 2° C­(sp^3^)–H Lactonization of Aliphatic Acids

### 3.4 Primary (1°)-C­(sp^3^)–H Functionalization

In 2022, Luis, Bietti, Costas and co-workers reported carboxylic acid directed γ-lactonization of unactivated 1 °C–H bonds (Scheme 62).^166^

**Scheme 62:**
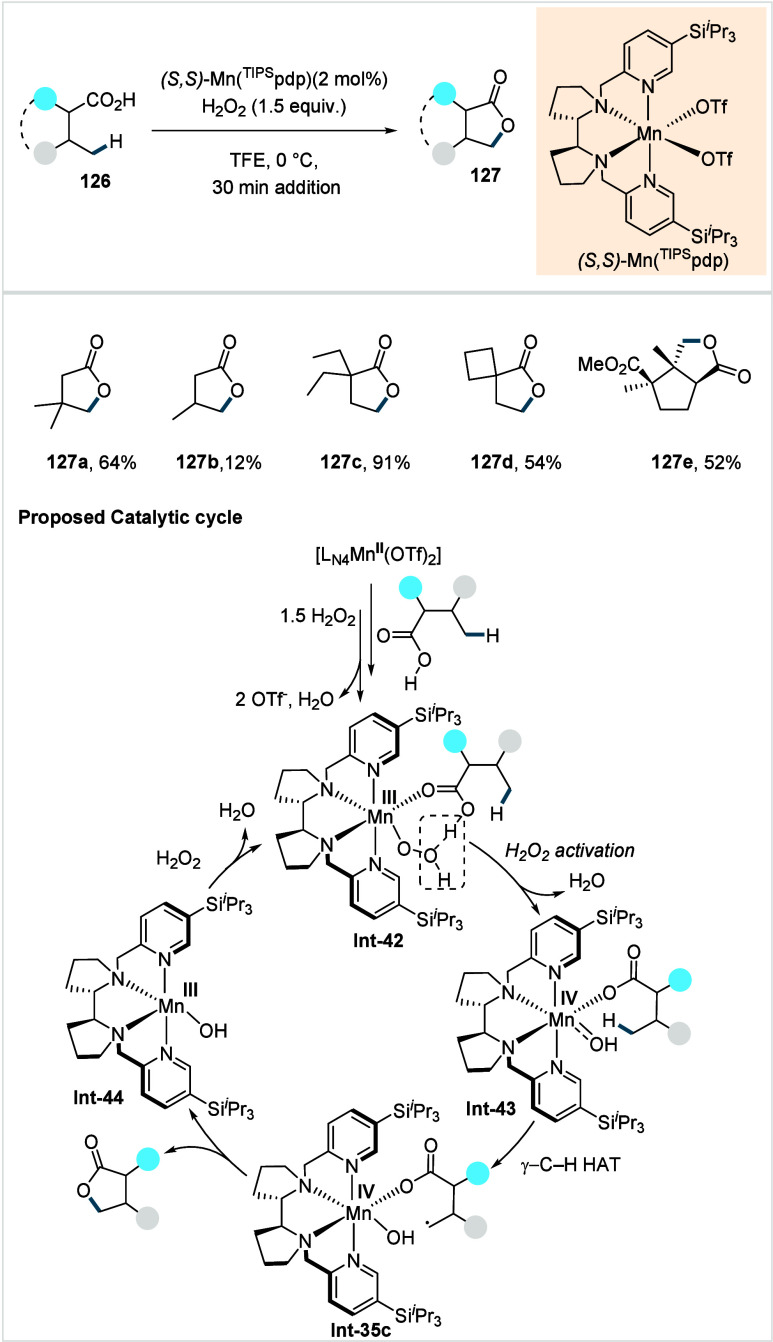
Costas et al. (**2022**): Manganese Catalyzed 1° C­(sp^3^)–H Lactonization of Aliphatic Acids

Using a Mn-catalyst with a ligand bearing trialkylsilyl-groups on the pyridine rings in combination with H_2_O_2,_ the method selectively functionalized primary C–H bonds over intrinsically more reactive secondary and tertiary sites. The chiral diamine backbone of the catalyst was found to be crucial for activity, as replacing bipyrrolidine with 1,2-cyclohexane diamine led to a substantial decrease in the yield. Substrate scope studies indicated that both the number of primary γ-C–H bonds and the presence or absence of a Thorpe-Ingold effect significantly influenced the efficiency of the lactonization process. For example, while 3,3 dimethyl butanoic acid, which possesses nine γ-methyl-C–H bonds, was converted to the corresponding lactone in a useful yield (**127a**), the removal of the methyl group(s) from the β-position drastically reduced the yields (**127b**). α-Quaternary acids afforded good yields of the respective lactones (**127c**) whereas α-nonquaternary acids displayed poor reactivity. It was observed that α-cyclopropyl- and cyclobutyl-substituted aliphatic acids underwent exclusive γ-methyl-C–H lactonization (**127d**). In contrast, aliphatic acids bearing five- or larger-membered cycloalkanes in the α-position yielded mixture of lactones arising from both γ-methyl and competing γ-methylene C–H functionalization, with the latter predominating. The protocol was also successfully applied to the late-stage functionalization of natural products, such as camphoric acid, affording the corresponding lactones in synthetically useful yields (**127e**). Based on the experimental and computational studies, the reaction was proposed to be initiated by the oxidation of the Mn^II^ precatalyst to a catalytically competent Mn^III^ species (**Int-42**), which is subsequently oxidized to a Mn^IV^-oxyl intermediate (**Int-43**) with the assistance of metal-bound carboxylate and H_2_O_2._ Next, **Int-43** undergoes an intramolecular 1,7-HAT from a primary γ-C–H bond to generate the carbon centered radical species **Int-35c**, which then readily undergoes lactonization. It should be noted that, unlike the mechanism discussed in Fe-catalyzed lactonization reaction (Scheme 55), the lactonization from **Int-35c** occurs via a carboxylate rebound mechanism, as confirmed by an ^18^O labeling study.

In the previously discussed enantioselective lactonization of secondary γ-C­(sp^3^)–H bonds (Scheme 60),^164^ the authors demonstrated that their catalyst could also functionalize nonactivated primary γ-C–H bonds (Scheme 63). A series of alkyl substituted cyclohexane carboxylic acids was efficiently converted to their corresponding fused-lactones in good yields and excellent enantioselectivities (**127f**).

**Scheme 63:**
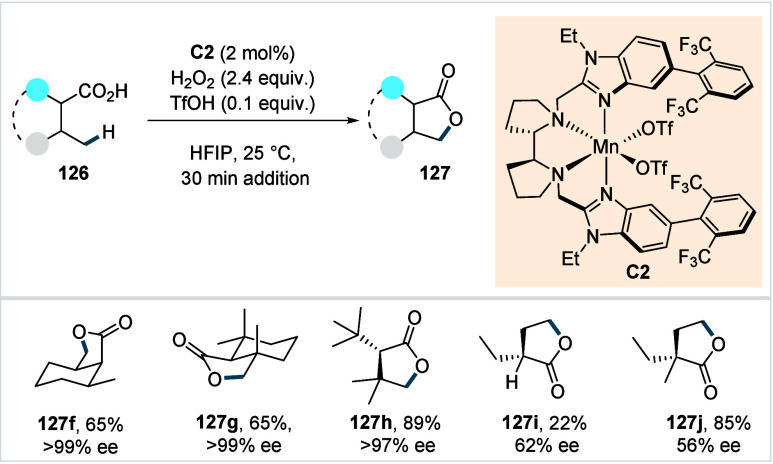
Costas et al. (**2023**): Manganese Catalyzed Enantioselective 1° C­(sp^3^)–H Lactonization of Aliphatic Acids

For substrates bearing multiple methyl groups both *cis* and *trans* to the carboxylic acid moiety, exclusive formation of *trans* lactone was observed (**127g**), indicating that the target methyl group need not be *cis* to the carboxylic acid. The protocol was successfully employed for the desymmetrization of α-alkyl substituted carboxylic acids, delivering the corresponding lactones in high yield and with an excellent enantioselectivity for the major diastereomer (**127h**). Removal of the β-methyl groups from substrate **126h** significantly reduced both yield and ee (**127i**). The introduction of an α-methyl group restored the desired reactivity (**127j**), highlighting the strong influence of Thorpe–Ingold effect on the reaction outcome.

In addition to the lactonization strategies described above, visible light-promoted methods,^167^ electrochemical methods,^168,169^ and hypervalent iodine-mediated approaches^170–172^ have also been employed for the synthesis of γ-lactones via C­(sp^3^)–H functionalization.

### 3.5 Catalyst Design in Carboxylic Acid Directed HAT-Based C­(sp^3^)–H Functionalization

The rational design and development of bioinspired 3d-metal-based catalysts has significantly advanced the selective functionalization of strong aliphatic C­(sp^3^)–H bonds via hydrogen atom transfer (HAT) mechanisms. The key principles guiding catalyst design are the geometry of the metal center and the nature of the active species involved in the HAT step. Typically, such processes require the participation of an electrophilic high-valent metal-oxo species to facilitate HAT, with such high-valent species derived from 3d-metals such as Fe and Mn adopting an octahedral geometry.

The discovery of such catalyst originates from studies on nondirected C­(sp^3^)–H oxidation processes.^173,174^ From this starting point, the development of directed C–H functionalization reactions using metal-bound oxo species demands careful control over both substrate and oxidant coordination. The catalyst must be able to accommodate the substrate and oxidant simultaneously at proximal sites, ensuring that the directed reaction proceeds efficiently and outcompetes nondirected intermolecular processes.^155^ Given that typical heme-based catalysts inherently provide open *trans* coordination sites, they are unsuitable for reactions that require simultaneous, proximal binding of substrate and oxidant. These considerations have driven the careful design of nonheme catalysts featuring appropriately designed tetradentate ligands, which occupy four coordination sites while ensuring two *cis* positions remain available for carboxylate and oxidant binding (Scheme 64). This arrangement enables efficient and selective directed C–H functionalization. The tetradentate ligands usually feature four strong σ-donating *N*-donors, which are known^173,174^ to be crucial for stabilizing high-valent metal-oxo species, while the rigid ligand backbone ensures steric shielding to control selectivity and can also impart stereochemical control.

**Scheme 64:**
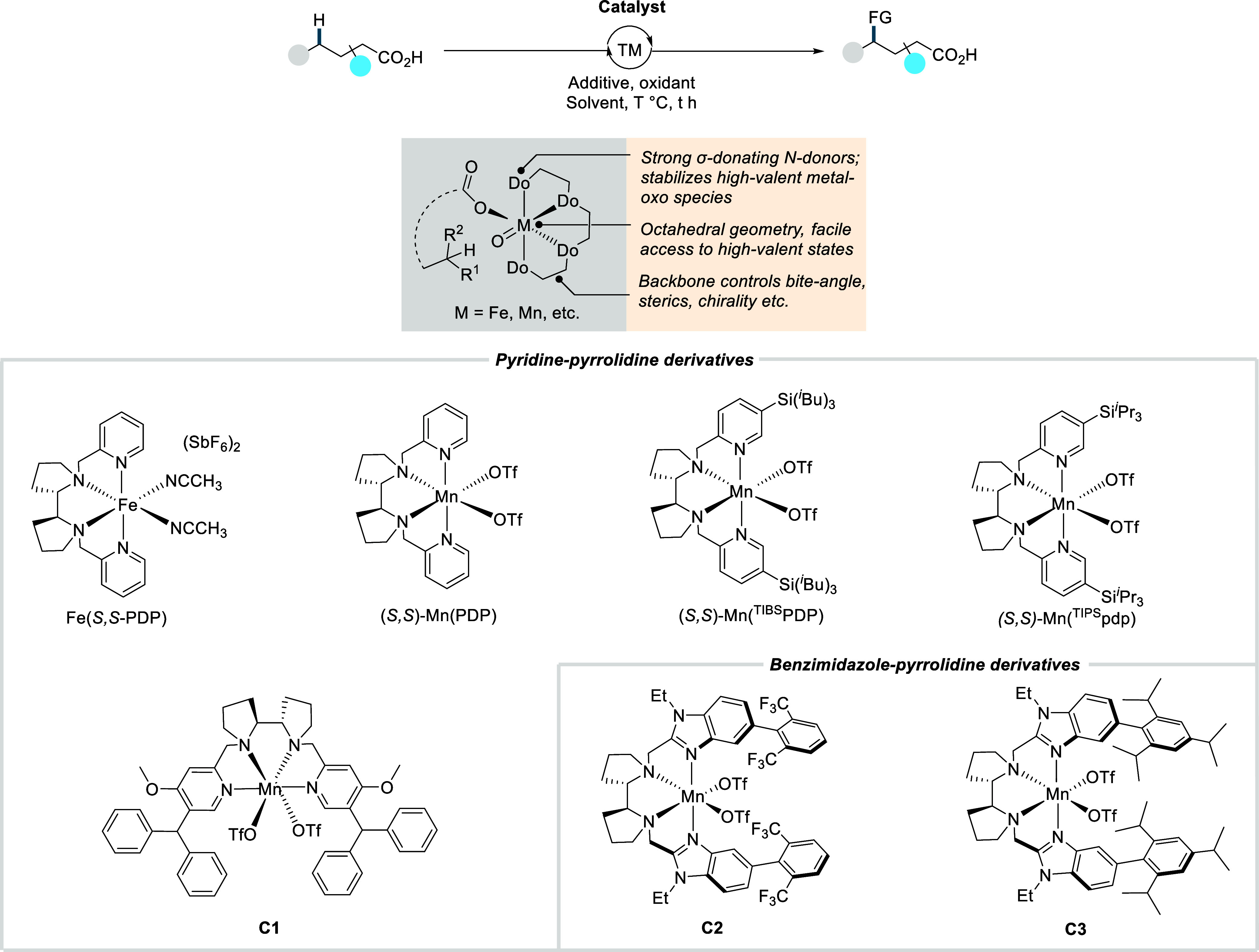
Catalyst Design in Carboxylic Acid Directed HAT-Based C­(sp^3^)–H Functionalization of Aliphatic Acids

Unlike Pd^II^ precatalysts, which can form active complexes in situ with mono- or bidentate ligands due to facile ligand exchange in square-planar geometries,^175,176^ 3d-metal-based catalysts are typically employed as presynthesized active catalyst. Pioneering work on carboxylate-directed nonheme Fe­(PDP)-catalyzed C­(sp^3^)–H oxidations laid the foundation for this field, while a variety of Mn-based catalysts featuring pyridine–pyrrolidine and benzimidazole–pyrrolidine motifs later enabled complementary reactivity patterns. Specifically, while Fe-based catalysts are primarily effective for the functionalization of tertiary (3°) C–H bonds, Mn-based catalysts have expanded the scope to secondary (2°) and primary (1°) C–H bonds.

## 4 C–H Functionalization of Aliphatic Acids Enabled by Noncovalent Interactions

### 4.1 Chemoenzymatic C­(sp^3^)–H Functionalization

Metalloenzymes have emerged as highly efficient catalysts for site-selective C­(sp^3^)–H oxidation reactions. These reactions proceed via a hydrogen-atom transfer (HAT) process involving high-valent metal-oxo species, which are formed upon the reductive activation of O_2_ or H_2_O_2_ at the metal centers. The precise orientation of substrates is guided by weak-interactions with the amino acid residues in the metalloenzyme, positioning specific C–H bonds close to the reactive oxidant while shielding others and thereby enabling exquisite site-selectivity. In 2019, Flitsch and co-workers reported the use of a wild-type cytochrome P450 monooxygenase to achieve C5 hydroxylation of decanoic acid, which was subsequently transformed into a δ-lactone with high enantioselectivity (90% ee) under acidic conditions.^177^

In 2021, Wang et al. described a highly regio- and enantioselective oxyfunctionalization of unactivated C­(sp^3^)–H bonds of fatty acids using P450_BSβ_-L78A/Q85A/V170I/G290I, a quadruple variant of the P450_BSβ_ hydroxylase enzyme (Scheme 65).^178^

**Scheme 65:**
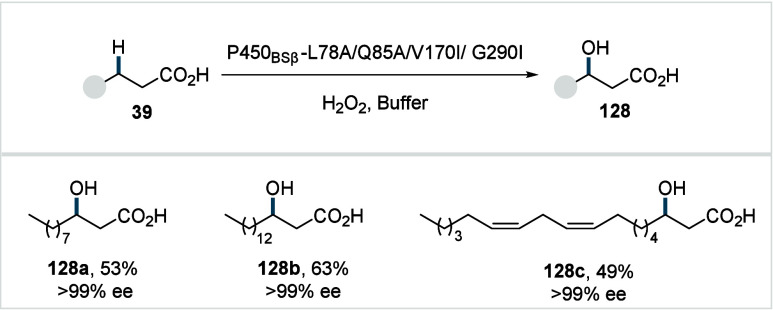
Wang et al. (**2021**): Enzymatic C­(sp^3^)–H Oxyfunctionalization of Aliphatic Acids

This enzymatic transformation enabled the β-C­(sp^3^)–H hydroxylation of C11–C18 saturated and naturally derived unsaturated fatty acids (**128a**–**128c**) in good yields and excellent enantioselectivities (>99%). The authors highlighted the synthetic utility of the enantiopure β-hydroxylated products by transforming them into various β-functionalized derivaties, including β-lactones and β-lactams. However, fatty acids with chain lengths below 10 carbon atoms or containing aryl or heteroaryl groups were found to be incompatible with this protocol.

To address these limitations, the authors subsequently developed an improved protocol for the enantioselective β-C­(sp^3^)–H hydroxylation of aliphatic carboxylic acids through directed evolution of P450_BSβ_ hydroxylase (Scheme 66).^179^ Using the variant P450_BSβ_-L78I/Q85H/G290I and inexpensive H_2_O_2_, the reaction proceeded under mild conditions and with a high site-selectivity for the β- over the α-position (β/α > 20).

**Scheme 66:**
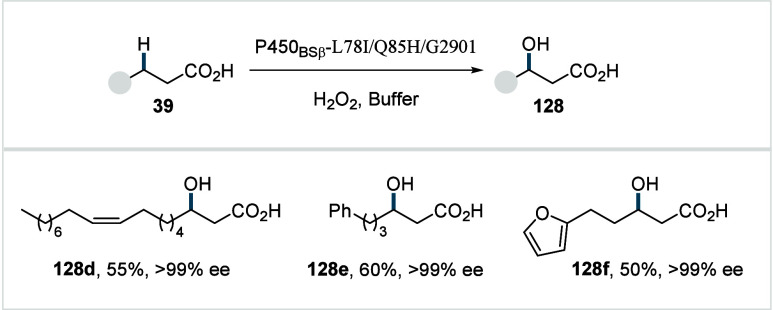
Wang et al. (**2022**): Enzymatic Enantioselective β-C­(sp^3^)–H Hydroxylation of Aliphatic Carboxylic Acids

A broad range of hyroxy fatty acids (HFAs) with 6–20 carbon atoms, including substrates bearing alkene (**128d**) and electronically different arene (**128e**) groups was well-tolerated, affording the corresponding β-hydroxy acids in good yields and excellent enantioselectivities. Aliphatic acids containing heteroaromatic moieties (**128f**) furnished moderate yields while retaining the excellent stereoselectivity.

In 2024, Liao, Bai, He, and Wang et al. detailed an enantioselective γ-lactonization of aliphatic carboxylic acids using a peroxygenase P450_BSβ_ variant (Scheme 67).^180^ Through directed evolution, a quadruple mutant, P450_BSβ_-L78G/Q85F/F173S/G290I, was identified, which substantially shifted the site-selectivity of the hydroxylation from the undesired β- to the desired γ-position. Subsequent acidic treatment of the hydroxy acids afforded the corresponding γ-lactones. A diverse library of γ-lactones, including examples derived from fatty acids (**130a**) and bearing functional groups such as alkenes, arenes (**130b**), and heteroarenes (**130c**–**130d**) was accessed in good yields and with excellent enantioselectivities. As a remaining limitation, aliphatic acids featuring a thiophene or furan motif provided low yields.

**Scheme 67:**
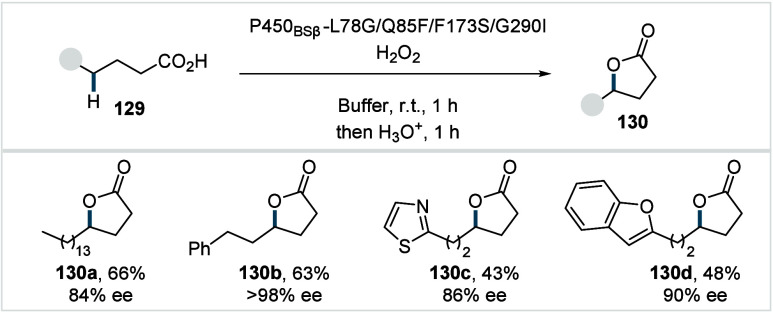
Liao/He/Bai/Wang et al. (**2024**): Enzymatic Enantioselective γ-Lactonization of Aliphatic Carboxylic Acids

In addition to the above-mentioned enzymatic transformations, α-ketoglutarate-dependent dioxygenase (Fe/αKG) enzymes have enabled the oxidative modification of amino acids, including hydroxylations, halogenations, and cyclizations (Scheme 68).

**Scheme 68:**
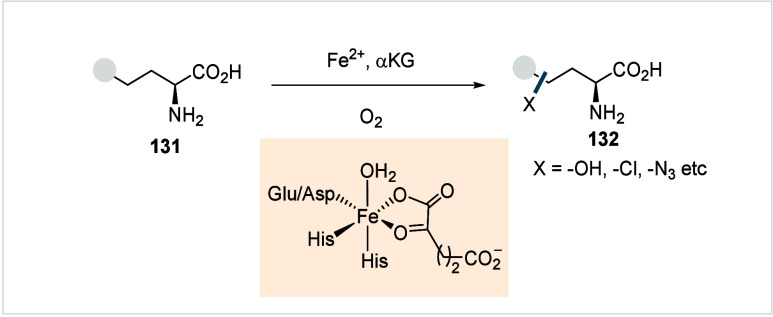
Enzymatic C­(sp^3^)–H Functionalization of Amino Acids

Since these methods are not exclusively directed by the carboxylate moiety but require the presence of an amino acid motif, they are not discussed in detail here, but have been summarized in detail in the literature.^181,182^

### 4.2 Supramolecular Recognition-Enabled C­(sp^3^)–H Functionalization

In 2002, Breslow and co-workers developed an artificial metalloenzyme consisting of a manganese-porphyrine decorated with four β-cyclodextrin (β-CD) units (**C4**) that enabled the selective benzylic C­(sp^3^)–H hydroxylation of a dihydrostilbene substrate (**133**) using an excess of iodosobenzene as oxidant (Scheme 69).^183^ The four β-CD rings enhanced the water solubility of the catalysts and facilitated encapsulation of the substrate’s hydrophobic moiety. This substrate-catalyst interaction was crucial in enabling the precise orientation of the target C­(sp^3^)–H bond relative to the reactive metal center. To ensure the cofacial alignment of the metal-bound oxygen and the substrate, pyridine was employed to block the opposing face of the catalyst.

**Scheme 69:**
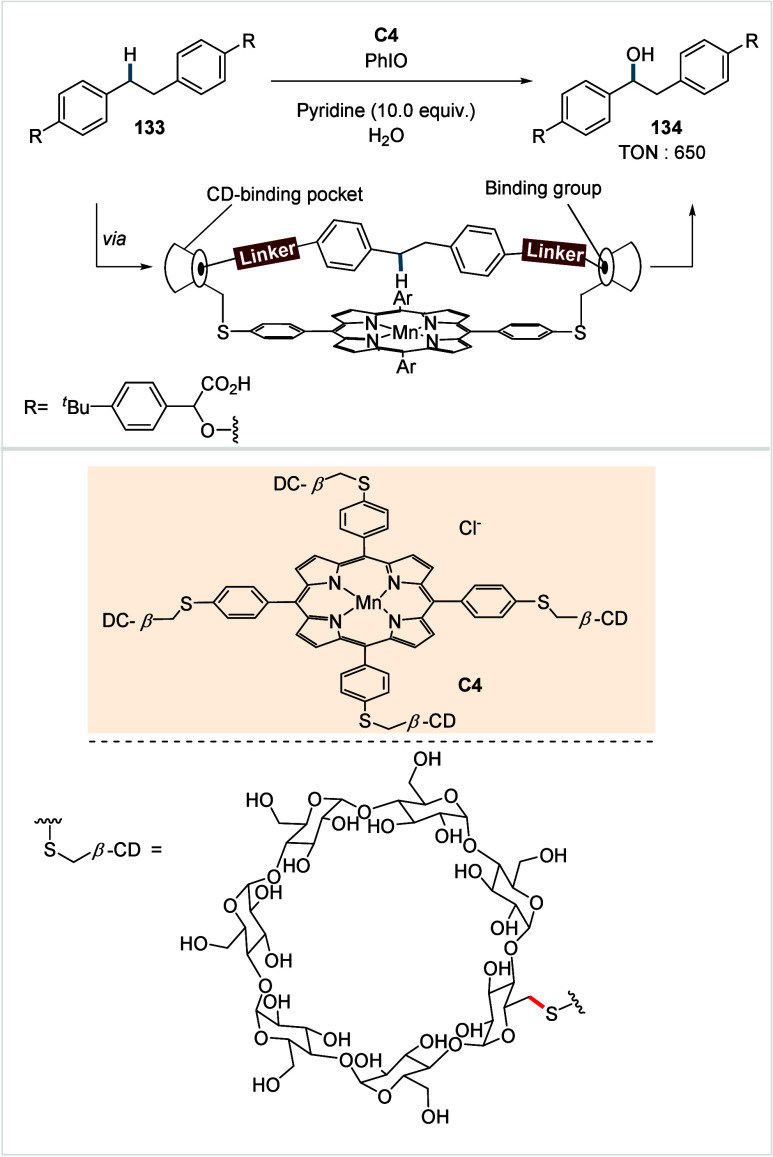
Catalytic C­(sp^3^)–H Oxidation Using Artificial Cytochrome P-450 Enzymes

The catalyst **C4** substantially outperformed its Fe analog. Interestingly, when a modified variant of **C4** was applied to lithocholenic acid, containing both a reactive hydroxyl and a double bond, the oxidation occurred exclusively at the saturated carbon centers, yielding a mixture of keto and diol products, while the double bond remained protected within the cyclodextrin cavity.

In 2006, Crabtree and Brudvig et al. developed a dimanganese-based catalyst, comprising a Mn­(μ-O)_2_Mn core and a Kemp’s triacid-derived ligand **C5**, capable of inducing a remote C­(sp^3^)–H oxidation of ibuprofen (Scheme 70).^184^ The protocol employed tetrabutylammonium oxone as the oxidant and proceeded via a high-valent metal-oxo species to give the remote benzylic C­(sp^3^)–H hydroxylated product (**136**) in 69% yield. The terpyridine ligand was proposed to stabilize the high-valent Mn­(oxo) species. Mechanistic studies indicated that the regioselectivity arises from a noncovalent molecular recognition through reversible H-bonding between the carboxylic acid group of the catalyst (**C5**) and that of the substrate.^185^ The phenelyne linker and the Kemp’s triacid unit form a U-turn motif which precisely positions the – COOH group for an effective molecular recognition that was found to be decisive for the success of the protocol.

**Scheme 70:**
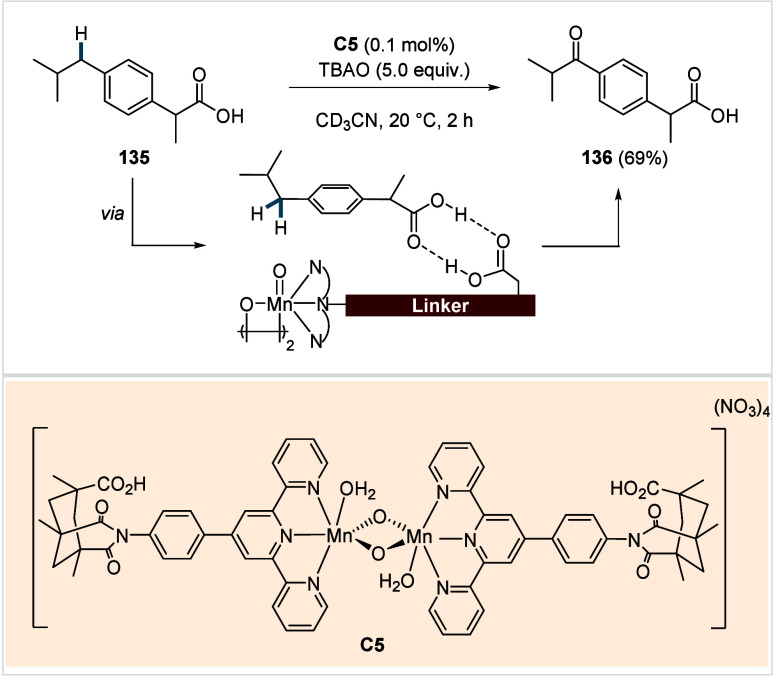
Selective C­(sp^3^)–H Oxidation of Ibuprofen Using Molecular Recognition

## 5 Applications of C(sp^3^)–H Functionalization Reactions in Complex Molecule Synthesis

In modern synthetic chemistry, achieving short and scalable syntheses of natural products and bioactive compounds remains a major challenge. Traditional target-oriented strategies typically rely on two complementary reactive functional groups to construct new C–C or C–X bonds, often requiring preinstallation of these groups thus increasing the overall step count and complexity. Direct functionalization of inert C­(sp^3^)–H bonds offers an intrinsically attractive alternative, improving step-, atom-, and redox-economy by allowing one or both functional groups to be replaced by C–H bonds.^186,187^ This approach is particularly advantageous in late-stage functionalization, where selective activation of a specific C–H bond among multiple chemically similar sites can streamline synthesis and enable rapid molecular diversification. Carboxylic acids frequently arise as intermediates in traditional synthetic sequences, making them particularly attractive as directing groups for C­(sp^3^)–H functionalization. Over the past decades, methods for carboxylic acid directed C­(sp^3^)–H functionalization have expanded significantly, providing access to diverse C–C and C–X bond-forming transformations. Various activation modes have enabled orthogonal reactivity profiles with respect to regioselectivity and functional group tolerance. Consequently, carboxylic acid–directed C­(sp^3^)–H functionalization has found increasing application in the efficient synthesis of complex natural products and bioactive compounds. This chapter highlights such applications in total and formal syntheses, emphasizing their strategic value in modern synthetic design.

### 5.1 Application of C­(sp^3^)–H Activation Reactions in Total Synthesis and Formal Synthesis of Natural Products and Bioactive Compounds

#### 5.1.1 Applications of Carbon–Carbon Bond Forming Reactions in Total Synthesis

In 2017, the Zhao group reported the synthetic application of their β-C­(sp^3^)–H arylation methodology in a concise synthesis of iopanoic acid (**3j**), an iodine-containing radiocontrast agent used in cholecystography (Scheme 71).^57^ Starting from commercially available 2-methylbutanoic acid (**1r**), the target molecule was assembled in three steps. The sequence began with a β-arylation using **4a** to afford **3r** in 69% yield, which was subsequently converted to iopanoic acid (**3j**) through phthalimide deprotection followed by iodination.

**Scheme 71:**
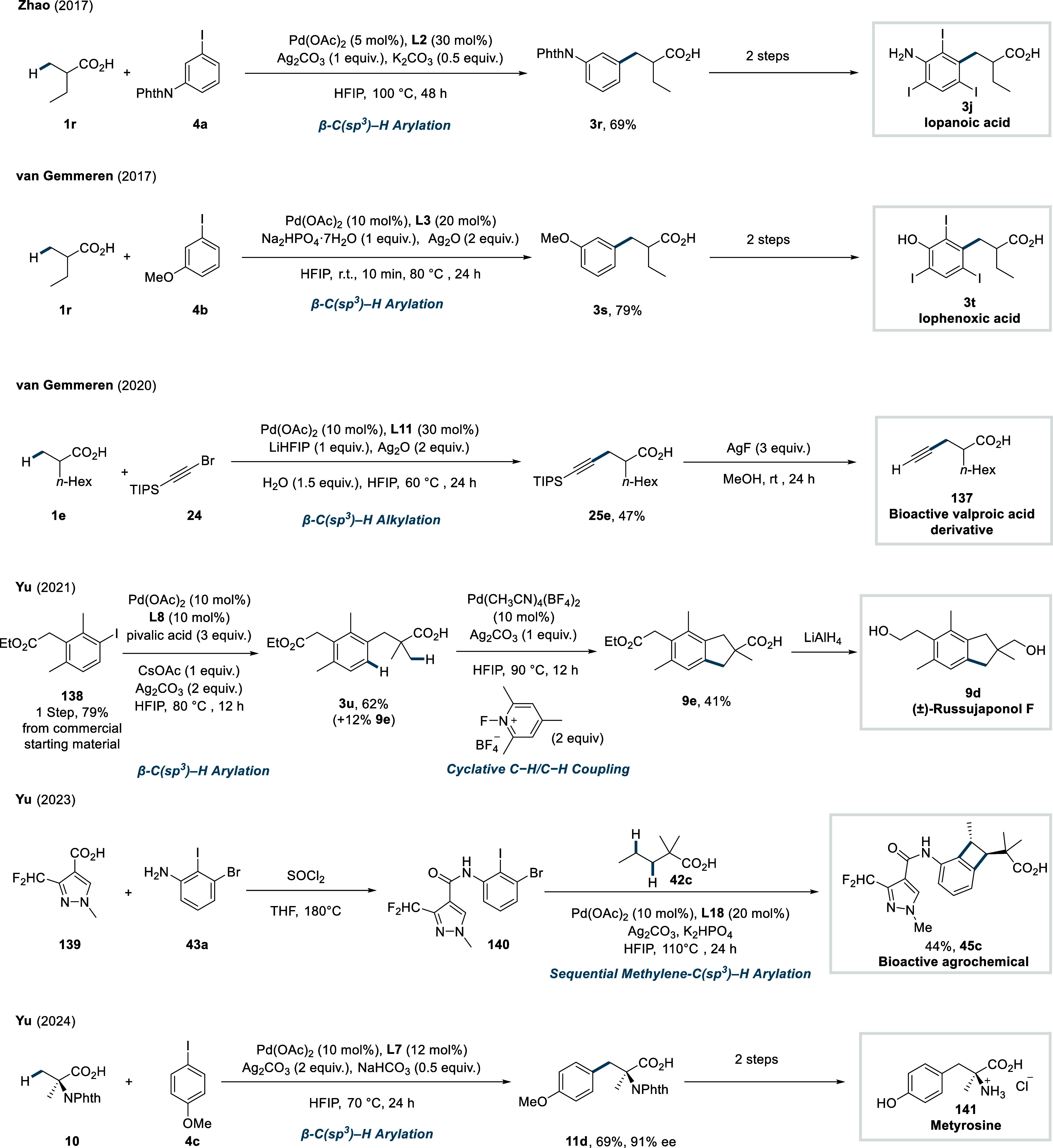
Applications of C–C Bond Forming Reactions in Natural Product Synthesis

In the same year, the van Gemmeren group applied their β-C­(sp^3^)–H arylation protocol to the synthesis of iophenoxic acid (**3t**), a compound used in wildlife bait acceptance studies and formerly marketed as Teridax for cholecystography.^61^ Starting from 2-methylbutanoic acid (**1r**), the three-step sequence commenced with β-arylation using **4b** in 79% yield to afford **3s**, followed by demethylation and triple iodination to furnish the target molecule.

In 2020, van Gemmeren et al. demonstrated the synthesis of a bioactive valproic acid derivative (**137**) using their β-C­(sp^3^)–H alkynylation protocol.^74^ The two-step sequence involved β-alkynylation of commercially available 2-methylheptanoic acid (**1e**) to furnish **25e** in 47% yield, followed by TIPS deprotection to afford **137**.

In 2021, the Yu group showcased the utility of their cyclative C–H/C–H coupling strategy in a four-step total synthesis of (±)-russujaponol (**9d**).^66^ The authors utilized aryl iodide **138** obtained from commercially available 2,6-dimethylphenylacetic acid in a single step as a starting point with a 79% yield, which was converted to precursor **3u** in 62% yield through a β-arylation of pivalic acid, giving **9e** as a side product in 12% yield. Subsequent cyclative C–H/C–C coupling of **3u** with a [F^+^] oxidant afforded **9e** in 41% yield, and global reduction with LiAlH_4_ furnished the target molecule.

In 2023, the Yu group applied their [2 + 2] benzannulation strategy to the synthesis of benzocyclobutyl amide (**45c**), a patented bioactive molecule used in crop protection, through a concise two-step sequence.^93^ The authors first prepared the *N*-dihaloaryl amide (**140**) via amide coupling between **139** and **43a**. The resulting intermediate subsequently underwent [2 + 2] benzannulation with **42c** to furnish the target compound (**45c**) in 44% yield.

In 2024, the Yu group reported a concise synthesis of metyrosine (**141**), a therapeutic agent used to manage hypertension, particularly in patients with adrenal tumors.^69^ In this three-step sequence, an enantioselective β-arylation of phthalimide-protected α-aminoisobutyric acid (**10**) with **4c** furnished the β-arylated product **11d** in 69% yield. Subsequent demethylation and phthalimide deprotection delivered the target molecule.

Yu and co-workers, in their study on transannular γ-C­(sp^3^)–H arylation, reported that a simple α-tertiary cyclopentane carboxylic acid (**41g**, Scheme 72) substrate provided one-step access to compound **46g** in 38% yield, a known AKR1C1 and AKR1C3 inhibitor.^96^

**Scheme 72:**
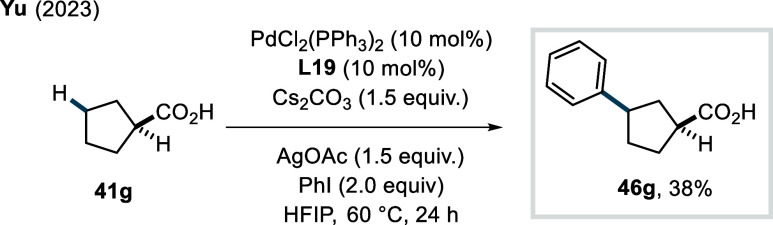
Applications of C–C Bond Forming Reactions in One-Step Synthesis of Bioactive Compounds

#### 5.1.2 Applications of Carbon–Oxygen Bond Forming Reactions in Total Synthesis

In 2009, White and co-workers reported the total synthesis of 6-deoxyerythronolide (**143**), the aglycone precursor to the erythromycin class of antibiotics, featuring a late-stage allylic C–H oxidation strategy (Scheme 73).^124^ In this approach, macrolactonization precursor **79d** was assembled in 18 steps from **142**. The macrolactonization of **79d** was then enabled by a Pd^II^/bis-sulfoxide catalyst system, furnishing macrocycle **80d** in an overall yield of 56% after two rounds of starting-material recovery and recycling. Elaboration of **80d** to the target molecule was accomplished in 3 further steps delivering 6-deoxyerythronolide in 7.8% overall yield over 22 steps.

**Scheme 73:**
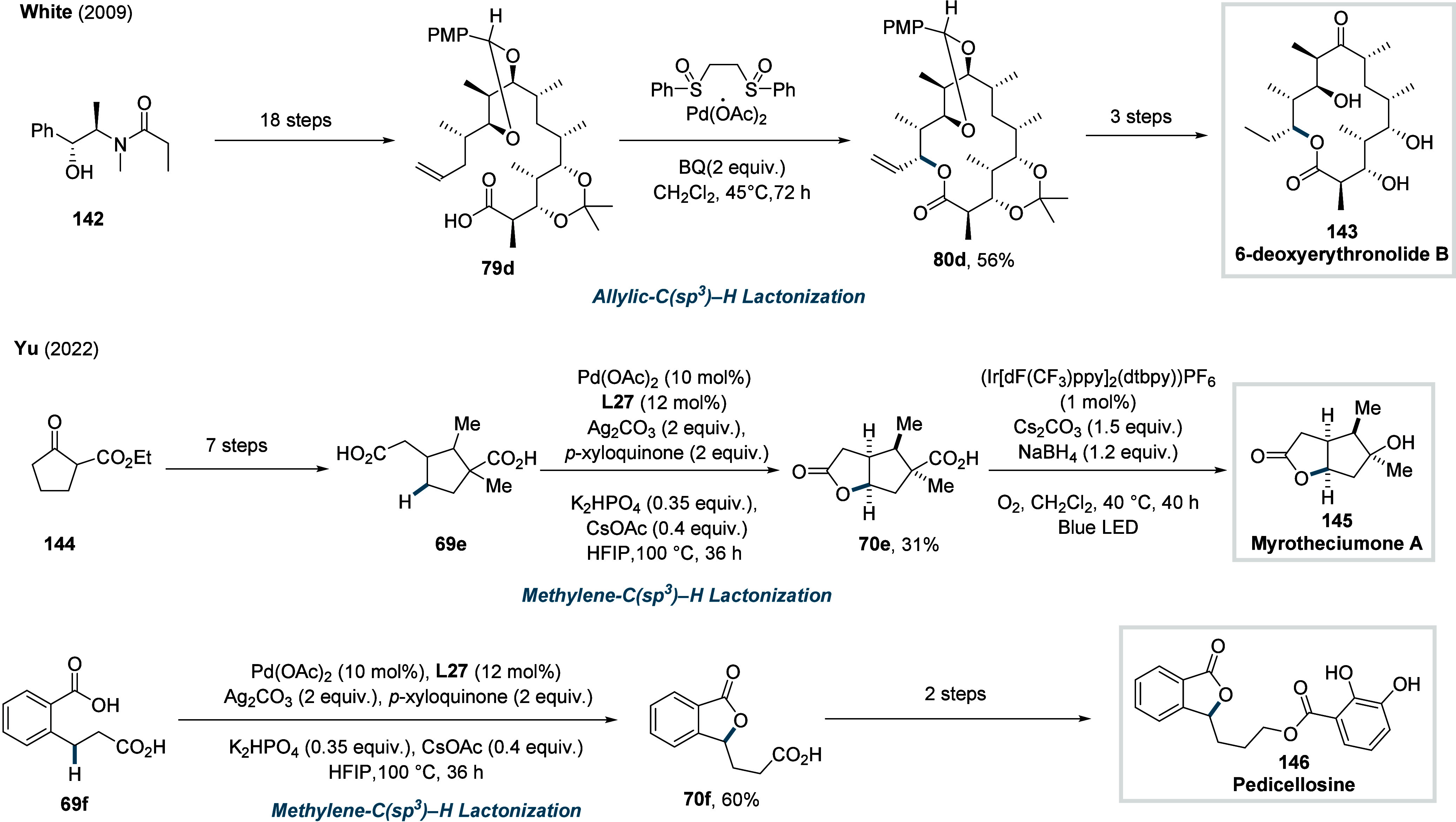
Applications of C–O Bond Forming Reactions in Natural Product Synthesis

Myrotheciumone A (**145**), a cytotoxic lactone isolated from the fungus *Ajuga decumbens*, possesses a bicyclic 5,5-fused framework. In 2022, Yu et al. showcased the utility of their methylene C­(sp^3^)–H lactonization of dicarboxylic acids through a nine-step total synthesis of this natural product, commencing from **144**.^118^ The dicarboxylic acid **69e** was prepared in seven steps from **144**. Subjecting **69e** to the standard lactonization conditions furnished the bicyclic 5,5-fused lactone **70e** in 31% yield. Subsequent photocatalytic decarboxylative hydroxylation of **70e** delivered myrotheciumone, thereby completing the synthesis in nine steps overall.

In the same study, the authors further demonstrated the versatility of their strategy through a concise total synthesis of pedicellosine, a phthalide isolated from the leaves of *Gentiana pedicellata* bearing a benzo-fused lactone core (**146**). The carboxylic acid directed γ-C­(sp^3^)–H lactonization of **69f** delivered the benzo-fused lactone **70f** in 60% yield. Subsequent reduction of the carboxylic acid and esterification completed the synthesis in only three steps from **69f**.

In 2025, Yu and co-workers demonstrated the synthetic potential of their triple C–H functionalization strategy through its application to the total synthesis of auxofuran (**147**), a bioactive natural product of interest for pharmaceutical research due to its fungal growth–stimulating properties.^136^ The sequence commenced with alkylation of commercially available pentanoic acid (**39a**, Scheme 74), furnishing intermediate **103e**. Treatment of **103e** under the conditions developed for butenolide formation enabled its transformation into intermediate **104e** with 42% yield. From this intermediate, a series of ring-closing steps followed by selective oxidation ultimately delivered the target molecule in an overall 29% yield over six steps.

**Scheme 74:**
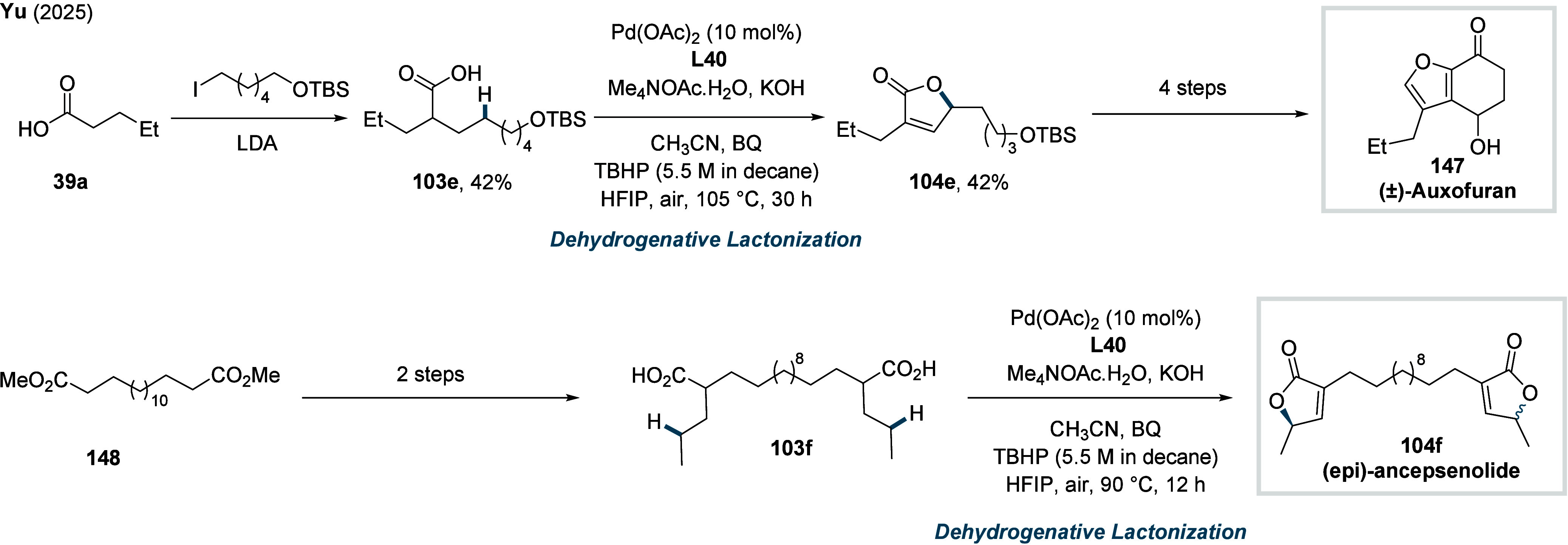
Applications of Miscellaneous Reactions in Natural Product Synthesis

In the same study, the authors also achieved the synthesis of ancepsenolide (**104f**), a marine-derived metabolite exhibiting diverse biological activities, including anticancer and antimalarial properties. Starting from **148**, LDA-mediated dialkylation followed by hydrolysis furnished the diacid precursor **103f**. Application of the standard lactonization protocol then delivered the target molecule (**104f**) in 40% overall yield over three steps.

Incrustoporin, featuring a butenolide ring, exhibits antifungal activity. Yu and co-workers, in their triple C–H functionalization protocol, demonstrated that this strategy enables one-step access to incrustoporin from the commercially available carboxylic acid (**103g**, Scheme 75) precursor in 42% yield.^136^

**Scheme 75:**
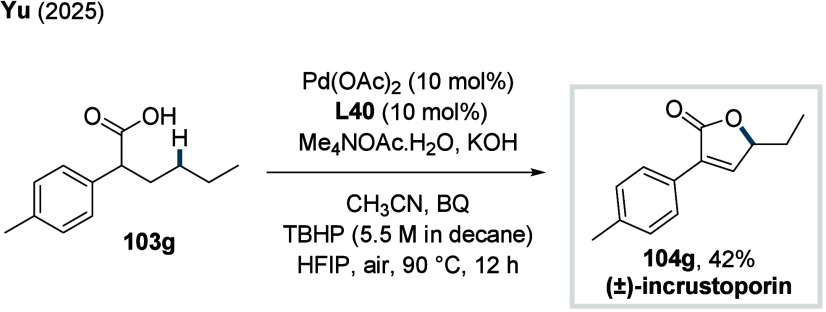
One-Step Synthesis of Natural Products Using C–O Bond Forming Reactions

#### 5.1.3 Applications of Carbon–Carbon Bond Forming Reactions in Formal Synthesis

In 2017, Zhao et al. employed their β-arylation methodology to access compound **149**, a key intermediate in the synthesis of L-thyroxine (**150**, Scheme 76).^57^ Their approach involved β-arylation of **5c** with 4-methoxy iodobenzene (**4c**), which furnished **6c** in 81% yield. Compound **6c** was subsequently converted into **149** over three steps, involving demethylation, phthalimide cleavage, and iodination.

**Scheme 76:**
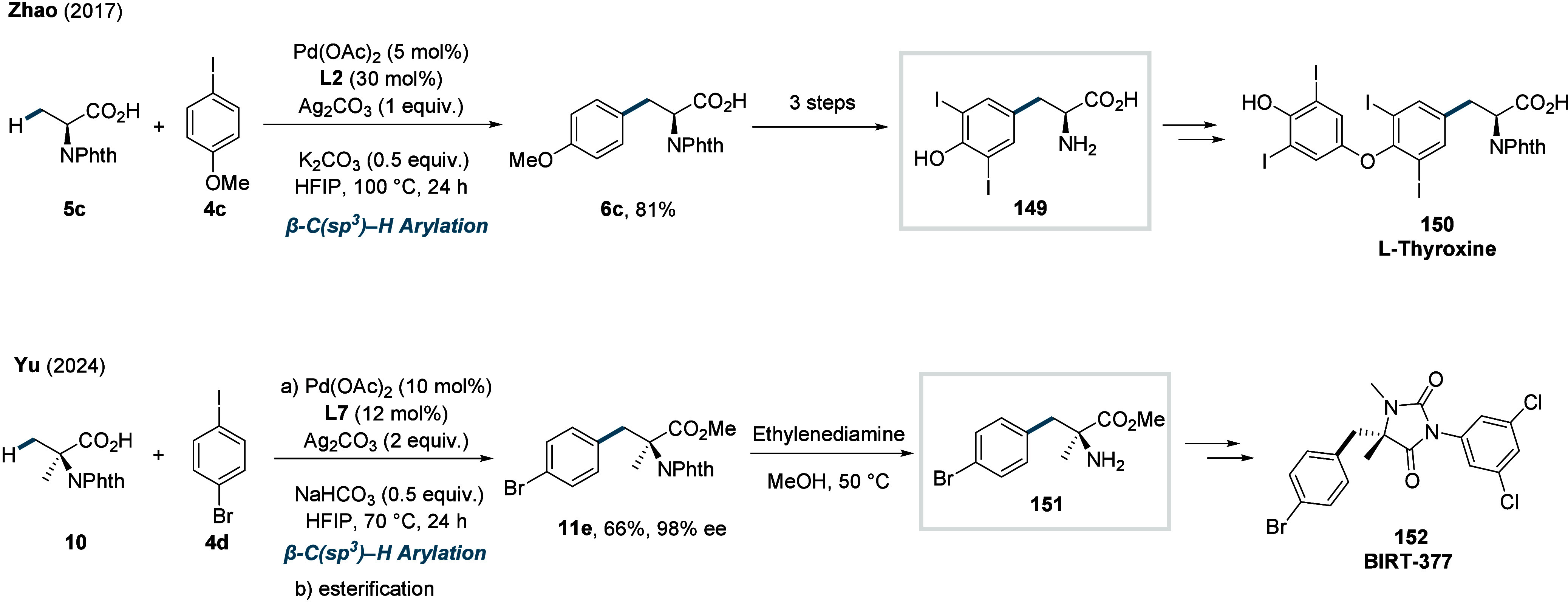
Applications of C–C Bond Forming Reactions in Formal Synthesis

In 2024, the Yu group employed their enantioselective β-arylation protocol to access compound **151**, a key intermediate in the synthesis of BIRT-377 (**152**).^69^ The synthesis commenced with β-arylation of **10** using **4d** to furnish the arylated acid, which was isolated as the methyl ester **11e** in 66% yield. Subsequent phthalimide deprotection delivered **151**.

In 2023, Yu and co-workers disclosed the synthesis of a series of histone deacetylase (HDAC) inhibitor precursors via their transannular γ-C­(sp^3^)–H arylation protocol (Scheme 77).^96^ Subjecting α-aryl cyclopentane carboxylic acid **41a** to the standard conditions delivered the corresponding γ-arylated products (**46h**–**46k**) in good yields in a single step upon coupling with the respective aryl iodides. Notably, In the following year, the authors obtained **46j** in 45% yield and 93% ee using their enantioselective protocol.^98^

**Scheme 77:**
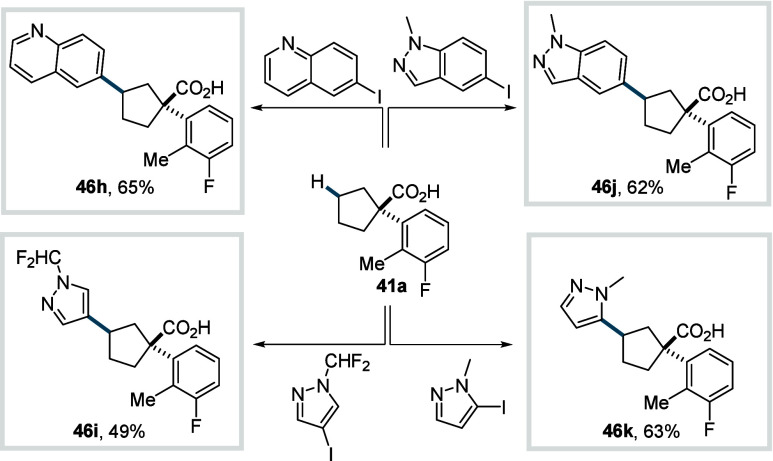
Applications of C–C Bond-Forming Reactions in One-Step Formal Synthesis

#### 5.1.4 Applications of Carbon–Oxygen and Miscellaneous Bond-Forming Reactions in Formal Synthesis

In 2023, Maiti et al. demonstrated the synthetic utility of their lactonization protocol through the formal synthesis of several natural products and bioactive compounds (Scheme 78).^120^ Intermediates **71e**–**71g** serve as key building blocks in the syntheses of capnellene (**155**), a marine sesquiterpene with anti-inflammatory activity; trichodiene (**156**), a biologically active sesquiterpene hydrocarbon; and sesquiterpenes such as seychellene (**157**). The syntheses commenced from cyclopentanone (**153**) or 4-methylcyclohexanone (**154**), which were converted into the corresponding cyclohexaneacetic acid derivatives **47e**–**47f** in three steps. Subsequent exposure to the standard lactonization conditions furnished the bicyclic lactone intermediates **71e** (62%) and **71f** (65%). Hydrogenation of **71f** provided **71g**, the synthetic intermediate en route to seychellene.

**Scheme 78:**
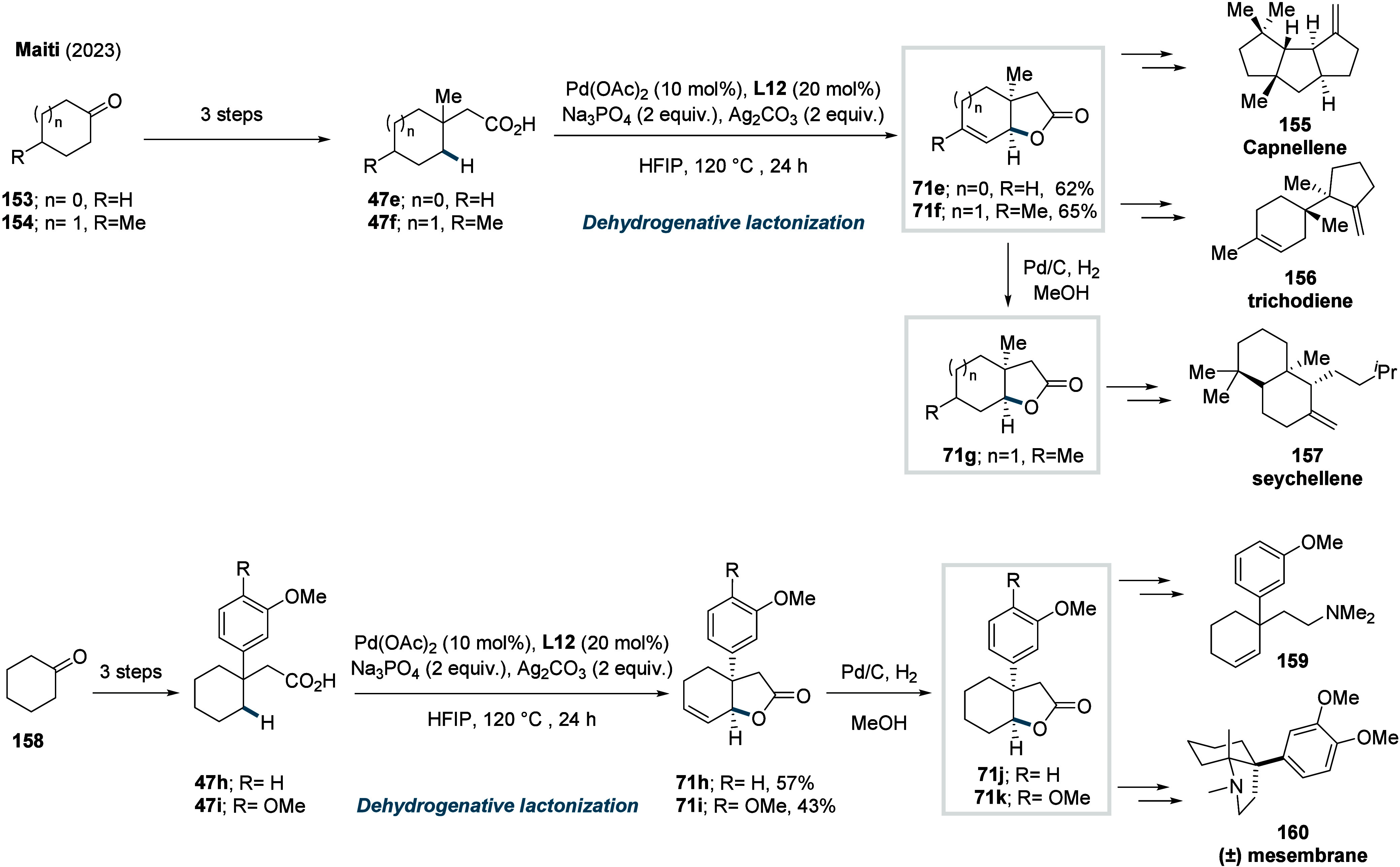
Applications of C–O Bond Forming Reactions in Formal Synthesis

Additionally, the authors identified methoxyaryl-substituted [6,5]-bicyclic lactones **71j**–**71k** as key intermediates for the total synthesis of the analgesic drug **159** and mesembrane (**160**), a natural alkaloid of the Amaryllidaceae family exhibiting diverse biological activities. Starting from cyclohexanone (**158**), and employing lactonization as the key step, intermediates **71h**–**71i** were prepared in four steps, with the lactonization proceeding in 57% and 43% yield, respectively. Subsequent hydrogenation in the presence of Pd/C furnished the target intermediates in five steps overall.

In 2025, Yu et al. reported a formal synthesis of racemic stemoamide (**162**) using their methylene C­(sp^3^)–H lactamization protocol (Scheme 79). The synthesis commenced with the formation of γ-lactam **86c** from **84c** in 57% yield under the standard lactamization conditions.^129^ The precursor **86c** was then converted into the desired intermediate **161** over four steps, involving tosyl group deprotection, an Ireland–Claisen rearrangement, a combined ring-closing metathesis/hydrogenation step, and a photoredox decarboxylative oxygenation.

**Scheme 79:**
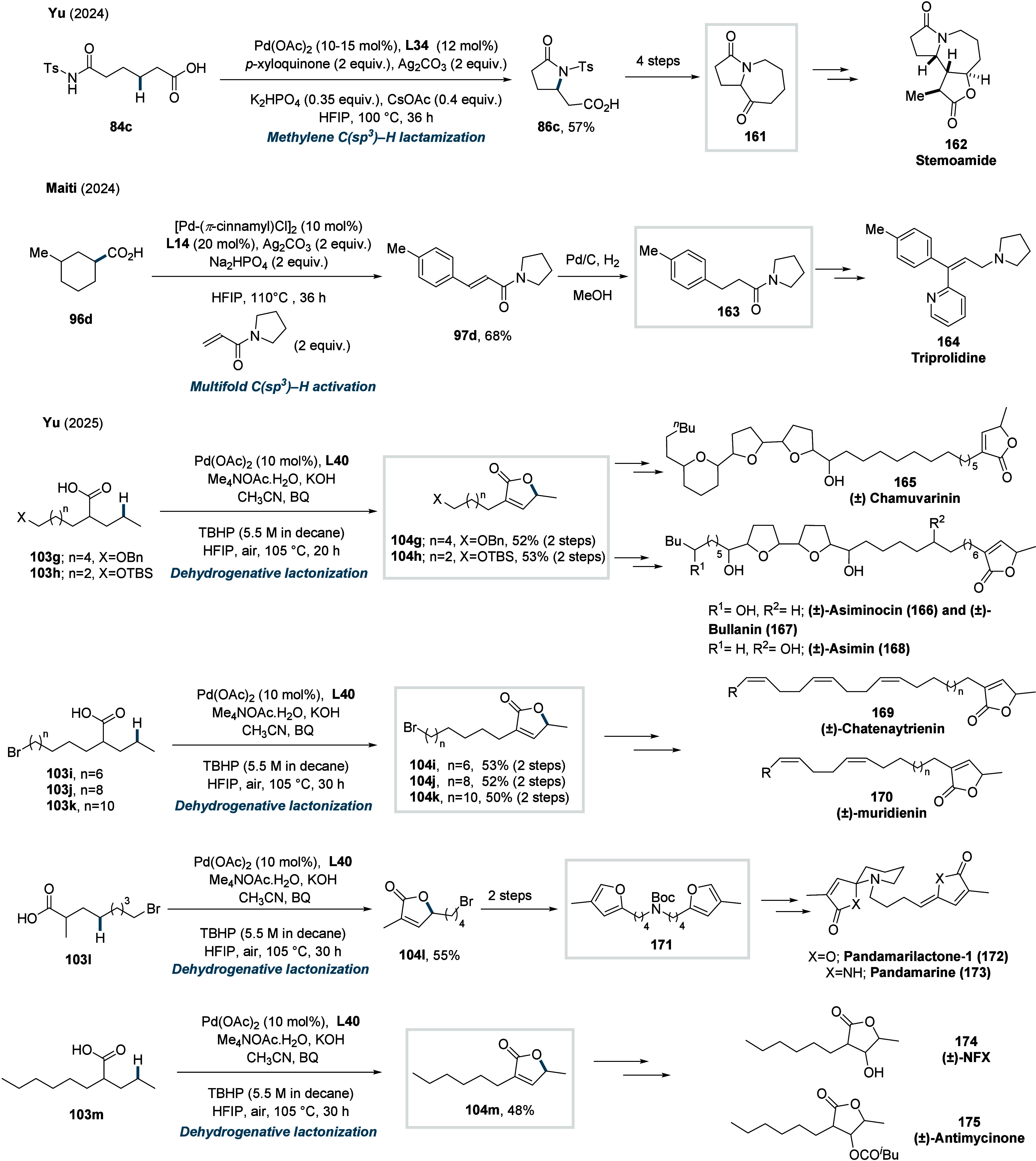
Applications of Miscellaneous Reactions in Formal Synthesis

In 2024, Maiti and co-workers applied their multifold C–H activation protocol to the synthesis of precursor **163**, a key intermediate in the preparation of triprolidine (**164**), an antihistamine drug.^134^ The synthetic sequence commenced with the formation of olefinated arene **97d** from 3-methylcyclohexyl carboxylic acid (**96d**) and acrylamide in 68% yield using their developed methodology. Subsequent reduction of **97d** furnished the target intermediate (**163**).

In 2025, Yu and co-workers reported the synthesis of a structurally diverse array of butenolide intermediates that serve as versatile building blocks for the total synthesis of representative annonaceous acetogenins.^136^ Aliphatic acids bearing a variety of side chain functionalities, including protected alcohol motifs (**103g**–**103h**) and bromine substituents (**103i**–**103k**), were prepared in a single step via LDA mediated alkylation. Application of the standard butenolide synthesis protocol to these substrates enabled efficient access to the corresponding butenolide intermediates. Specifically, intermediates **104g** and **104h**, en route to (±)-chamuvarinin (**165**), (±)-Asiminocin (**166**), (±)-Bullanin (**167**), and (±)-Asimin (**168**) were obtained in 52% and 53% overall yields over two steps, respectively. Likewise, brominated substrates (**103i**–**103k**) furnished the corresponding butenolides (**104i**–**104k**) which serve as intermediates for the synthesis of (±)-chatenaytrienin (**169**) and (±)-muridienin (**170**). Aliphatic acid precursor **103l** underwent smooth conversion under the standard conditions to deliver **104l** in 55% yield, which was subsequently transformed into intermediate **171**. This intermediate constitutes a key precursor in the total syntheses of (±)-pandamarilactone 1 (**172**) and (±)-pandamarine (**173**). The methodology enabled access to synthetic intermediate **104m** for (±)-NFX (**174**) and (±)-antimycinone (**175**) in one step from the commercially available acid precursor **103m** in 48% yield. Additionally, the authors reported a two-step synthesis of a racemic metabolite from *Pterogorgia* spp., as well as a one-step synthesis of an anti-HIV drug intermediate.

### 5.2 Applications of C­(sp^3^)–H Functionalization in the Total and Formal Synthesis of Natural Products and Bioactive Compounds via HAT and Other Pathways

#### 5.2.1 Applications of Carbon–Oxygen Bond Forming Reactions in Total Synthesis

In 2016, Du Bois and co-workers demonstrated the utility of their copper-catalyzed lactonization protocol in the synthesis of phyllodulcin (**176**), a dihydroisocoumarin natural product (Scheme 80).^152^ Benzoic acid precursor **105m** was subjected to the standard lactonization conditions to afford lactone **106m** in 51% yield, and subsequent removal of the tosyl protecting group furnished the target compound.

**Scheme 80:**
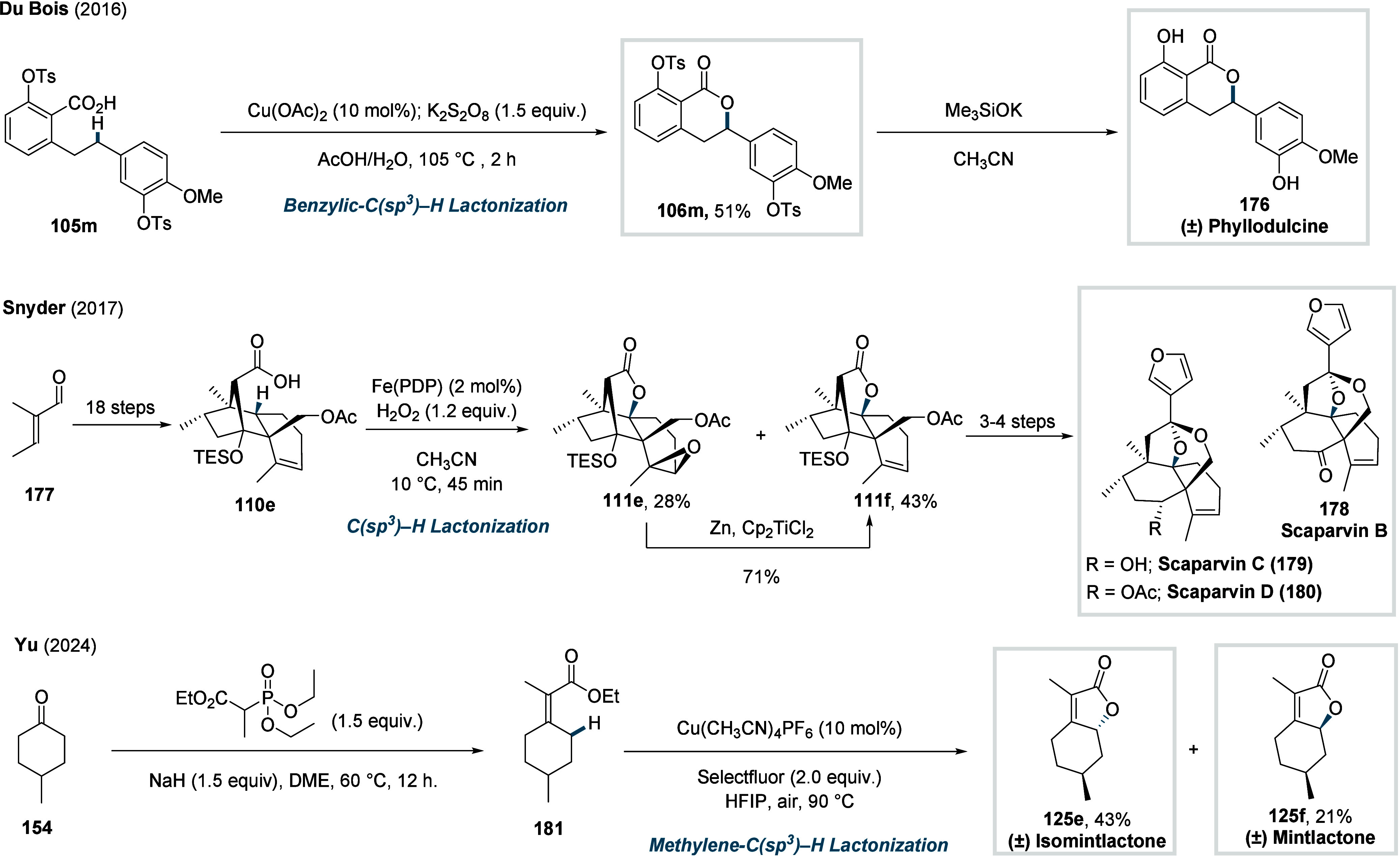
Applications of C–O Bond Forming Reactions in Total Synthesis via HAT

Snyder and co-workers reported the total syntheses of scaparvins B (**178**), C (**179**), and D (**180**) utilizing a late-stage tertiary C­(sp^3^)–H oxidation strategy.^188^ The lactonization precursor **110e** was prepared in 18 steps from **177**. Treatment of **110e** with Fe­(PDP) in the presence of H_2_O_2_ enabled oxidative lactonization to furnish lactone **111f** in 43% yield, along with the undesired epoxide **111e**. Subsequent reductive conversion of **111e** using Cp_2_TiCl_2_ and Zn provided **111f** in 71% yield. Final elaboration of **111f** delivered scaparvins B, C, and D.

In 2024, the Yu group applied their copper-catalyzed C­(sp^3^)–H lactonization protocol to the synthesis of (±) mintlactone (**125e**) and (±) isomintlactone (**125f**), two monoterpenic lactones isolated from the essential oils of several *Mentha* species.^153^ The synthesis commenced with a Horner–Wadsworth–Emmons olefination of 4-methylcyclohexanone (**154**) to furnish ester **181**, the lactonization precursor. Subjecting **181** to the standard copper-catalyzed conditions furnished the corresponding lactone products **125e** and **125f** in 43% and 21% yield, respectively.

In 2024, Wang et al. showcased the application of their enantioselective γ-C­(sp^3^)–H lactonization protocol to the one-step synthesis of optically pure natural products **128g** and **128h**.^180,189,190^ Starting from the corresponding parent carboxylic acid precursors (**39g** and **39h**), the target lactones were obtained in 51% and 53% yield, respectively (Scheme 81).

**Scheme 81:**
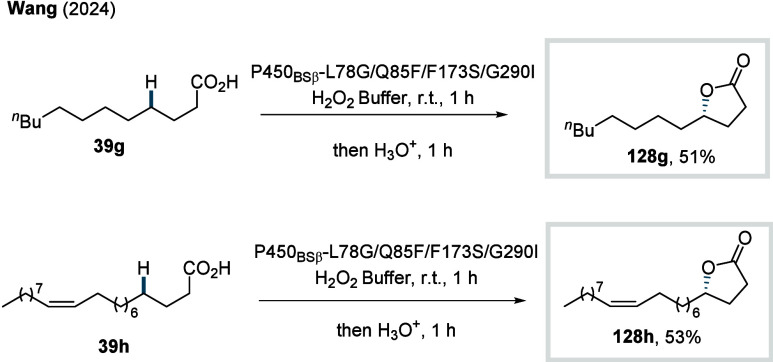
Applications of C–O Bond Forming Reactions in Total Synthesis via Other Pathways

#### 5.2.2 Applications of Carbon–Oxygen Bond Forming Reactions in Formal Synthesis

In 2023, Costas et al. showcased the utility of their lactonization protocol by accessing lactone **127k** from the parent acid (**126k**) in a single step, achieving 71% yield and 99% ee (Scheme 82).^164^ The precursor **127k** serves as a key synthetic intermediate in the total synthesis of (−) maximizcin (**182**).^191^

**Scheme 82:**
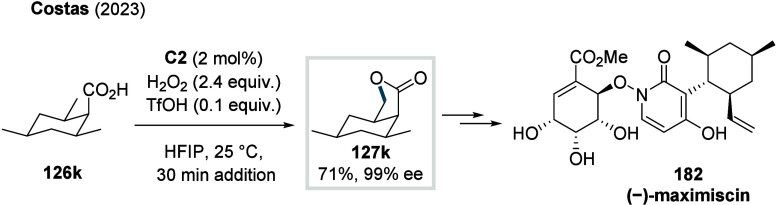
Applications of C–O Bond Forming Reactions in Formal Synthesis

## 6 Conclusion and Outlook

In conclusion, the use of the carboxylic acid group to direct selective C­(sp^3^)–H functionalization processes has received tremendous research interest over the past decades. C–H activation strategies exploiting the weak coordination ability of the carboxylic acid moiety, have enabled a direct conversion of C–H bonds into a wide spectrum of valuable molecular architectures. While the identification of suitable ligand scaffolds and fine-tuning of reaction parameters have been crucial to the success of C–H activation strategies, further advances in reaction conditions and substrate scopes are still needed and substantial progress in these areas is expected in the coming years. While β-C­(sp^3^)–H activation has been achieved for both α-quaternary and α-nonquaternary acids, the development of novel ligand scaffolds is still required to expand the chemical space for γ-C­(sp^3^)–H activation reactions, which to date largely remain limited to β-quaternary acids. A particular emphasis in ligand design should be given on the development of chiral ligands for the enantioselective transformations of linear aliphatic acids, due to the large application potential of such transformations. Extending the C­(sp^3^)–H activation to distal positions of linear acids, such as δ-, and ε-C­(sp^3^)–H bonds represents a formidable challenge. Environmentally benign oxidants such as molecular oxygen or earth-abundant 3d-metal oxides should replace the stoichiometric silver salts that are frequently employed as terminal oxidants. Likewise, electrochemical or photochemical regeneration of the active catalyst constitutes a powerful alternative to traditional redox processes. Given the elevated temperatures typically required in existing methodologies, metallaphotoredox catalysis offers a compelling strategy to overcome this limitation by generating reactive intermediates under mild conditions, which can then be intercepted by C–H-activated species to promote subsequent bond formation, thereby bypassing the sometimes sluggish oxidative addition step. Virtually all advances in carboxylic acid-directed C­(sp^3^)–H activation have been made through palladium catalysis, underscoring the need for cost-effective and earth-abundant 3d-metal catalysts to enable more environmentally benign and economically attractive versions of these methodologies. Beyond sustainability considerations, the application of 3d metals to methylene C–H functionalization may enable mechanistically distinct pathways, owing to their reduced tendency toward β-hydride elimination and their ability to engage in single-electron transfer and rapid radical capture. In parallel to developments in C–H activation, C–H functionalizations through alternative mechanisms such as radical-mediated pathways have unlocked complementary reactivity and selectivity patterns and are also expected to see important further developments in the upcoming years. To date, studies in this direction have largely remained confined to lactonization reactions. This bias can be attributed to the absence of an external coupling partner, which renders the carbon-centered radical intermediate susceptible to rapid intramolecular capture by the pendant carboxylate or to recombination via metal-bound hydroxo ligand transfer, followed by cyclization to form lactones. Mechanistically, these pathways outcompete alternative reactivities of the radical due to the inherent proximity of the reaction partners. In principle, however, the same carbon-centered radical could be diverted toward intermolecular interception by suitably reactive trapping agents, thereby enabling C–heteroatom or C–C bond formation, a reactivity profile that is well precedented in systems lacking a proximal carboxylate functionality. Enabling such divergence will require precise modulation of the catalyst’s electronic structure and steric environment to suppress rapid carboxylate or hydroxo rebound and to extend the lifetime of the radical intermediate, thereby allowing kinetically competitive interception by an external coupling partner. Furthermore, methods capable of efficiently functionalizing C­(sp^3^)–H bonds beyond the γ-position, as well as catalyst tailored for the effective functionalization of aliphatic acids bearing α-hydrogens would be particularly attractive in this field. In light of the substantial progress in the direct C­(sp^3^)–H functionalization of carboxylic acids enabled by the two complementary strategies described in this review, intense research to address the remaining challenges discussed above is expected to deliver further highly valuable synthetic methods in the near future. The level of practicality these methods have already reached is poised to further expand their widespread adoption in various fields, such as target oriented synthesis, and to drastically shorten the synthetic steps required for the synthesis of bioactive molecules and natural products. The suitability of the methods discussed in this review for the generation of compound libraries of diversely functionalized bioactive molecules and for the late-stage functionalization of complex substrates are expected to trigger their adoption for structure–activity relationship (SAR) studies, thereby aiding drug discovery processes.
